# Gene expression changes in the medial prefrontal cortex and nucleus accumbens following abstinence from cocaine self-administration

**DOI:** 10.1186/1471-2202-11-29

**Published:** 2010-02-26

**Authors:** Willard M Freeman, Melinda E Lull, Kruti M Patel, Robert M Brucklacher, Drake Morgan, David CS Roberts, Kent E Vrana

**Affiliations:** 1 Department of Pharmacology, Penn State College of Medicine, Hershey, PA, 17033, USA; 2 Functional Genomics Facility, Penn State College of Medicine. Hershey, PA, 17033, USA; 3 Department of Psychiatry, University of Florida, Gainesville, FL, 32611, USA; 4 Department of Physiology & Pharmacology, Wake Forest University School of Medicine, Winston-Salem, NC, 27157, USA

## Abstract

**Background:**

Many studies of cocaine-responsive gene expression have focused on changes occurring during cocaine exposure, but few studies have examined the persistence of these changes with cocaine abstinence. Persistent changes in gene expression, as well as alterations induced during abstinence may underlie long-lasting drug craving and relapse liability.

**Results:**

Whole-genome expression analysis was conducted on a rat cocaine binge-abstinence model that has previously been demonstrated to engender increased drug seeking and taking with abstinence. Gene expression changes in two mesolimbic terminal fields (mPFC and NAc) were identified in a comparison of cocaine-naïve rats with rats after 10 days of cocaine self-administration followed by 1, 10, or 100 days of enforced abstinence (n = 6-11 per group). A total of 1,461 genes in the mPFC and 414 genes in the NAc were altered between at least two time points (ANOVA, p < 0.05; ± 1.4 fold-change). These genes can be subdivided into: 1) changes with cocaine self-administration that do not persist into periods of abstinence, 2) changes with cocaine self-administration that persist with abstinence, 3) and those not changed with cocaine self-administration, but changed during enforced abstinence. qPCR analysis was conducted to confirm gene expression changes observed in the microarray analysis.

**Conclusions:**

Together, these changes help to illuminate processes and networks involved in abstinence-induced behaviors, including synaptic plasticity, MAPK signaling, and TNF signaling.

## Background

A hallmark of cocaine addiction is continued drug craving and relapse propensity despite long-term drug abstinence. Development of effective cocaine addiction treatments therefore requires therapies that decrease the likelihood of relapse to cocaine abuse in the recovering addict. A central theme of cocaine abuse research is the role of neurobiological changes (*e.g.*, electrophysiology, neurochemistry, neuroanatomy, epigenetic, transcriptomic, proteomic) in the development and maintenance of the addicted behavioral phenotype (i.e., increased drug-seeking and drug-taking).

Cocaine addiction generally starts with recreational use and deteriorates over time into a compulsive and chronically relapsing drug-taking disorder [1]. Stress, environmental cues, and conditioned stimuli have been demonstrated clinically to play a role in cocaine relapse [2-4]. While initiating drug abstinence can be accomplished through in-patient treatment, maintaining cocaine abstinence has proven difficult [5]. In controlled clinical trials, prolonged cocaine abstinence is often achieved by only a minority of patients [6-8]. This may be due to increases in cocaine craving during drug abstinence [9]. Understanding the persistent neurobiological changes that contribute to continued drug craving during abstinence and relapse potential represents an important step towards identifying treatments that reduce the likelihood of relapse [10].

There is a growing understanding of the acute gene/protein expression changes with cocaine administration (either non-contingently or self-administered) that may be important to the development and expression of cocaine-responsive behavior [11], but only a small number of studies have examined whether these changes revert to normal levels or remain altered with cocaine abstinence. Observations of molecular changes persisting into or occurring during this abstinence period provide the opportunity to identify genes and their protein counterparts that could be used as therapeutic targets to decrease relapse liability.

The development of animal models of cocaine abuse and abstinence has led to the identification of rodent behaviors similar to those of human cocaine abusers. Most notably, time-dependent increases in cocaine seeking and taking behaviors have been observed in the rat model of cocaine abuse and enforced abstinence employed in this study [12-14]. Similar observations have been made using other animal models of prolonged abstinence from cocaine [15,16]. Molecular analyses of these models have not only identified changes in gene or protein expression [12,17-19], but have also correlated gene expression with cocaine-responsive behaviors [20,21]. Many of the existing reports used targeted approaches to quantify specific gene and protein expression changes during abstinence from cocaine. Large-scale discovery studies with long-term enforced abstinence following cocaine self-administration are limited and transcriptomic studies, in particular, have not been conducted.

The self-administration paradigm used in this study exhibits increased reinforcing efficacy, drug seeking, and drug taking with at least 7 days, and as long as 100 days, of abstinence after a period of cocaine self-administration [12-14,22]. Examination of mesolimbic structures in these animals is warranted by the roles that these structures, including the medial prefrontal cortex (mPFC) and nucleus accumbens (NAc), play in reward and behavioral responses to stimuli. Both of these brain regions have been implicated in cocaine abuse and withdrawal through imaging [23-25], behavioral [20,21,26], and molecular [12,17-19] studies. We have conducted targeted mRNA and epigenetic analysis from this model previously [12]. The aim of the present study was to extend this initial analysis of mesolimbic dopaminergic terminal regions by providing a genome-wide characterization in both the mPFC and the NAc of rats following 10 days of cocaine self-administration and after increasing periods of enforced abstinence from cocaine (1, 10, and 100 days). Identification of genes persistently altered in expression by cocaine or altered during a period of cocaine abstinence provides insight into the mechanisms involved in the long-term behavioral changes that occur with cocaine abuse and illuminates novel potential new targets for pharmacological intervention.

## Results

### Animals

Behavioral analyses of the rat cocaine self-administration paradigm and time points used in this study have been published previously [12,14,27]. The specific animals used in this study represent an independent set that was not behaviorally tested (e.g. progressive ratio or extinction responding) to avoid any confounding effects of behavioral testing on gene expression. All cocaine self-administering groups were maintained on a continuous access (24 hours/day) discrete trials (DT) schedule for 10 days. Trials were limited to 4 trials per hour (DT4). After 10 days of DT4 responding, animals were subjected to 1, 10, or 100 days of enforced abstinence. The cocaine intake data for the specific animals used in this study is presented in Table 1. No significant differences were observed in the total cocaine intake of each group or the average number of daily injections. The similarity in total cocaine intake and responding between groups minimizes the possibility that exposure to differing amounts of cocaine, or a difference in self-administration behavior, could account for gene expression changes observed. Microarray studies of the mPFC were conducted on naïve, 1-day abstinent, and 100-days abstinent animals (n = 6/group). Microarray studies of the NAc were conducted on naïve, 1-day abstinent, and 10-days abstinent animals (n = 5/group) from the same cohort. For confirmatory qPCR analyses of gene expression levels, a larger number of samples were tested, including those included in microarray studies. Additionally, while microarrays were conducted on samples from three time points, all four time points were used for these confirmation studies (naïve, n = 11; 1-day, n = 6; 10-days, n = 7; and 100-days, n = 8) to provide finer temporal resolution.

**Table 1:** Cocaine intake data for samples.

	Arrays				qPCR Confirmation		
	
	Group	N	Average Total Intake	Average Injections Per Session	N	Average Total Intake	Average Injections Per Session
**mPFC**	Naïve	6	-*	-	11	-	-
	
	1-day Abstinence	6	934 ± 108 mg/kg	62 ± 7	6	934 ± 108 mg/kg	62 ± 7
	
	10-days Abstinence				7	950 ± 105 mg/kg	63 ± 7
	
	100-days Abstinence	6	856 ± 42 mg/kg	57 ± 3	8	855 ± 39 mg/kg	57 ± 3

**NAc**	Naïve	5	-*	-	11	-	-
	
	1-day Abstinence	5	932 ± 118 mg/kg	57 ± 28	6	934 ± 108 mg/kg	62 ± 7
	
	10-days Abstinence	5	951 ± 116 mg/kg	63 ± 8	7	950 ± 105 mg/kg	63 ± 7
	
	100-days Abstinence				8	855 ± 39 mg/kg	57 ± 3

* Naïve animals were not exposed to cocaine, and therefore have zero intake. There were no significant differences in cocaine intake or number of injections per session between groups. "Arrays" indicates animals used for microarray analysis and "qPCR Confirmation indicates animals used for confirmatory qPCR analysis. All of the samples from the array analysis were included in the qPCR confirmation. Data is presented as Mean ± S.D.

### Microarray analysis

**Figure 1:**
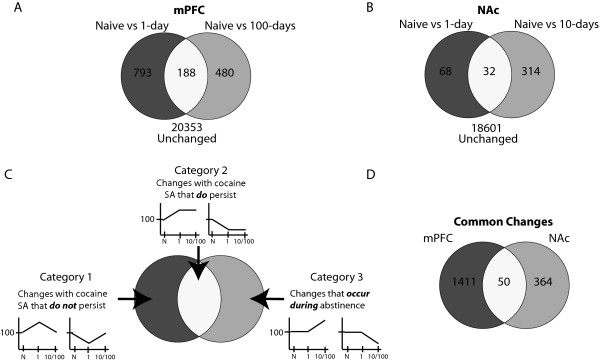
**Analysis of expression changes from the microarray studies**. Venn diagrams illustrate the changes identified by microarray analysis of naïve animals, and animals following 1 and 10 or 100 days of abstinence. (A) In the mPFC, 21,814 probes were detected as present. Of these, 1,461 were changed by greater than 1.4 fold with p < 0.05 (ANOVA) and can be split into three categories based on expression profile: changed between naïve and 1-day (793), naïve and 100-days (480) or both (188). (B) In the NAc, 19,015 probes were detected as present. Of these, 414 were changed by greater than 1.4 fold with p < 0.05 (ANOVA), and can be split into the same three categories: changed between naïve and 1-day (68), naïve and 10-days (314) or both (32). (C) The three categories of gene expression changes can be segregated into (1) changes that occur with cocaine self-administration (SA) that do not persist, (2) changes that occur with cocaine SA that *do *persist, and (3) changes that *occur during *abstinence. Small graphs indicate the expression profile of changes in each category, where the x-axis represents group (naïve, 1-day and 10- or 100-days abstinent) and the y-axis represents percent (%) of naïve expression. (D) When comparing the gene expression changed between the mPFC and NAc, a majority (97%) are unique to one brain region or the other. Only a small fraction (50) is changed in both brain regions.

In the mPFC, gene products corresponding to a total of 21,814 probes (of the 44,000 total probes on the arrays) were confidently detected, based on signal intensity at a fixed value above background levels. In the NAc analysis, mRNAs for a total of 19,015 probes were detected. Differentially expressed genes were identified through a combination of statistical significance (p < 0.05, One-way ANOVA between groups) and a fold change filter of =1.4 fold change (Figure 1A&1B). This illuminated 1,461 gene expression changes in the mPFC (representing 6.7% of the total mRNA species detected) and 414 gene expression changes in the NAc (2.2% of the total detected). These changes demonstrated three types of temporal profiles (Figure 1C). Category 1 changes are those that occurred with cocaine self-administration (either up- or down-regulated at 1-day of abstinence), but that *did not persist *into longer periods of abstinence (10- or 100-days). A majority of the mPFC gene expression changes belonged to this category (793 of 1,461); however, only a small fraction of changes in the NAc (68 of 414) exhibited this expression pattern. Category 2 changes are those that occurred with cocaine self-administration and persisted into periods of abstinence (changed at 1-day and *remained *changed with enduring abstinence). A small number of gene expression changes from each brain region (188 mPFC; 32 NAc) belonged to this category. Finally, category 3 changes are those that did not occur with cocaine self-administration, but changed *during *the period of abstinence (not changed at 1-day, but changed at 10- and/or 100-days). Approximately 33% of the changes in the mPFC (480 of 1,461) and 76% of the changes in the NAc (314 of 414) belong to this category. A comparison of the genes changed at any time point between the mPFC and NAc reveals that a limited number of changes were observed in both brain regions (Figure 1D). Of the total of 1875 gene expression changes, less than 3% were detected in both the mPFC and the NAc. A full list of gene expression changes is presented in Additional File 1: Table S1, and has been uploaded to the Gene Expression Omnibus online database.

### Confirmatory qPCR

qPCR analysis was used to confirm a subset of gene expression changes observed in the microarray analyses. Genes chosen for qPCR confirmation were selected based on ontological classes with potential roles in drug-induced changes in the brain. Additional genes were included in the confirmation studies based on previous reports of cocaine-responsive gene expression (see Additional File 2: Table S2, for a full list and gene expression assay information).

**Figure 2:**
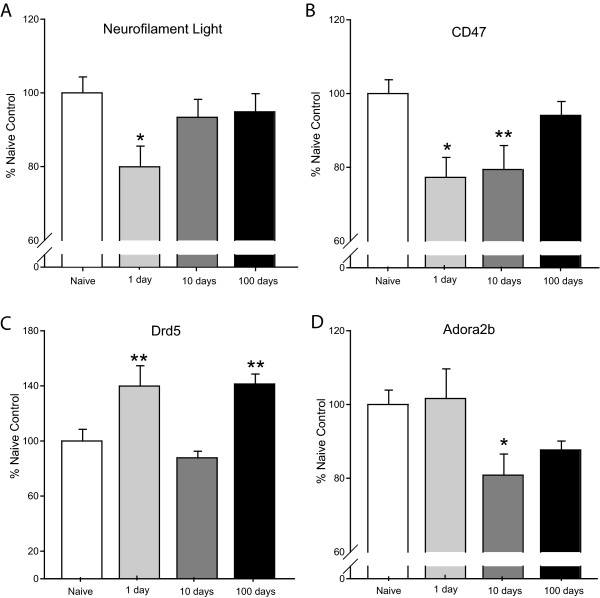
**Confirmed changes in mPFC gene expression**. Four changes in gene expression were confirmed in the mPFC with qPCR analysis. (A) Neurofilament light is decreased by 20% at 1-day of abstinence (p < 0.05). (B) CD47 expression is decreased from naïve at 1-day (23%, p < 0.05) and 10-days of abstinence (21%; p < 0.01). (C) Dopamine receptor D5 (Drd5) expression is increased from naïve at 1-day (40%; p < 0.01) and 100-days (41%; p < 0.01). (D) Adenosine A2B receptor (Adora2b) expression is decreased from naïve at 10-days of abstinence (20%; p < 0.05). Statistical analysis was performed by one-way ANOVA with Student-Newman-Keuls post hoc testing; * p < 0.05, ** p < 0.01.

Differentially regulated genes confirmed in this study (13 total; 4 mPFC, 9 NAc), belong to each of the 3 categories described above (Figure 2). In the mPFC, neurofilament light (Nefl) was the only category 1 change, with a 20% decrease in the mPFC at 1-day of abstinence (Figure 2A). Category 2 changes included a 23% decrease in levels of CD47 at 1-day that persisted through 10-days of abstinence (21%), and an increase in levels of dopamine receptor D5 (Drd5) at 1-day (40%) and 100-days of abstinence (41%) (Figure 2B&2C). Finally, levels of adenosine A2B receptor (Adora2b) were unchanged by cocaine administration, but were reduced by 20% after 10-days of abstinence (Category 3 change) (Figure 2D).

**Figure 3:**
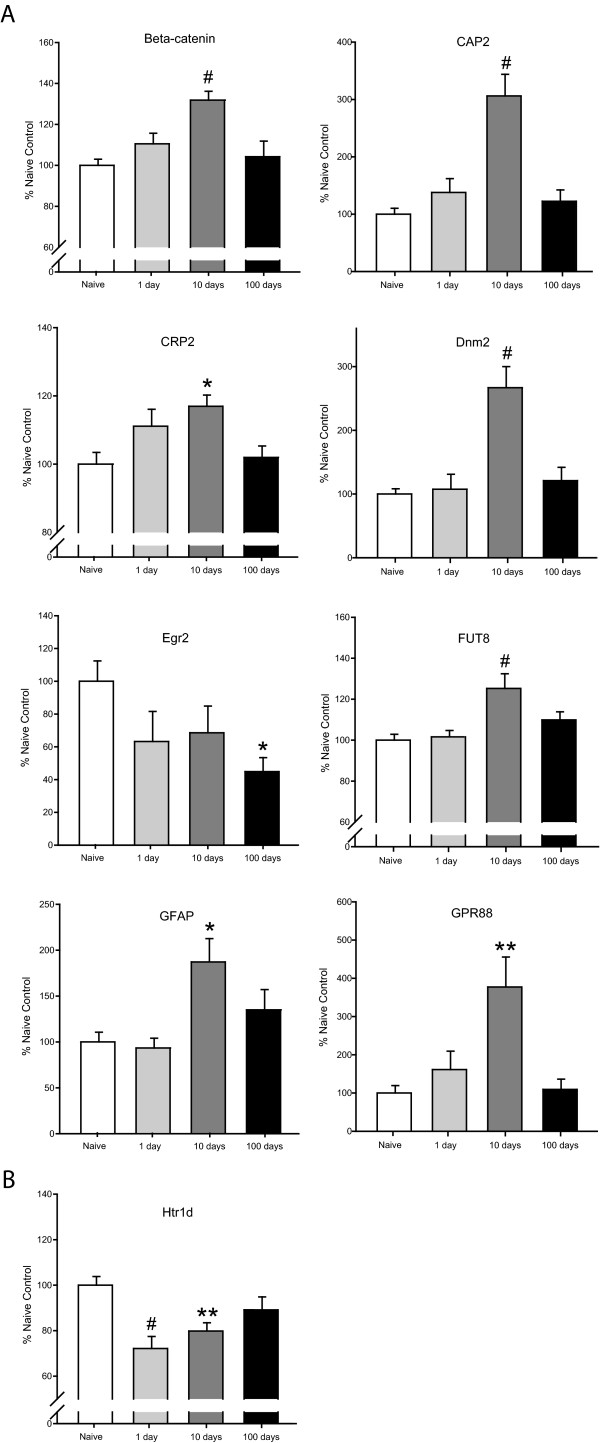
**Confirmed changes in NAc gene expression**. Nine changes in gene expression were confirmed in the NAc with qPCR analysis in this study. (A) Eight of the changes have the profile of changing in expression *during *abstinence (either at 10 or 100-days of abstinence). These genes are beta-catenin (increased by 32% at 10-days; p < 0.001), adenylate cyclase-associated protein 2 (Cap2; increased by 206% at 10-days; p < 0.001), cysteine-rich protein 2 (Crip2; increased 17% at 10-days; p < 0.05), dynamin 2 (Dnm2; increased 167% at 10-days; p < 0.001), early growth response 2 (Egr2; decreased by 55% at 100-days; p < 0.05), fucosyltransferase 8 (Fut8; increased 25% at 10-days; p < 0.01), glial fibrillary acidic protein (GFAP; increased 87% at 10-days; p < 0.05), and G-protein coupled receptor 88 (Gpr88; increased 277% at 10-days; p < 0.01). (B) 5-hydroxytryptamine receptor 1d (Htrd1) is decreased by 28% at 1-day (p < 0.001) and by 20% at 10-days of abstinence (p < 0.01). Statistical analysis was performed by one-way ANOVA with Student-Newman-Keuls post hoc testing; * p < 0.05, ** p < 0.01, #p < 0.001.

In the NAc, nine genes were confirmed, most of which displayed a category 3 profile of changing *during *abstinence. These include beta-catenin (32% increase at 10-days), adenylate cyclase-associated protein 2 (Cap2; 206% increase at 10-days), cysteine-rich protein 2 (Crip2; 17% increase at 10-days), dynamin 2 (Dnm2; 167% increase at 10-days), early growth response 2 (Egr2; 55% decrease at 100-days), fucosyltransferase 8 (Fut8; 25% increase at 10-days), glial fibrillary acidic protein (GFAP; 87% increase at 10-days), and G-protein coupled receptor 88 (Gpr88; 277% increase at 10-days) (Figure 3A). The 5-hydroxytryptamine receptor 1d (Htr1d), which was decreased at 1-day (28%) and remained decreased at 10-days (20%) represents the only confirmed category 2 change (Figure 3B).

Consistent with our previous findings in this animal model and at the same time points [12], the microarray analysis performed in this study detected significant changes in activity-related cytoskeletal associated protein (Arc), cocaine and amphetamine-related transcript (CART), early growth response 1 (Egr1), FBJ osteosarcoma oncogene (Fos), neuropeptide Y (NPY), and nuclear receptor subfamily 4a1 (Nr4a1) transcript levels. Prior to the microarray analysis, we had examined these genes by qPCR based on their known responsiveness to cocaine and have already reported these results [12]. Persistent decreases (Category 2 or 3) in Arc, Fos, and Nr4a1 were observed in both the mPFC and NAc. Category 1 changes in CART, NPY (both increased at 1-day) and Egr1 (decreased at 1-day) in the mPFC were also confirmed by qPCR (Table 2).

**Table 2:** Gene expression changes reported previously

			Expression Level (% of Naïve Control)		
	**Gene Name**	**Gene ID**	**Nv1**	**Nv10**	**Nv100**

**mPFC**	Activity-related cytoskeleton-associated protein	Arc	43.9 ± 14**	76.8 ± 12	51.8 ± 10**
	
	Cocaine and amphetamine regulated transcript	CART	725.2 ± 130**	191.9 ± 55	190.3 ± 86
	
	Early growth response 1	Erg1	59.6 ± 9.2*	74.7 ± 15	74.5 ± 5.3
	
	FBJ osteosarcoma oncogene	Fos	41.7 ± 12**	58.1 ± 11**	53.1 ± 6.8**
	
	Neuropeptide Y	Npy	158.4 ± 21^#^	94.5 ± 9.5	91 ± 5.2
	
	Nuclear receptor subfamily 4, group A, member 1	Nr4a1	35.1 ± 11**	53.4 ± 12*	49.4 ± 7.7*

**NAc**	Activity-related cytoskeleton-associated protein	Arc	83.5 ± 22	73.7 ± 12	49.5 ± 6.4*
	
	FBJ osteosarcoma oncogene	Fos	53.9 ± 16**	60.3 ± 6.7**	51.7 ± 62**
	
	Nuclear receptor subfamily 4, group A, member 1	Nr4a1	65.7 ± 20	63.4 ± 11	48.1 ± 5.1*

Data is presented as mean ± standard error of the mean (SEM), ANOVA with Student-Newman-Keuls post hoc testing; * p < 0.05, ** p < 0.01, # p < 0.001. Data previously reported in [12].

### Ontological and Network analysis

Analysis of the Gene Ontology (GO) categories of changes in each brain region identified a number of molecular functions significantly regulated with cocaine self-administration and abstinence. The molecular functions altered were anatomically distinct. In the mPFC, the most-represented class, consisting of ~ 25% of the changes identified by microarray analysis, was protein serine/threonine kinase activity, followed closely by structural components of the ribosome and monovalent inorganic cation transporter activity (~ 15% of the total changes each). The most represented classes among the changes in NAc gene expression were hydrolase activity (~ 40%), and phosphoric ester hydrolase activity (~ 10%).

Network analysis, conducted using Ingenuity Pathways Analysis software (Ingenuity Systems, Redwood City, CA), was performed to determine the relationships between confirmed gene expression changes from this study and previously reported changes from this model [12]. To complement the GO analysis, additional analysis was conducted to determine whether these genes are implicated in specific pathways and/or biological functions and diseases. Only those genes confirmed by qPCR were used in this analysis. In the mPFC, these molecules comprise a network of interactions involved in synaptic plasticity, calcium signaling, and mitogen activated protein kinase/extracellular signal-regulated kinase (MAPK/ERK) signaling. In the NAc, all 12 molecules were components of a network of interactions with members linked to the neuronal cytoskeleton, glial cells, and Wnt and tumor necrosis factor (TNF) signaling functions.

## Discussion

This study represents the first microarray analysis of mesolimbic gene expression following long-term enforced abstinence from cocaine self-administration. Transcriptomic studies of cocaine-induced gene expression changes have been conducted, but these have focused on non-contingent cocaine administration and no or limited (~ 1 day) abstinence. The work conducted in the present study used a model with well-characterized behavioral changes during periods of abstinence, and used animals not subjected to behavioral testing during abstinence (e.g. progressive ratio or extinction responding) so that the gene expression changes observed are free from the effects elicited by behavioral testing conducted before sacrifice. Additionally, it is important to note that all groups (1, 10, and 100 days of abstinence) self-administered equivalent amounts of cocaine over the 10 days of discrete trial cocaine self-administration. This time-course analysis of gene expression allows for discrimination of gene expression changes associated with increased drug seeking (10 and 100 days of abstinence) from those that occur with cocaine self-administration, but do not persist for as long as increased drug seeking and taking (1 day of abstinence).

The literature describes a number of neurobiological changes (e.g. altered gene/protein expression, neurotransmitter levels, epigenetic events) with different models of cocaine abuse, (for a review see [11,28]). Whether these changes persist into periods of abstinence, however, has generally not been determined. In this study, gene expression changes that occurred as a result of cocaine self-administration and abstinence segregated into three categories of expression patterns. Category 1 changes were defined as those that occur with cocaine use, but *do not *persist into periods of abstinence. These were observed as changes only between naïve animals and 1-day abstinent animals. After only 1 day of abstinence, an increase in drug seeking and drug taking is not observed [12], so the changes in gene expression observed at this point may be necessary, but are not sufficient, to cause the incubated phenotype and are primarily due to exposure to cocaine. Category 2 changes are those that occur with cocaine use that *persist *with periods of abstinence. These were observed to be altered in the comparisons between naïve and 1-day abstinent animals *and *between naïve and 10- or 100-day abstinent animals. These alterations may result from cocaine exposure, but do not return to naïve levels with cessation of the cocaine stimulus. The persistence of these changes may indicate their potential role in the development (10 days) or maintenance (100 days) of abstinence-persistent increases in drug seeking and drug taking behaviors. Category 3 changes consist of genes that were unchanged with cocaine use, but are altered *during *the abstinence period. While not immediately affected by cocaine exposure, this set of changes may result from initiation or continuation of abstinence. These may function synergistically with other (category 2) changes to contribute to the development of increased drug-seeking and -taking.

### mPFC

The mPFC mediates executive function and decision making processes and is therefore a key neuroanatomical region in addictive behaviors [29,30]. In response to cocaine administration, changes in metabolic activity, neurotransmitter systems, and gene or protein expression occur in the mPFC (for a review see [28]). In this study, a large number of gene expression changes were observed in the mPFC both as a result of cocaine self-administration and with subsequent enforced abstinence. Most of these changes occurred as a direct result of the cocaine self-administration (981) and a majority (793) returned to cocaine-naïve levels with cessation of cocaine self-administration. As expected many changes (category 1) in gene expression require continued cocaine stimulus to remain altered, and return to normal levels after the stimulus is removed. A subset of genes (188) remained changed after 100-days of enforced abstinence. Persistence of gene expression changes with abstinence (category 2) requires maintenance via other mechanisms. Epigenetic changes occur in response to cocaine, and may constitute a regulatory mechanism for persistent changes in gene expression [12,31]. Changes (480) that do not occur during cocaine self-administration, but are induced with abstinence (category 3) may reflect the withdrawal of the cocaine stimulus and development of the incubated phenotype.

Altered gene expression of Adora2b, Arc, CART, Cd47, Drd5, Egr1, Fos, Nefl, NPY, and Nr4a1 were confirmed by qPCR. We have previously described altered expression of Arc, CART, Egr1, Fos, NPY, and Nr4a1 in these samples in a directed study of genes with known relevance to drug abuse [12]. A number of the qPCR confirmation analyses that did not reach statistical significance demonstrated expression profiles similar to those observed in the microarray. This may reflect the effects of neuroanatomical complexity on quantitation of gene expression endpoints and the inclusion of larger numbers of animals in the confirmatory experiments.

This work identified altered expression of two G-protein coupled receptors (GPCRs; Adora2b, Drd5), a cell-surface signaling molecule (CD47), and a component of the neuronal cytoskeleton (Nefl). Increased Drd5, and signaling through this receptor, have been reported to decrease responsiveness to cocaine [32,33]. Similarly, adenosine signaling has been implicated in drug addiction. Specifically, activation of Adora2b receptors attenuates cocaine-conditioned place preference [34]. Although the mechanisms underlying these effects are unclear, Drd5 signaling is implicated in neuronal activities including long-term potentiation (LTP; [35]), and reinforcement learning [36], while both Drd5 [37] and Adora2b [38] appear to affect Ca^2+ ^dynamics.

The decrease in CD47 expression in this model is a novel observation and is of interest due to its function in neuronal development [39,40]. Nefl functions in cytoskeletal organization and cell-surface receptor remodeling [41,42], which may be impaired with the observed decrease in expression at 1-day of abstinence. Previously, changes in protein levels and post-translational modifications of Nefl, and other neurofilament isoforms, have been reported with cocaine, morphine, alcohol, and nicotine administration [43-45].

We have previously reported a directed analysis of immediate early genes (IEGs; Arc, Egr1, Fos, and Nr4a1) and neuropeptides (NPY and CART) in this animal model [12]. These genes were also identified in the current discovery microarray analysis, providing increased confidence in the microarray findings. These genes play important roles in a number of neuronal processes including learning and memory [46,47], synaptic plasticity [48-50], Ca^2+ ^signaling [51,52], and MAPK signaling [51].

Network analysis was conducted using the set of confirmed mPFC gene expression changes, and revealed that Cart, NPY, Nr4a1, Fos, Egr1, Adora2b and Drd5 all interact (directly or indirectly) with the MAPK/ERK pathway. While altered expression of MAPK/ERK pathway elements was not detected in this study, changes in expression and activity levels of MAPK/ERK genes have been reported (for a review see [28]) and this pathway is thought to play an important role in drug-induced changes in the brain [53,54]. Regulators of Ca^2+ ^dynamics were also identified in the network analysis. The changes in Drd5, Adora2b, CD47 and CART expression may indicate a decrease in intracellular Ca^2+ ^signaling that occurs with cocaine self-administration and persists into periods of abstinence [40,52,55,56].

Additionally, the gene expression changes identified indicate that synaptic plasticity may be affected by cocaine self-administration and abstinence. Persistent reductions in levels of CD47 and Arc, and inductions in levels of Drd5 and NPY suggest altered synaptic plasticity process involved in memory formation and removal of old memory traces, respectively [50,57,58]. A potential reduction in synaptic plasticity in the mPFC with cocaine self-administration/abstinence is hypothesized based on levels of CD47, Nefl, Arc, Egr1, and NPY [39,42,48-50]. These data are in agreement with studies of the direct role of psychostimulants on mechanisms of synaptic plasticity, including LTP and LTD, in the mesolimbic system [59,60]. In total, these gene expression changes may contribute to persistently altered synaptic plasticity in the mPFC.

### NAc

The central role of the NAc in psychostimulant reward is well documented [61]. While cocaine exerts common actions on the NAc and mPFC [62], we observed little overlap (50 of the 1875 total gene expression changes (mPFC + NAc)) between these brain regions. The regulated genes common to both brain regions include IEGs reported previously [12], various signaling molecules, and genes involved in cellular metabolism. When the microarray datasets were examined by ontological analysis distinct molecular functions were observed in each brain region. This indicates that the functional changes occurring in the mPFC and NAc may differ and may ultimately play different roles in abstinence-dependent behaviors.

Unlike the mPFC, fewer category 1 and 2 changes were observed in the NAc (100 of 414 total), than category 3 changes (those changed specifically during abstinence) (314). Of the cocaine-induced changes, only 32 persisted into periods of abstinence (category 2), while the remainder returned to pre-cocaine levels. Arc, Beta-catenin, Cap2, Crip2, Dnm2, Egr2, Fos, Fut8, GFAP, Gpr88, Htr1d, and Nr4a1 were all confirmed by qPCR to be differentially expressed. We have previously demonstrated the responsiveness of Arc, Fos and Nr4a1 in this animal model [12].

Published data regarding Cap2, Crip2, Fut8, and Gpr88 in the brain are limited, with no previous reports of cocaine-responsiveness. Crip2 (a LIM-domain protein), Cap2 (an adenylate cyclase-associated protein) and Dnm2 are cytoskeletal function and organization genes [63,64]. Interestingly, Dnm2 is regulated by the transcription factor Arc, also altered in the NAc with cocaine [12,65]. Among the remaining changes, Egr2 and GFAP have been previously demonstrated to be cocaine-responsive [66-68]. Htr1d has been linked with a number of psychiatric disorders [69,70]. Changes in the expression of these genes may also indicate cocaine induced alterations in receptor signaling, glial cell function, and synaptic plasticity.

Beta-catenin, which was increased at 10-days of abstinence in this study, is a well-characterized protein that regulates cell growth as a part of the Wnt signaling pathway. As a part of Wnt signaling, beta-catenin also plays a role in synaptic plasticity [71,72]. In response to chronic cocaine, beta-catenin has been shown to increase in a number of brain regions [73-75]. Fut8, a fucosyltransferase protein, also increased at 10-days of abstinence in this study, has been shown to increase upon Wnt/beta-catenin activation [76], indicating that there may be a coordinated activation of Wnt signaling during periods of abstinence from cocaine.

Network analysis of the confirmed genes in the NAc identified a TNF-centered network. Generally involved in inflammatory processes, TNF has not been historically associated with behavioral responses to cocaine. Studies performed on the effects of cocaine on macrophages have reported that cocaine suppresses LPS-stimulated TNF expression [77,78]. TNF induction was recently demonstrated to reduce conditioned place preference and locomotor sensitization caused by methamphetamine and morphine administration [79]. If TNF does play a role in the behavioral responses to cocaine, these additional genes may represent regulatory and effector elements of a TNF network.

While these reported changes represent new insights into abstinence-induced changes in the brain, localization of these changes to specific cell types is still to be determined. As with other functional genomic and proteomic approaches looking at dissected brain regions, even these specific dissections contain a heterogeneous cellular population. Future molecular neurobiology studies that seek to extend these, and other findings, will need to utilize techniques (e.g. laser capture microdissection and fluorescent *in-situ *hybridization) to localize changes to specific cell types and neuronal networks [80].

## Conclusions

In addition to offering further evidence of long-lasting changes in gene expression following abstinence from cocaine self-administration, these results identify cellular processes that may regulate the development and/or maintenance of incubation of drug-seeking and drug-taking. A number of additional changes in gene expression remain to be examined in future studies, but the results presented here support the finding that persistent shifts in gene expression can last long into abstinence. In the mesolimbic reward pathway, changes in the mPFC may be more pronounced than in the NAc and involve mostly distinct sets of genes. This may indicate different metaplastic processes occur in these brain regions with the development and expression of abstinence-induced behaviors. In the mPFC, changes in MAPK/ERK and calcium signaling and in synaptic plasticity occur. The alterations in the NAc suggest a possible role of Wnt and TNF-mediated signaling in cocaine-associated behaviors. Together the findings of this study highlight a number of pathways and processes in the brain that may play roles in the development and maintenance of abstinence-induced drug seeking and drug taking. A clear understanding of how these novel changes contribute to relapse liability will not only increase our knowledge of the neurobiology of addiction, but will provide targets for therapeutic development.

## Methods

### Cocaine self-administration

The surgical and cocaine self-administration procedures used have been described previously [12,18]. Briefly, male Sprague-Dawley rats (Harlan Inc., IN) were implanted with a chronic indwelling Silastic cannula in the right jugular vein, and trained to self-administer cocaine hydrochloride through exposure to a fixed ratio 1 (FR1) schedule of reinforcement as described previously [12,14]. After establishing a stable daily intake of cocaine (40 infusions within 6 hours for at least 5 days), access conditions were changed to a discrete-trials schedule. During the discrete-trial schedule, rats were given access to cocaine for 10-min discrete trials that were initiated at 15-min intervals. An infusion (1.5 mg/kg/inj) of cocaine was given following a response on the lever, which resulted in illumination of a stimulus light for 20 sec. The lever was retracted and the trial terminated if an injection was collected or if 10 minutes had elapsed. Rats received four discrete trials per hour (i.e., DT4), 24 hours per day, for 10 days. Following 10 days of self-administration, animals were placed in standard polycarbonate cages for 1, 10 or 100 days of enforced abstinence. All research was approved by the Wake Forest University School of Medicine and Penn State College of Medicine Animal Care and Use Committees and conducted according to the Guide for the Care and Use of Laboratory Animals, as promulgated by the National Institutes of Health.

### Dissection

Following 1, 10, or 100 days of deprivation, rats were sacrificed and the brains were rapidly removed and cooled in ice-chilled saline. Brains were then placed in an ice-chilled ASI brain slicer (ASI Instruments, Warren MI). The medial prefrontal cortex (mPFC) and nucleus accumbens (NAc) were collected as described previously [12]. Briefly, the section from Bregma +4.4 to 2.4 mm [81] was cut along the forceps minor and the cortex medial of this cut was collected. This is considered the medial prefrontal cortex, and includes the cingulate area, prelimbic cortex, and medial orbital cortex. The section from + 2.2 to 0.2 mm was cut 0.5 mm on each side of the midline, on a line connecting the tip of the external capsule and the previous cut, on a line connecting the tip of the external capsule and lateral ventricle, and between the ventricle. This dissection includes both the core and the shell of the NAc. See Additional File 3: Figure S1, for schematics of the dissections.

### RNA isolation

Following dissection, tissue samples were stored at -80°C until RNA was isolated. RNA isolation was conducted as described previously [74,82]. Total RNA from cocaine naïve rats and rats following 1, 10, and 100 days of enforced abstinence (following 10 days of DT4 cocaine self-administration as described above) was isolated using Tri-Reagent (Molecular Research Center Inc., Cincinnati, OH) [83]. RNA quantity and quality was then assessed using the Agilent 2100 Bioanalyzer (Agilent, Palo Alto, CA) following further RNA purification using an RNeasy Mini Kit for RNA clean-up (Qiagen Sciences, Maryland).

### Microarray analysis

Microarray analysis was performed in the Penn State College of Medicine Functional Genomics Core Facility according to standard procedures. For the mPFC arrays, naïve, 1-day, and 100-day abstinent samples were used (N = 6). For the NAc arrays, naïve, 1-day and 10-day abstinent samples were used (N = 5; reduced sample number due to removal of samples that did not pass array quality control). Microarrays for the NAc were performed after those for the mPFC, and for the NAc, the 10-day timepoint was used instead of the 100-day timepoint in order to detect important changes that may not last to 100 days of abstinence. The persistence of these changes to 100-days could then be tested with qPCR.

Microarray analysis was performed using the CodeLink Rat Whole Genome Bioarray system (GE Healthcare). Following the manufacturer's protocol, first strand synthesis was performed with 2 μg of RNA as starting material and was followed by second strand synthesis and purification using Qiaquick spin columns (Qiagen, Valencia, CA). T7 reaction buffer, T7 NTPs, 10 mM biotin-11-UTP, and T7 polymerase were then added to the dsDNA for the IVT reaction and incubated at 37°C for 14 hours. The resulting Biotin-labeled cRNA was then purified using RNEasy columns (Qiagen), quantitated, and volume-adjusted for a total of 10 μL. The cRNA was then fragmented and denatured before hybridization for 18 hours at 37°C. Slides were washed and then incubated at room temperature with Alexa Fluor 647 labeled streptavidin for 30 minutes followed by washing.

Micro arrays were scanned on an Axon 4000 B scanner with GenePix4 v4.0 software at a 5 μm resolution at 635 nm with laser power at 100%, PMT voltage at 600 V, focus position 0 μm, and lines to average = 1. Images were then imported into CodeLink Expression Analysis Software v4.1 (GE Healthcare) and initial quality control (positive and negative controls), exclusion of manufacturing defects (MSR spots), background subtraction, and intra-array normalization was performed.

### Data analysis

Following image analysis on CodeLink Expression Analysis Software, microarray data were imported into GeneSpring GX 7.3 (Agilent Technologies) and signal values less than 0.01 were transformed to an intensity of 0.01. Normalization was performed per chip to the 50^th ^percentile, and per gene to the median. Values were then normalized on a per gene basis to the naïve group for each of the two time points (1 and 10, or 1 and 100 days of abstinence). Potential differential expression was determined with a one-way ANOVA (variances not assumed to be equal), p < 0.05 and filtered for 1.4-fold or greater differences in expression in accordance with standards for microarray analysis [84]. The use of a combination of statistical and fold-change cutoffs as opposed to traditional multiple testing corrections (*e.g*., Bonferroni post-hoc testing) produce gene lists with the lowest rate of type I and type II errors [85]. A fold-change cutoff of 1.4 fold was chosen, as this magnitude change is at the lower range of changes historically confirmed by qPCR in this laboratory. Lastly, probe sequences on the array were searched against current rat genome sequences to eliminate any probes for sequences removed from the NCBI database.

### Quantitative PCR (qPCR) analysis of gene expression

cDNA synthesis was performed on total RNA from naïve, 1, 10, and 100-day abstinent animals using Superscript III Reverse Transcriptase (Invitrogen, Carlsbad, CA). 1 μg RNA, 500 ng Oligo (dT), and 10 mM each dNTP, were incubated for 5 minutes at 65°C and then chilled on ice for 2 minutes. 5× First Strand Buffer (250 mM Tris-HCl (pH8.3), 375 mM KCl, and 15 mM MgCl_2_), 5 mM DTT (final concentration), 40 U RNaseOut, and 200 U Superscript III RT were then added. The 20 μl reaction was incubated for 60 minutes at 50°C followed by a final incubation at 70°C for 15 minutes for termination. The resulting cDNA product was quantified and 50 ng of product was used in each subsequent qPCR reaction.

Quantitative PCR was carried out on a real-time detection instrument (ABI 7900HT Sequence Detection System) in 384-well optical plates using TaqMan Universal PCR Master Mix and Assay on Demand primers and probes (Applied Biosystems, Foster City, CA) as described previously [86,87]. This examination used a larger set of animals than the microarray analysis (Table 1). Primer/probe sets used are listed in Additional File 2: Table S2. SDS 2.2.2 software and the 2^-ΔΔCt ^analysis method [88]. were used to quantitate relative amounts of product using β-actin as an endogenous control. Significance from qPCR analysis was determined with SigmaStat 3.5 (SYSTAT Software, Inc.) based on one-way analysis of variance (ANOVA) (p < 0.05) with a *post hoc *Student Newman-Keuls test (p < 0.05).

### Ontological, pathway, and network analysis

Ontological analysis used Gene Ontology (GO) categories and differentially expressed processes or functional categories were determined statistically, as previously described [87] using GeneSpring GX software. This analysis determined the number of genes in a category present on the array and the number of expression changes that would be part of that category by random chance given the number of differentially expressed genes. Results from these analyses were used to compile a list of genes to examine by qPCR. Ingenuity Pathway Analysis (Ingenuity Systems, Redwood City CA) was used for network and pathways analysis of the qPCR confirmed gene expression results.

## Abbreviations

Adora2b: adenosine receptor A2b; Arc: activity-related cytoskeletal associated protein; Cap2: adenylate cyclase-associated protein 2; CART: cocaine and amphetamine-related transcript; Crip2: cysteine-rich protein 2; Dnm2: dynamin 2; Drd5: dopamine receptor D5; DT: discrete trials; Egr1: early growth response 1; Egr2: early growth response 2; Fos: FBJ osteosarcoma oncogene; FR1: fixed ratio 1; Fut8; fucosyltransferase 8; GFAP: glial fibrillary acidic protein; GPR88: g-protein coupled receptor 88; Htr1d: 5-hydroxytryptamine receptor 1d; MAPK/ERK: mitogen activated protein kinase/extracellular signal-regulated kinase; mPFC: medial prefrontal cortex; NAc: nucleus accumbens; Nefl: neurofilament light; NPY: neuropeptide Y; Nr4a1: nuclear receptor subfamily 4a1; TNF: tumor necrosis factor

## Authors' contributions

WMF and KEV generated the experimental design. RMB and KMP conducted the array and RT-PCR analyses, respectively. MEL performed the data analysis, prepared the figures, archived the data, and wrote the manuscript. WMF assisted with data analysis and WMF and KEV contributed to the data interpretation. DM and DCSR (Wake Forest University) developed the animal model used and provided the samples for analysis. All authors read and approved the final manuscript.

## Supplementary Material

Additional file 1 **Microarray expression data**. A full listing of differentially expressed genes identified by microarray analysis. Click here for file

Additional file 2 **Gene expression assay numbers**. Gene symbols, full names and gene expression assay numbers. Click here for file

Additional file 3 **Dissection schematics**. Dissection diagrams. Schematics of the mPFC and NAc dissections are provided using modifications of figures from Paxinos and Watson. Numbered red lines are specific dissection cuts using visible landmarks as described in the methods. The shaded area represents the tissue collected for molecular analysis. Click here for file
