# Supplementary material for: Gene expression changes in the medial prefrontal cortex and nucleus accumbens following abstinence from cocaine self-administration
Source: BMC Neurosci. 2010 Feb 26;11:29. doi: 10.1186/1471-2202-11-29 (PMC2837051; doi:10.1186/1471-2202-11-29)
Supplement: Additional file 1 — Microarray expression data. A full listing of differentially expressed genes identified by microarray analysis. [file 1471-2202-11-29-S1.DOC]

**Supplementary Table 1 Microarray expression data**

**mPFC**

Naïve vs. 1-day

| **Gene** | **Alias** | **Accession #** | **Fold Change** | **Probe ID** |
| --- | --- | --- | --- | --- |
| 3-phosphoinositide dependent protein kinase-1 | Pdpk1 | BF401624 | 1.584 | GE1219148 |
| 5'-3' exoribonuclease 1 (predicted) | Xrn1_predicted | BE116585 | 1.7 | GE1183756 |
| A disintegrin-like and metalloprotease (reprolysin type) with thrombospondin type 1 motif, 9 (predicted) | Adamts9_predicted | BF397277 | 1.505 | GE1303573 |
| Abhydrolase domain containing 14A | Abhd14a | BQ211903 | 1.468 | GE13996 |
| acetoacetyl-CoA synthetase | Aacs | NM_023104 | 1.479 | GE21274 |
| Activating signal cointegrator 1 complex subunit 3-like 1 | Ascc3l1 | AW251633 | 1.407 | GE16005 |
| activity regulated cytoskeletal-associated protein | rg3.1 | NM_019361 | 3.109 | GE20683 |
| acyl-CoA synthetase long-chain family member 5 | Acs5; Facl5 | NM_053607 | 1.638 | GE1168369 |
| adaptor-related protein complex 2, beta 1 subunit | Ap2b1 | NM_080583 | 1.505 | GE1241695 |
| adenylate cyclase 2 | Adcy2 | NM_031007 | 1.521 | GE1174680 |
| AE binding protein 1 (predicted) | Aebp1_predicted | CD371177 | 1.805 | GE1267103 |
| AE binding protein 1 (predicted) | Aebp1_predicted | BM388007 | 1.684 | GE1121701 |
| AGENCOURT_17182824 NIH_MGC_233 Rattus norvegicus cDNA clone IMAGE:7101024 5', mRNA sequence. | Grlf1_predicted | CK359453 | 0.696 | GE1215330 |
| AGENCOURT_17182838 NIH_MGC_233 Rattus norvegicus cDNA clone IMAGE:7104000 5', mRNA sequence. |  | CK356329 | 1.435 | GE1270714 |
| AGENCOURT_17578897 NIH_MGC_232 Rattus norvegicus cDNA clone IMAGE:7124785 5', mRNA sequence. |  | CK477071 | 0.528 | GE1299668 |
| AGENCOURT_17638475 NIH_MGC_235 Rattus norvegicus cDNA clone IMAGE:7107569 5', mRNA sequence. |  | CK481115 | 0.524 | GE1151175 |
| aggrecan 1 | Agc | NM_022190 | 1.581 | GE1109042 |
| aldo-keto reductase family 1, member D1 | Akr1d1 | NM_138884 | 0.513 | GE1253447 |
| Aldo-keto reductase family 1, member E1 | Akr1e1 | BQ203983 | 1.603 | GE1191874 |
| aldo-keto reductase family 7, member A3 (aflatoxin aldehyde reductase) | Afar; Akr7a1 | NM_013215 | 0.622 | GE20245 |
| AlkB, alkylation repair homolog (E. coli) (predicted) | Alkbh_predicted | BQ190341 | 2.089 | GE1240479 |
| AMGNNUC:MRMN3-00020-G5-A mrmn3 (10218) Rattus norvegicus cDNA clone mrmn3-00020-g5 5', mRNA sequence. | Fgf12 | CB697972 | 1.581 | GE1113024 |
| AMGNNUC:MRPE4-00173-C11-A mrpe4 (10380) Rattus norvegicus cDNA clone mrpe4-00173-c11 5', mRNA sequence. |  | CB545887 | 0.669 | GE1125941 |
| AMGNNUC:NRDG1-00003-G3-A nrdg1 (10855) Rattus norvegicus cDNA clone nrdg1-00003-g3 5', mRNA sequence. | Amigo | CB547987 | 1.598 | GE1169415 |
| AMGNNUC:NRDG1-00081-A2-A nrdg1 (10855) Rattus norvegicus cDNA clone nrdg1-00081-a2 5', mRNA sequence. |  | CB720022 | 0.69 | GE1105855 |
| AMGNNUC:NRHY3-00129-F11-A W Rat hypothalamus (10735) Rattus norvegicus cDNA clone nrhy3-00129-f11 5', mRNA sequence. |  | CB557697 | 1.69 | GE1286378 |
| AMGNNUC:NRHY4-00109-D8-A W Rat hypothalamus (10464) Rattus norvegicus cDNA clone nrhy4-00109-d8 5', mRNA sequence. |  | CB615816 | 1.414 | GE1162954 |
| AMGNNUC:NRHY4-00140-E12-A W Rat hypothalamus (10464) Rattus norvegicus cDNA clone nrhy4-00140-e12 5', mRNA sequence. |  | CB544865 | 1.541 | GE1292713 |
| AMGNNUC:NRHY5-00234-A11-A W Rat hypothalamus (10471) Rattus norvegicus cDNA clone nrhy5-00234-a11 5', mRNA sequence. |  | CB583629 | 0.543 | GE1192718 |
| AMGNNUC:NRHY7-00012-C9-A nrhy7 (10850) Rattus norvegicus cDNA clone nrhy7-00012-c9 5', mRNA sequence. |  | CB581649 | 0.684 | GE1109939 |
| AMGNNUC:NRPI4-00007-H7-A W Rat pituitary (10472) Rattus norvegicus cDNA clone nrpi4-00007-h7 5', mRNA sequence. | LOC501920 | CB713751 | 1.529 | GE16205 |
| AMGNNUC:SRPB2-00151-G8-A srpb2 (10220) Rattus norvegicus cDNA clone srpb2-00151-g8 5', mRNA sequence. |  | CB768721 | 1.423 | GE1156033 |
| AMGNNUC:TRQI1-00003-E4-A trqi1 (10576) Rattus norvegicus cDNA clone trqi1-00003-e4 5', mRNA sequence. |  | CB792430 | 0.654 | GE1120413 |
| AMGNNUC:TRYP1-00002-E9-A tryp1 (10582) Rattus norvegicus cDNA clone tryp1-00002-e9 5', mRNA sequence. |  | CB803583 | 0.698 | GE1282363 |
| Amphoterin induced gene and ORF 3 | Amigo3 | AA996630 | 1.517 | GE1197881 |
| Ankyrin repeat and MYND domain containing 2 (predicted) | Ankmy2_predicted | BF560671 | 1.601 | GE1251814 |
| Ankyrin repeat and SOCS box-containing protein 11 (predicted) | Asb11_predicted | BF284816 | 0.41 | GE18260 |
| Anterior pharynx defective 1a homolog (C. elegans) | Aph1a | BF411371 | 1.503 | GE16545 |
| Armadillo repeat containing, X-linked 6 | Armcx6 | AI556651 | 1.459 | GE18727 |
| Arsenic (+3 oxidation state) methyltransferase | As3mt | CK227096 | 0.604 | GE1203640 |
| Arylacetamide deacetylase-like 1 (predicted) | Aadacl1_predicted | CB747046 | 1.55 | GE1269328 |
| Aspartoacylase (aminoacylase) 3 | Acy3 | BF390673 | 1.523 | GE1201462 |
| Ataxin 1 | Atxn1 | AI235475 | 1.561 | GE1305121 |
| ATP synthase, H+ transporting, mitochondrial F0 complex, subunit c (subunit 9) isoform 3 | Atp5g3 | NM_053756 | 1.417 | GE13581 |
| ATP synthase, H+ transporting, mitochondrial F1 complex, epsilon subunit | Atp5e | NM_139099 | 1.6 | GE1202898 |
| ATP/GTP binding protein 1 (predicted) | Agtpbp1_predicted | CK474799 | 1.577 | GE1148288 |
| ATPase family, AAA domain containing 2 (predicted) | Atad2_predicted | BF548328 | 1.403 | GE16600 |
| ATPase, H+ transporting, lysosomal V0 subunit a isoform 2 (predicted) | Atp6v0a2_predicted | AI013500 | 1.439 | GE13762 |
| ATPase, H+ transporting, V1 subunit D | Atp6v1d | NM_199386 | 1.626 | GE1181815 |
| ATPase, Na+/K+ transporting, alpha 3 polypeptide | Atpa1a3 | NM_012506 | 1.734 | GE22092 |
| ATP-binding cassette, sub-family C (CFTR/MRP), member 3 | Mlp2; Mrp3 | NM_080581 | 1.669 | GE13038 |
| ATP-binding cassette, sub-family C (CFTR/MRP), member 5 | Mrp5; Abcc5a | NM_053924 | 1.457 | GE13074 |
| ATP-binding cassette, sub-family C (CFTR/MRP), member 9 | Abcc9 | CB325077 | 1.427 | GE1244631 |
| ATP-binding cassette, sub-family D (ALD), member 4 | Abcd4 | CB615784 | 0.634 | GE1154048 |
| Autocrine motility factor receptor (predicted) | Amfr_predicted | AA800222 | 1.525 | GE12272 |
| Autophagy-related 4B (yeast) | Atg4b | BF286237 | 1.668 | GE18333 |
| basic helix-loop-helix domain containing, class B2 | Dec1; Sharp2; Stra13; Stra14; SHARP-2 | NM_053328 | 1.413 | GE13163 |
| B-cell receptor-associated protein 31 | Bcap31 | H35185 | 1.506 | GE15185 |
| B-cell translocation gene 2, anti-proliferative | An; Agl; Pc3; an-1; Tis21 | NM_017259 | 2.402 | GE20453 |
| Bernardinelli-Seip congenital lipodystrophy 2 homolog (human) | Bscl2 | BF386179 | 1.47 | GE1296575 |
| BMP and activin membrane-bound inhibitor, homolog (Xenopus laevis) | Bambi | NM_139082 | 1.955 | GE18823 |
| BMP-2 inducible kinase | Bmp2k | CB548423 | 0.603 | GE1239201 |
| bone marrow stromal cell antigen 1 | Bst1 | NM_030848 | 1.931 | GE19500 |
| BPY2 interacting protein 1 (predicted) | Bpy2ip1_predicted | BI297161 | 1.602 | GE1231130 |
| brain abundant, membrane attached signal protein 1 | NAP22 | NM_022300 | 1.547 | GE1197450 |
| Brain-enriched guanylate kinase-associated | Begain | BF396927 | 1.42 | GE1146423 |
| Branched chain aminotransferase 1, cytosolic | Bcat1 | BE118063 | 1.499 | GE1136969 |
| Brix domain containing 1 (predicted) | Bxdc1_predicted | BF393904 | 1.684 | GE1300057 |
| BTB (POZ) domain containing 14A | Btbd14a | BF414548 | 0.6 | GE1225273 |
| BTB (POZ) domain containing 4 (predicted) | Btbd4_predicted | CK366351 | 1.548 | GE1204212 |
| Bwk1 leukemia-related gene | Bwk1 | BE113533 | 1.438 | GE1239315 |
| C1q and tumor necrosis factor related protein 1 | C1qtnf1 | AI179236 | 0.688 | GE14583 |
| C1q and tumor necrosis factor related protein 4 (predicted) | C1qtnf4_predicted | AW918311 | 1.586 | GE16897 |
| Cadherin 16 | Cdh16 | AA891858 | 0.655 | GE12623 |
| calcium channel, voltage-dependent, beta 3 subunit | CACH3B | NM_012828 | 1.451 | GE19992 |
| calcium/calmodulin-dependent protein kinase II, beta | Ck2b | NM_021739 | 1.496 | GE20786 |
| calcium-activated potassium channel beta subunit | LOC60591 | NM_022203 | 0.591 | GE22057 |
| Caldesmon 1 | Cald1 | AW532335 | 0.5 | GE1111716 |
| Calmodulin binding transcription activator 2 (predicted) | Camta2_predicted | AI007841 | 1.451 | GE13600 |
| calreticulin | Calr | NM_022399 | 1.659 | GE1171859 |
| CAMP responsive element binding protein 1 | Creb1 | BE114301 | 1.425 | GE22111 |
| Carbohydrate sulfotransferase 2 (predicted) | Chst2_predicted | CA510483 | 0.653 | GE1169712 |
| carcinoembryonic antigen-related cell adhesion molecule 3 | Cgm1; RATCEAA; MGC114355 | NM_012702 | 0.57 | GE19911 |
| Cartilage acidic protein 1 | Crtac1 | BE103216 | 1.487 | GE1152116 |
| casein kappa | Csnk | NM_031562 | 0.495 | GE19584 |
| CASK-interacting protein CIP98 | Cip98 | AW530332 | 1.546 | GE16327 |
| CD276 antigen | Cd276 | BF398424 | 1.559 | GE18597 |
| CD47 antigen (Rh-related antigen, integrin-associated signal transducer) | Cd47 | AW141154 | 2.246 | GE21321 |
| CD74 antigen (invariant polypeptide of major histocompatibility complex, class II antigen-associated) | INVG34 | NM_013069 | 0.603 | GE20152 |
| CDNA BC060737 (predicted) | BC060737_predicted | CK365821 | 0.413 | GE1161677 |
| CDNA clone IMAGE:7303447 |  | CA507368 | 1.437 | GE1192123 |
| CDNA clone IMAGE:7303896 |  | H34951 | 1.424 | GE15391 |
| CDNA clone IMAGE:7324860 | MGC94226 | BU759303 | 1.521 | GE1271845 |
| CDNA clone IMAGE:7375734 |  | CB791260 | 2.28 | GE1124365 |
| CDNA clone MGC:95041 IMAGE:7123253 |  | BE113935 | 1.472 | GE19018 |
| CDW92 antigen | Cdw92 | AI105205 | 2.806 | GE14031 |
| CDW92 antigen | Cdw92 | AA801076 | 1.427 | GE12321 |
| Centaurin, beta 5 (predicted) | Centb5_predicted | AW535349 | 1.563 | GE16403 |
| Centaurin, gamma 1 | Centg1 | AW526039 | 1.559 | GE16248 |
| Chaperonin containing TCP1, subunit 2 (beta) | Cct2 | BG671511 | 1.694 | GE15063 |
| chaperonin subunit 3 (gamma) | MGC72950 | NM_199091 | 1.528 | GE1155823 |
| Checkpoint with forkhead and ring finger domains | Chfr | H31570 | 1.735 | GE17971 |
| Chemokine (C-C motif) receptor 2 | Ccr2 | AA926151 | 0.677 | GE1215586 |
| chemokine binding protein 2 | D6; MGC105345 | NM_078621 | 0.463 | GE22070 |
| chimerin (chimaerin) 1 | Chn1 | NM_032083 | 1.628 | GE21127 |
| cholinergic receptor, muscarinic 4 | M4 | M16409 | 1.571 | GE19670 |
| chondroitin sulfate proteoglycan 3 | neurocan | NM_031653 | 1.527 | GE19775 |
| Chondrolectin (predicted) | Chodl_predicted | BQ209692 | 0.692 | GE1270461 |
| Chromobox homolog 1 (Drosophila HP1 beta) (predicted) | Cbx1_predicted | BF549070 | 1.629 | GE1244875 |
| chymotrypsin-like | Ctrl | NM_054009 | 1.592 | GE13076 |
| Claudin 2 (predicted) | Cldn2_predicted | BF548791 | 0.515 | GE1277445 |
| cocaine and amphetamine regulated transcript | Cart | NM_017110 | 0.275 | GE20347 |
| Coenzyme Q6 homolog (yeast) | Coq6 | BE114317 | 1.487 | GE1100402 |
| Coiled-coil domain containing 37 (predicted) | Ccdc37_predicted | CA509611 | 0.713 | GE1103230 |
| Coilin | Coil | BF398409 | 0.615 | GE1252497 |
| cold shock domain protein A | Yb2; Dbpa; MGC124554 | NM_031979 | 0.618 | GE21940 |
| Complexin 1 | Cplx1 | BI281681 | 1.564 | GE1168262 |
| Creatine kinase, mitochondrial 1, ubiquitous | Ckmt1 | BG377436 | 1.404 | GE1217157 |
| C-type lectin domain family 4, member f | Kclr; Clecsf13; MGC112607 | NM_053753 | 0.624 | GE1221456 |
| C-type lectin domain family 4, member f | Kclr; Clecsf13; MGC112607 | NM_053753 | 0.593 | GE21644 |
| CUE domain containing 2 (predicted) | Cuedc2_predicted | BE095878 | 1.443 | GE17108 |
| CXXC finger 5 | Cxxc5 | H33001 | 1.527 | GE15397 |
| cyclin D1 | Ccnd1 | NM_171992 | 1.447 | GE1299175 |
| Cyclin D3 | Ccnd3 | BI280392 | 1.87 | GE1112083 |
| Cyclin M1 (predicted) | Cnnm1_predicted | BF565344 | 1.569 | GE19407 |
| Cystatin S | CysS | BI302476 | 0.66 | GE1228357 |
| cysteine sulfinic acid decarboxylase | Csad | NM_021750 | 0.641 | GE20793 |
| cytochrome c oxidase subunit VIIb | Cox7b | NM_182819 | 1.511 | GE12579 |
| cytochrome c oxidase, subunit Va | Cox5a | NM_145783 | 1.46 | GE21091 |
| Cytochrome c oxidase, subunit VIa, polypeptide 1 | Cox6a1 | BM387708 | 1.483 | GE1269464 |
| Cytochrome c oxidase, subunit VIIIa | Cox8a | BE116231 | 1.588 | GE1137837 |
| cytochrome P450, family 26, subfamily A, polypeptide 1 | Cyp26a1; Cyp26 | NM_130408 | 1.491 | GE1238576 |
| cytochrome P450, subfamily 3A, polypeptide 3 | CYP; RL33; cDEX; CYP3A1; CYP3A23; MGC108757 | NM_013105 | 0.528 | GE21745 |
| Cytochrome P450-like protein | Loc266761 | BF557139 | 1.448 | GE1104448 |
| DAZ associated protein 1 | Dazap1 | AW918273 | 1.419 | GE16895 |
| DEAD (Asp-Glu-Ala-Asp) box polypeptide 50 | Ddx50 | BF389731 | 1.689 | GE1223116 |
| DEAH (Asp-Glu-Ala-His) box polypeptide 30 | Dhx30 | BI273837 | 1.427 | GE1214453 |
| decorin | MGC94682 | X59859 | 0.652 | GE22148 |
| dihydropyrimidinase-like 3 | Crmp4; TUC-4b | NM_012934 | 0.489 | GE15521 |
| dipeptidylpeptidase 3 | MGC124585 | NM_053748 | 1.617 | GE19551 |
| Discs, large homolog 4 (Drosophila) | Dlgh4 | BI294217 | 1.41 | GE14000 |
| dishevelled, dsh homolog 1 (Drosophila) | dvl-1 | NM_031820 | 1.635 | GE1158415 |
| Dispatched homolog 1 (Drosophila) (predicted) | Disp1_predicted | AW920924 | 0.616 | GE1205510 |
| Disrupted meiotic cDNA 1 homolog (yeast) (predicted) | Dmc1h_predicted | AW522855 | 0.644 | GE1210341 |
| DNA-damage inducible transcript 3 | Ddit3 | BF406173 | 1.633 | GE1287394 |
| dopamine receptor D4 | D4RA | NM_012944 | 0.581 | GE21846 |
| Dpy-19-like 1 (C. elegans) (predicted) | Dpy19l1_predicted | AI408686 | 1.503 | GE15123 |
| D-serine modulator-1 | Dsm-1 | AA944561 | 1.524 | GE12850 |
| Dual endothelin 1, angiotensin II receptor | Dear | CA506902 | 1.627 | GE17632 |
| dual specificity phosphatase 1 | Mkp1; CL100; MKP-1; 3CH134; Ptpn16 | NM_053769 | 2.348 | GE1248349 |
| Dual specificity phosphatase 10 (predicted) | Dusp10_predicted | BF544297 | 2.882 | GE1145916 |
| Dual specificity phosphatase 22 (predicted) | Dusp22_predicted | AI171617 | 1.426 | GE14281 |
| dual specificity phosphatase 5 | Cpg21 | NM_133578 | 1.607 | GE13169 |
| Dual specificity phosphatase and pro isomerase domain containing 1 (predicted) | Dupd1_predicted | AW918423 | 0.495 | GE1286762 |
| Dullard homolog (Xenopus laevis) | Dullard | BF420071 | 1.434 | GE1190940 |
| dynamin 3 | dynamin-2 | NM_138538 | 1.788 | GE1174180 |
| dynein light chain LC8-type 1 | Pin; Dlc8; 8kDLC; Dnclc1; MGC72986 | NM_053319 | 1.408 | GE22022 |
| Dynein light chain roadblock-type 2 (predicted) | Dynlrb2_predicted | AI144946 | 0.597 | GE1271480 |
| Dynein-like protein 2 | DLP2 | BI289861 | 1.596 | GE18522 |
| early growth response 1 | Ngf1; Ngfi; NGFI-A; Krox-24; zif-268 | NM_012551 | 1.793 | GE1278192 |
| Early growth response 3 | Egr3 | BF410052 | 1.634 | GE1265585 |
| early growth response 3 | Egr3 | NM_017086 | 0.653 | GE1245976 |
| EF hand calcium binding protein 2 | Necab2 | NM_133415 | 1.48 | GE1123175 |
| EGF-like module containing, mucin-like, hormone receptor-like sequence 1 | Emr1 | BE100625 | 1.53 | GE1292466 |
| EGF-like-domain, multiple 9 (predicted) | Egfl9_predicted | BF523420 | 1.46 | GE1234017 |
| Electron transferring flavoprotein, alpha polypeptide | Etfa | BE110721 | 1.559 | GE1133029 |
| Elongation factor Tu GTP binding domain containing 2 | Eftud2 | BG377695 | 1.659 | GE1156010 |
| endothelin receptor type B | Etb; Ednra | NM_017333 | 0.697 | GE22139 |
| EST189800 Normalized rat heart, Bento Soares Rattus sp. cDNA clone RHEAN65 3' end, mRNA sequence. |  | AA800303 | 2.75 | GE12284 |
| EST191111 Normalized rat kidney, Bento Soares Rattus sp. cDNA clone RKIAB31 5' end, mRNA sequence. |  | AA848351 | 1.697 | GE1214639 |
| EST206192 Normalized rat ovary, Bento Soares Rattus sp. cDNA clone ROVAZ56 3' end, mRNA sequence. |  | AI011741 | 0.59 | GE1106373 |
| EST207701 Normalized rat spleen, Bento Soares Rattus sp. cDNA clone RSPBF62 3' end, mRNA sequence. |  | AI014146 | 1.584 | GE1185597 |
| EST234536 PC12 cells, untreated, pT7T3Pac, TIGR Rattus sp. cDNA clone RPPAA80 3' end, mRNA sequence. |  | AI317865 | 1.483 | GE1103796 |
| EST348968 Rat gene index, normalized rat, norvegicus, Bento Soares Rattus norvegicus cDNA clone RGIEG58 5' end, mRNA sequence. |  | AW917664 | 0.705 | GE16818 |
| EST350850 Rat gene index, normalized rat, norvegicus, Bento Soares Rattus norvegicus cDNA clone RGIFY73 5' end, mRNA sequence. |  | AW919546 | 0.597 | GE1262370 |
| EST448936 Rat Gene Index, normalized rat, Rattus norvegicus cDNA Rattus norvegicus cDNA clone RGIEO54 3' sequence, mRNA sequence. |  | BF284345 | 1.428 | GE18238 |
| EST452796 Rat Gene Index, normalized rat, Rattus norvegicus cDNA Rattus norvegicus cDNA clone RGIGQ87 3' sequence, mRNA sequence. |  | BF288205 | 0.714 | GE1135602 |
| EST531153 Rat gene index, normalized rat, norvegicus Rattus norvegicus cDNA clone RGIAA90 5' end similar to mitochondrial D-loop nascent strand-like region, mRNA sequence. |  | BM986373 | 0.594 | GE1170372 |
| Eukaryotic translation elongation factor 1 alpha 1 | Eef1a1 | BI292815 | 1.468 | GE1292098 |
| Eukaryotic translation elongation factor 1 alpha 1 | Eef1a1 | AI602726 | 1.409 | GE1187452 |
| eukaryotic translation elongation factor 1 alpha 2 | Ps10; Stnl; RATPS10 | NM_012660 | 1.674 | GE1220113 |
| Eukaryotic translation initiation factor 3, subunit 6 | Eif3s6 | BQ201565 | 1.457 | GE1183777 |
| Eukaryotic translation initiation factor 3, subunit 9 (eta) | Eif3s9 | BF417351 | 1.44 | GE1183175 |
| Eukaryotic translation initiation factor 5A | Eif5a | BQ206344 | 4.794 | GE1217424 |
| Eukaryotic translation termination factor 1 | Etf1 | BE118039 | 1.601 | GE1187137 |
| Eyes absent 2 homolog (Drosophila) | Eya2 | BF386078 | 0.634 | GE1110950 |
| Far upstream element (FUSE) binding protein 1 | Fubp1 | BF407775 | 1.587 | GE17389 |
| Far upstream element (FUSE) binding protein 1 | Fubp1 | BM384976 | 1.474 | GE18013 |
| FBJ murine osteosarcoma viral oncogene homolog | Fos | BF415939 | 2.651 | GE18981 |
| FBJ murine osteosarcoma viral oncogene homolog | Fos | AW915240 | 2.535 | GE16552 |
| F-box protein 46 | Fbxo46 | BF564168 | 0.362 | GE1288733 |
| Fibroblast growth factor receptor substrate 3 | Frs3 | BF416732 | 0.69 | GE1292727 |
| Fibronectin type III and SPRY domain containing 2 (predicted) | Fsd2_predicted | AI714110 | 0.698 | GE1133626 |
| Fibronectin type III domain containing 5 | Fndc5 | AI172165 | 1.431 | GE14330 |
| Fibulin 1 (predicted) | Fbln1_predicted | BG374692 | 1.54 | GE1262496 |
| FK506 binding protein 1b | Fkbp1b | NM_022675 | 1.527 | GE21619 |
| FK506 binding protein-like | Fkbpl | AW435459 | 1.667 | GE1299079 |
| fractured callus expressed transcript 1 | Tim9b | NM_053371 | 1.419 | GE13390 |
| Frizzled homolog 6 (Drosophila) | Fzd6 | BQ194726 | 0.634 | GE16839 |
| FtsJ homolog 2 (E. coli) (predicted) | Ftsj2_predicted | BM386994 | 1.473 | GE1270530 |
| G protein-coupled receptor associated sorting protein 1 | pips | NM_134386 | 1.446 | GE1216304 |
| G protein-coupled receptor kinase 6 | GRK6 | NM_031657 | 1.483 | GE1212717 |
| G protein-regulated inducer of neurite outgrowth 1 (predicted) | Gprin1_predicted | BG373539 | 1.573 | GE1255503 |
| Galactokinase 1 | Galk1 | BQ200512 | 1.504 | GE1124796 |
| Gamma-aminobutyric acid (GABA) B receptor 1 | Gabbr1 | AI171785 | 1.602 | GE14299 |
| Gamma-aminobutyric acid (GABA) B receptor 1 | Gabbr1 | BF410498 | 1.433 | GE1244007 |
| gamma-aminobutyric acid (GABA-A) receptor, subunit beta 3 | Gabrb3 | NM_017065 | 1.688 | GE20314 |
| GIPC PDZ domain containing family, member 1 | Gipc; Rgs19; Rgs19ip1 | NM_053341 | 1.468 | GE1169453 |
| glutamate cysteine ligase, modifier subunit | Glclr | NM_017305 | 1.592 | GE20488 |
| glutamate receptor, ionotropic, N-methyl D-aspartate 1 | NR1; NMDAR1 | NM_017010 | 1.599 | GE22164 |
| Glutamate receptor, ionotropic, N-methyl D-aspartate 2A | Grin2a | BF393097 | 2.002 | GE1111325 |
| glutamate receptor, ionotropic, N-methyl D-aspartate 2B | Grin2b | NM_012574 | 2.481 | GE1205376 |
| glutamate receptor, ionotropic, N-methyl D-aspartate 2D | Grin2d | U08260 | 1.532 | GE21562 |
| glycoprotein 2 (zymogen granule membrane) | Gp2 | NM_134418 | 0.634 | GE1137686 |
| Grainyhead-like 1 (Drosophila) (predicted) | Grhl1_predicted | BF395816 | 1.478 | GE1179109 |
| granzyme M (lymphocyte met-ase 1) | Gzmm | L05175 | 1.722 | GE19593 |
| GTPase, IMAP family member 1 | Gimap1 | BQ200653 | 0.661 | GE1178632 |
| GTPase, IMAP family member 5 | Gimap5 | BF562358 | 1.874 | GE1187028 |
| Guanine nucleotide binding protein (G protein), gamma 12 | Gng12 | BF523372 | 1.498 | GE1302229 |
| guanine nucleotide binding protein, beta polypeptide 2 | Gnb2 | NM_031037 | 1.42 | GE1305917 |
| H2A histone family, member Z | MGC72814; MGC105426 | NM_022674 | 1.495 | GE1221561 |
| heart and neural crest derivatives expressed transcript 1 | eHand; MGC114332 | NM_021592 | 1.54 | GE20749 |
| heat shock 70kDa protein 5 binding protein 1 | Gbp | NM_178021 | 1.586 | GE1154612 |
| heat shock transcription factor 1 | Hsf1 | X83094 | 1.547 | GE22197 |
| Hect domain and RLD 3 (predicted) | Herc3_predicted | BG668746 | 1.427 | GE15176 |
| Hermansky-Pudlak syndrome 1 homolog (human) | Hps1 | CK222076 | 0.691 | GE1158633 |
| Hermansky-Pudlak syndrome 4 homolog (human) (predicted) | Hps4_predicted | BG377981 | 1.438 | GE1291847 |
| HIG1 domain family, member 1B (predicted) | Higd1b_predicted | BQ204324 | 1.406 | GE18969 |
| HIG1 domain family, member 2A (predicted) | Higd2a_predicted | BF407545 | 1.437 | GE12722 |
| high mobility group AT-hook 1 | Hmgi; Hmgiy | X62875 | 1.566 | GE1132911 |
| Histone deacetylase 10 | Hdac10 | CK365614 | 1.468 | GE1293134 |
| HMP19 protein | MGC125201 | CA508410 | 1.463 | GE1301409 |
| Homer homolog 3 (Drosophila) | Homer3 | BI296126 | 1.453 | GE1300462 |
| Hydrocephalus-inducing protein | LOC292017 | BF402637 | 0.714 | GE1158317 |
| Hypothetical LOC292874 (predicted) | RGD1309036_predicted | AA925385 | 2.985 | GE12769 |
| Hypothetical LOC306766 | LOC306766 | AI170668 | 1.502 | GE14246 |
| Hypothetical protein LOC303515 | LOC303515 | BQ195904 | 1.585 | GE1277315 |
| hypothetical protein LOC502414 | LOC502414 | AY380821 | 1.44 | GE15222 |
| Hypothetical protein LOC681153 | LOC681153 | CB577030 | 1.48 | GE1108961 |
| Hypothetical protein LOC685117 | LOC685117 | H32340 | 1.458 | GE1205816 |
| Hypothetical protein LOC688916 | LOC688916 | BF563473 | 0.671 | GE1278983 |
| Hypothetical protein LOC691898 | LOC691898 | BM386732 | 1.463 | GE1198197 |
| hypoxia induced gene 1 | Hig1 | NM_080902 | 1.521 | GE1157086 |
| immediate early response 2 | MGC72578 | BC061717 | 2.138 | GE19059 |
| Immediate early response 5 | Ier5 | BQ193418 | 1.606 | GE1136663 |
| Immunoglobulin superfamily, member 8 | Igsf8 | AA858677 | 1.414 | GE12545 |
| Inhibitor of kappaB kinase beta | Ikbkb | CB716031 | 1.444 | GE1215960 |
| Insulin-like growth factor 1 receptor | Igf1r | BG371510 | 1.452 | GE1128326 |
| Integrin, beta 5 | Itgb5 | BG373383 | 0.705 | GE1146516 |
| Intercellular adhesion molecule 5, telencephalin (predicted) | Icam5_predicted | BE102418 | 1.463 | GE1142134 |
| Interferon-induced protein with tetratricopeptide repeats 1 (predicted) | Ifit1_predicted | BF542737 | 1.608 | GE1237247 |
| interferon-related developmental regulator 1 | Pc4; MGC93482 | NM_019242 | 1.596 | GE20609 |
| interleukin 18 binding protein | Igifbp | NM_053374 | 1.489 | GE1191880 |
| Intimal thickness-related receptor | MGC94555 | AW528127 | 2.182 | GE1203720 |
| Intraflagellar transport 80 homolog (Chlamydomonas) | Ift80 | AW915413 | 1.403 | GE1126033 |
| IQ calmodulin-binding motif containing 1 (predicted) | Iqcb1_predicted | BF287587 | 1.615 | GE1269405 |
| Ischemia related factor vof-21 | LOC259228 | NM_147208 | 1.543 | GE1255997 |
| Isocitrate dehydrogenase 3 (NAD+) alpha | Idh3a | BE109638 | 1.514 | GE17576 |
| jagged 2 | Jag2 | U70050 | 1.452 | GE21012 |
| Jumonji domain containing 2C (predicted) | Jmjd2c_predicted | AW914947 | 0.699 | GE1110615 |
| Jun dimerization protein 2 | Jundp2 | BF416076 | 1.483 | GE1134389 |
| Jun-B oncogene | Junb | NM_021836 | 1.615 | GE1221854 |
| junction plakoglobin | Jup | NM_031047 | 1.538 | GE20979 |
| kalirin, RhoGEF kinase | Duo; Hapip; Kalirin | NM_032062 | 0.655 | GE13496 |
| Karyopherin (importin) beta 1 | Kpnb1 | C06868 | 1.401 | GE13629 |
| Katanin p80 (WD40-containing) subunit B 1 | Katnb1 | CA508369 | 1.559 | GE1164437 |
| Kelch domain containing 3 | Klhdc3 | H34526 | 1.434 | GE1191282 |
| KH domain containing, RNA binding, signal transduction associated 1 | P62; Sam68 | NM_130405 | 1.644 | GE1228372 |
| kidney specific organic anion transporter | Slc21a4; OAT-K1; OAT-K2; rOAT-K1; rOAT-K2; rOAT-K3; rOAT-K5; rOAT-K6; rOAT-K7; rOAT-K8; rOAT-K9; rOAT-K11; rOAT-K13; rOAT-K14 | NM_030837 | 1.757 | GE19518 |
| Kinase D-interacting substance 220 | Kidins220 | AI102262 | 1.46 | GE13910 |
| Kruppel-like factor 4 (gut) | GKLF; MGC93286 | NM_053713 | 2.149 | GE14597 |
| Kruppel-like factor 5 | IKLF; Bteb2; bteb2 | NM_053394 | 1.463 | GE13435 |
| lactate dehydrogenase B | Ldh2 | NM_012595 | 1.456 | GE21898 |
| Ladinin (predicted) | Lad1_predicted | AA799544 | 0.713 | GE12226 |
| latrophilin 1 | CL1BA | NM_022962 | 1.448 | GE1226348 |
| latrophilin 3 | Lec3; Cirl3 | NM_130822 | 1.532 | GE13276 |
| Leucine rich repeat containing 3B (predicted) | Lrrc3b_predicted | BF564482 | 1.523 | GE1115283 |
| Leucine rich repeat neuronal 1 | Lrrn1 | CA505190 | 0.557 | GE1262406 |
| LIM and senescent cell antigen like domains 2 | Lims2 | BF401381 | 0.698 | GE1226578 |
| Ly6/neurotoxin 1 (predicted) | Lynx1_predicted | AI716642 | 1.481 | GE15665 |
| Mammary tumor virus receptor 2 | Mtvr2 | AI236101 | 1.414 | GE14918 |
| MAP/microtubule affinity-regulating kinase 1 | Mark1 | AI176765 | 1.428 | GE21226 |
| MARCKS-like 1 | F52; Mlp; MGC93399 | NM_030862 | 1.496 | GE15735 |
| Matrix metallopeptidase 14 (membrane-inserted) | Mmp14 | BG663104 | 1.544 | GE21893 |
| matrix metallopeptidase 14 (membrane-inserted) | Mt1-mmp | NM_031056 | 1.487 | GE1182623 |
| matrix metallopeptidase 23 | Mmp23 | NM_053606 | 2.084 | GE13039 |
| Mediator of RNA polymerase II transcription, subunit 25 homolog (yeast) (predicted) | Med25_predicted | AA892531 | 1.421 | GE12663 |
| Mediator of RNA polymerase II transcription, subunit 31 homolog (yeast) (predicted) | Med31_predicted | AI717232 | 1.412 | GE1129224 |
| methionine adenosyltransferase II, alpha | Sams2 | BC062394 | 1.465 | GE1225389 |
| Methionine sulfoxide reductase B2 | Msrb2 | BM389859 | 1.579 | GE16734 |
| Methylenetetrahydrofolate dehydrogenase (NADP+ dependent) 1-like (predicted) | Mthfd1l_predicted | BF281848 | 1.445 | GE18057 |
| microtubule-associated protein 1 light chain 3 alpha | MGC105263 | NM_199500 | 1.533 | GE1175605 |
| Microtubule-associated protein 1b | Map1b | H34194 | 1.486 | GE1280554 |
| Microtubule-associated protein 6 | Mtap6 | CA505054 | 1.435 | GE1241110 |
| Mitochondrial carrier homolog 2 (C. elegans) (predicted) | Mtch2_predicted | AW918605 | 1.409 | GE16935 |
| Mitochondrial protein, 18 kDa | MGC94604 | AA943734 | 1.445 | GE12796 |
| Mitochondrial ribosomal protein L51 (predicted) | Mrpl51_predicted | AW918154 | 0.599 | GE16874 |
| Mitochondrial ribosomal protein L52 (predicted) | Mrpl52_predicted | AI409180 | 0.528 | GE15158 |
| Mitogen activated protein kinase kinase kinase kinase 2 (predicted) | Map4k2_predicted | CB760256 | 0.53 | GE1170412 |
| Mitogen-activated protein kinase 8 interacting protein 3 | Mapk8ip3 | CB615891 | 1.506 | GE1288677 |
| Mitogen-activated protein kinase kinase kinase 14 (predicted) | Map3k14_predicted | CB585517 | 0.594 | GE1101647 |
| MMR_HSR1 domain containing protein RGD1359460 | RGD1359460 | AI175555 | 1.415 | GE14404 |
| Muscle and microspikes RAS | Mras | AI407001 | 1.665 | GE15048 |
| muscle, skeletal, receptor tyrosine kinase | Nsk1 | NM_031061 | 0.65 | GE20914 |
| myelin-associated oligodendrocytic basic protein | Mobp81; Mobp81p; MGC105491 | NM_012720 | 1.974 | GE21457 |
| myelin-associated oligodendrocytic basic protein | Mobp81; Mobp81p; MGC105491 | NM_012720 | 0.576 | GE1302921 |
| Myeloid/lymphoid or mixed-lineage leukemia (mapped) | Mll_mapped | BI295700 | 1.43 | GE1290378 |
| Myeloid/lymphoid or mixed-lineage leukemia (mapped) | Mll_mapped | CB796575 | 0.604 | GE1244162 |
| Myeloid/lymphoid or mixed-lineage leukemia 5 (trithorax homolog, Drosophila) | Mll5 | AW143757 | 1.454 | GE15907 |
| MYG1 protein | C12orf10 | AA818018 | 1.467 | GE12360 |
| Myomesin 1 (skelemin) 185kDa | Myom1 | BM383962 | 0.65 | GE1270223 |
| myosin, heavy polypeptide 9, non-muscle | Myh9 | NM_013194 | 1.419 | GE20239 |
| Myotrophin | Mtpn | AI170291 | 1.471 | GE14220 |
| NADH dehydrogenase (ubiquinone) 1 alpha subcomplex, 1 (predicted) | Ndufa1_predicted | BF417200 | 1.462 | GE1286706 |
| NADH dehydrogenase (ubiquinone) 1 alpha subcomplex, 2 (predicted) | Ndufa2_predicted | BE114324 | 1.481 | GE1265481 |
| NADH dehydrogenase (ubiquinone) 1 beta subcomplex 3 (predicted) | Ndufb3_predicted | BF417510 | 1.433 | GE1186325 |
| NADH dehydrogenase (ubiquinone) 1 beta subcomplex, 2 (predicted) | Ndufb2_predicted | BM386606 | 1.833 | GE15461 |
| NADH dehydrogenase (ubiquinone) Fe-S protein 3 (predicted) | Ndufs3_predicted | BG379944 | 1.428 | GE1159927 |
| NADH dehydrogenase (ubiquinone) Fe-S protein 8 (predicted) | Ndufs8_predicted | BE118184 | 1.514 | GE1186911 |
| NADH dehydrogenase (ubiquinone) flavoprotein 2 | Ndufv2 | M22756 | 1.566 | GE19677 |
| NCK interacting protein with SH3 domain (predicted) | Nckipsd_predicted | AW916819 | 2.05 | GE16742 |
| nerve growth factor, gamma | KLK1; rGK-1; Klk1c1; preprokallikrein | NM_031523 | 1.443 | GE19663 |
| N-ethylmaleimide sensitive fusion protein attachment protein alpha | Napa | NM_080585 | 1.405 | GE21170 |
| N-ethylmaleimide-sensitive factor attachment protein, gamma | Napg | AA955740 | 1.441 | GE1236212 |
| neurofilament, light polypeptide | Nfl; NF-L | NM_031783 | 1.493 | GE13224 |
| Neurotrophic tyrosine kinase, receptor, type 2 | Ntrk2 | BF388200 | 1.655 | GE1102337 |
| neurturin | Nrtn | NM_053399 | 1.956 | GE1243351 |
| neutrophil cytosolic factor 1 | Ncf-1; p47phox | NM_053734 | 0.674 | GE1145076 |
| NG5 protein | Ng5 | AW524733 | 1.555 | GE16215 |
| NISC_lx11g07.y1 NCI_CGAP_Pr51 Rattus norvegicus cDNA clone IMAGE:5621964 5', mRNA sequence. |  | CA339191 | 0.689 | GE1245880 |
| non-metastatic cells 7, protein expressed in | Nm23-r7 | NM_138532 | 1.421 | GE1248006 |
| Non-POU domain containing, octamer-binding | Nono | CA508538 | 1.468 | GE1253064 |
| Nuclear cap binding protein subunit 1, 80kDa | Ncbp1 | BG375243 | 1.737 | GE14883 |
| Nuclear factor of kappa light chain gene enhancer in B-cells inhibitor, alpha | Nfkbia | CA509173 | 1.461 | GE1265705 |
| Nuclear factor of kappa light polypeptide gene enhancer in B-cells 2, p49/p100 | Nfkb2 | BQ190328 | 1.506 | GE1186357 |
| Nuclear factor, erythroid derived 2,-like 1 (predicted) | Nfe2l1_predicted | BI290000 | 1.43 | GE1151695 |
| Nuclear prelamin A recognition factor | Narf | BM387344 | 1.412 | GE1112994 |
| nuclear protein 1 | p8 | NM_053611 | 0.527 | GE13173 |
| Nuclear receptor coactivator 3 | Ncoa3 | BF552560 | 0.664 | GE1247284 |
| Nuclear receptor interacting protein 3 (predicted) | Nrip3_predicted | BF556360 | 1.454 | GE1111072 |
| nuclear receptor subfamily 4, group A, member 1 | HMR; Nur77; Ngfi-b | NM_024388 | 2.12 | GE1248064 |
| Nucleoporin 188 | Nup188 | AI102685 | 1.786 | GE13925 |
| nucleoporin 88 | Nup84; Prei2 | NM_053616 | 1.423 | GE21071 |
| Nudix (nucleoside diphosphate linked moiety X)-type motif 14 (predicted) | Nudt14_predicted | CA505483 | 1.775 | GE15775 |
| Obscurin, cytoskeletal calmodulin and titin-interacting RhoGEF | Obscn | BF404478 | 1.484 | GE18717 |
| Odd Oz/ten-m homolog 2 (Drosophila) | Odz2 | BF409534 | 0.603 | GE1217075 |
| olfactomedin 1 | Noe1; D2Sut1e | NM_053573 | 1.584 | GE20838 |
| Oligophrenin 1 (predicted) | Ophn1_predicted | BF400678 | 0.708 | GE1140566 |
| Oxysterol binding protein 2 (predicted) | Osbp2_predicted | CB547415 | 1.629 | GE1142664 |
| P55 protein | LOC652956 | AI229529 | 1.578 | GE14687 |
| Pam, highwire, rpm 1 (predicted) | Phr1_predicted | AA800029 | 1.442 | GE12260 |
| PCTAIRE-motif protein kinase 3 | Pctk3 | H32518 | 1.506 | GE1250977 |
| Period homolog 1 (Drosophila) | Per1 | BQ211906 | 1.806 | GE1223779 |
| Period homolog 1 (Drosophila) | Per1 | AA819832 | 1.486 | GE12422 |
| Peroxin 2 | Pex2 | AW528836 | 1.444 | GE1284276 |
| Peroxisomal biogenesis factor 11b | Pex11b | BQ203462 | 1.482 | GE1155436 |
| Peroxisomal delta3, delta2-enoyl-Coenzyme A isomerase | Peci | AW919017 | 1.445 | GE16975 |
| PH domain and leucine rich repeat protein phosphatase | Scop; Plekhe1 | NM_021657 | 0.662 | GE20758 |
| PHD finger protein 2 (predicted) | Phf2_predicted | BI283978 | 1.582 | GE17110 |
| phosphatidic acid phosphatase type 2c | Ppap2c | NM_139252 | 1.617 | GE12690 |
| phosphatidylethanolamine binding protein 1 | Pbp; HCNP; Rkip | NM_017236 | 1.443 | GE22183 |
| phosphatidylinositol transfer protein, alpha | Pitpn | NM_017231 | 1.527 | GE20439 |
| Phosphatidylserine synthase 1 | Ptdss1 | CB780453 | 0.662 | GE1195482 |
| Phosphofructokinase, liver, B-type | Pfkl | BI290618 | 1.58 | GE12403 |
| Phosphoinositide-3-kinase, regulatory subunit 4, p150 (predicted) | Pik3r4_predicted | CB806898 | 0.703 | GE1247893 |
| Phosphorylase kinase, gamma 2 (testis) | Phkg2 | AI072206 | 0.668 | GE1258261 |
| Placental growth factor | Pgf | CK603317 | 0.611 | GE21739 |
| Plasticity related protein 4 | PRG-4 | BM986327 | 1.404 | GE1135352 |
| Pleckstrin homology domain containing, family A member 5 | Plekha5 | BE108016 | 1.681 | GE1302451 |
| Pleckstrin homology domain containing, family A member 5 | Plekha5 | AI009219 | 1.504 | GE13638 |
| Pleckstrin homology domain containing, family F (with FYVE domain) member 2 (predicted) | Plekhf2_predicted | CA511789 | 0.612 | GE1116668 |
| Pleckstrin homology-like domain, family A, member 3 | Phlda3 | AW520812 | 1.813 | GE16169 |
| plectin 1 | Plec1 | NM_022401 | 1.547 | GE21113 |
| Plexin B2 | Plxnb2 | BG377676 | 1.541 | GE1175709 |
| Polycomb group ring finger 2 (predicted) | Pcgf2_predicted | BQ210271 | 1.627 | GE15919 |
| polyglutamine binding protein 1 | Pqbp1 | BC059163 | 1.52 | GE17303 |
| Polymerase (RNA) II (DNA directed) polypeptide F | Polr2f | CB709764 | 0.595 | GE1279463 |
| potassium inwardly-rectifying channel, subfamily J, member 6 | Kcnj6 | NM_013192 | 0.617 | GE20238 |
| potassium voltage gated channel, Shab-related subfamily, member 1 | Shab; Kv2.1; DRK1PC; Kcr1-1 | NM_013186 | 1.499 | GE20233 |
| potassium voltage gated channel, Shab-related subfamily, member 1 | Shab; Kv2.1; DRK1PC; Kcr1-1 | NM_013186 | 1.495 | GE1275453 |
| Potassium voltage-gated channel, shaker-related subfamily, beta member 1 | Kcnab1 | BF396103 | 0.549 | GE1188737 |
| potassium voltage-gated channel, subfamily H (eag-related), member 2 | ERG1 | NM_053949 | 1.73 | GE21227 |
| POU domain, class 3, transcription factor 3 | Pou3f3 | BF386744 | 0.612 | GE1269907 |
| PQ loop repeat containing 1 | Pqlc1 | BQ196010 | 1.462 | GE16490 |
| PRA1 domain family 2 (predicted) | Praf2_predicted | BF545150 | 1.501 | GE14558 |
| Pre-B-cell leukemia transcription factor 1 (predicted) | Pbx1_predicted | AW530825 | 1.501 | GE1228911 |
| Procollagen, type IV, alpha 3 (Goodpasture antigen) binding protein (predicted) | Col4a3bp_predicted | CF115281 | 1.706 | GE1205418 |
| Procollagen, type VI, alpha 1 (predicted) | Col6a1_predicted | AI598402 | 1.506 | GE15511 |
| Procollagen, type XV | Col15a1 | AA800298 | 0.635 | GE12283 |
| Progesterone receptor | Pgr | BE106259 | 1.551 | GE1110148 |
| Progestin and adipoQ receptor family member IV | Paqr4 | CK601392 | 1.508 | GE1291117 |
| Programmed cell death protein 11 (predicted) | Pdcd11_predicted | BM385868 | 1.427 | GE1180325 |
| Progressive ankylosis homolog (mouse) | Ank | BF391129 | 1.78 | GE1260606 |
| Proline-serine-threonine phosphatase-interacting protein 1 (predicted) | Pstpip1_predicted | BQ190642 | 1.481 | GE1125043 |
| Prostate tumor over expressed gene 1 | Ptov1 | H31309 | 1.41 | GE1264520 |
| Protein arginine N-methyltransferase 4 | Prmt4 | CK469490 | 1.994 | GE1218333 |
| Protein disulfide isomerase associated 3 | Pdia3 | CA509557 | 1.456 | GE18656 |
| protein disulfide isomerase associated 3 | ER60; ERp57; Grp58 | NM_017319 | 1.433 | GE20496 |
| protein disulfide isomerase associated 4 | Erp70; Erp72 | NM_053849 | 1.776 | GE1269057 |
| Protein disulfide isomerase associated 4 | Pdia4 | AI237120 | 0.664 | GE1172620 |
| Protein O-linked mannose beta1,2-N-acetylglucosaminyltransferase | Pomgnt1 | AW915001 | 1.987 | GE1138852 |
| protein phosphatase 1, regulatory (inhibitor) subunit 12A | M110; MBSP; Mypt1 | NM_053890 | 1.601 | GE21994 |
| Protein phosphatase 1, regulatory (inhibitor) subunit 16A (predicted) | Ppp1r16a_predicted | BF394856 | 1.424 | GE1162535 |
| Protein phosphatase 2 (formerly 2A), regulatory subunit B (PR 52), beta isoform | Ppp2r2b | AW529564 | 1.669 | GE1144404 |
| Protein phosphatase 3, catalytic subunit, alpha isoform | Ppp3ca | BQ210125 | 1.455 | GE1296551 |
| protein phosphatase 6, catalytic subunit | MGC93213 | NM_133589 | 1.893 | GE21147 |
| Protein regulator of cytokinesis 1 (predicted) | Prc1_predicted | AI113104 | 0.53 | GE14064 |
| Protein tyrosine phosphatase 4a3 (predicted) | Ptp4a3_predicted | BE112125 | 1.523 | GE14016 |
| Protein tyrosine phosphatase, non-receptor type 3 | Ptpn3 | AI555271 | 1.54 | GE1211180 |
| Protocadherin gamma subfamily C, 3 | Pcdhgc3 | CB796773 | 0.603 | GE1237378 |
| PRP19/PSO4 pre-mRNA processing factor 19 homolog (S. cerevisiae) | Prp19; MGC124562 | NM_139333 | 1.543 | GE1147016 |
| Pseudouridylate synthase 7 homolog (S. cerevisiae) (predicted) | Pus7_predicted | BM390168 | 2.337 | GE1172168 |
| putative pheromone receptor (Go-VN5) | LOC286914 | NM_173130 | 0.615 | GE13181 |
| putative pheromone receptor VN6 | LOC266771; V1ra16 | NM_153729 | 1.407 | GE1187051 |
| pyruvate dehydrogenase kinase, isoenzyme 1 | MGC108625 | NM_053826 | 1.475 | GE19639 |
| Pyruvate kinase, muscle | Pkm2 | CK358492 | 0.647 | GE1278525 |
| queuine tRNA-ribosyltransferase 1 | Tgt; Tgut | NM_022250 | 1.457 | GE1187376 |
| Quinolinate phosphoribosyltransferase | Qprt | AI407095 | 0.659 | GE15055 |
| RAB14, member RAS oncogene family | Rab14 | CA509420 | 1.559 | GE1243491 |
| RAB15, member RAS onocogene family | Rab15 | BQ194530 | 1.446 | GE1184287 |
| RAB31, member RAS oncogene family | Rab0 | NM_145094 | 1.712 | GE13516 |
| RAB6B, member RAS oncogene family (predicted) | Rab6b_predicted | BF283901 | 1.494 | GE18214 |
| RAB6B, member RAS oncogene family (predicted) | Rab6b_predicted | BG380450 | 1.404 | GE1276879 |
| RAN binding protein 5 (predicted) | Ranbp5_predicted | BQ202763 | 1.407 | GE1112133 |
| Rap guanine nucleotide exchange factor (GEF) 4 | Cgef2; Epac2; CAMP-GEFII | U78517 | 1.459 | GE22054 |
| Ras homolog gene family, member C (predicted) | Rhoc_predicted | AA891940 | 1.619 | GE12629 |
| Ras homolog gene family, member G | Rhog | BE118414 | 1.574 | GE17940 |
| ras homolog gene family, member T2 | Miro2 | NM_181823 | 1.419 | GE1120654 |
| RAS protein-specific guanine nucleotide-releasing factor 1 | Rasgrf1 | BF563233 | 1.577 | GE1152978 |
| RAS-like, family 2, locus 9 | Rasl2-9; GTP-ase Ran | AF507943 | 2.019 | GE1128152 |
| Ras-related C3 botulinum toxin substrate 1 | Rac1 | BG664778 | 1.477 | GE13970 |
| RecQ protein-like | Recql | AA891612 | 1.506 | GE15618 |
| Regulator of chromosome condensation 2 (predicted) | Rcc2_predicted | AI236185 | 1.568 | GE1285866 |
| Regulator of G-protein signaling 17 (predicted) | Rgs17_predicted | AW140991 | 1.561 | GE15752 |
| regulator of G-protein signaling 4 | Rgs4 | NM_017214 | 1.551 | GE1196053 |
| regulator of G-protein signaling 9 | Rgs9 | NM_019224 | 0.678 | GE20597 |
| retinal S-antigen | SAGMR; SANTI | NM_013023 | 0.655 | GE20123 |
| Retinoic acid induced 17 (predicted) | Rai17_predicted | BF553300 | 1.417 | GE1219966 |
| Retinol dehydrogenase 10 (all-trans) | Rdh10 | BE096510 | 1.547 | GE1178569 |
| RGD1564379 (predicted) | RGD1564379_predicted | BF284939 | 1.521 | GE18280 |
| Rho guanine nucleotide exchange factor (GEF) 1 | Lsc; MGC108950 | AJ236911 | 1.633 | GE20778 |
| Rho-associated coiled-coil forming kinase 2 | ROK | NM_013022 | 1.773 | GE20122 |
| Rhotekin | Rtkn | CB324632 | 0.69 | GE1294954 |
| Ribose-phosphate pyrophosphokinase I -like | LOC314140 | BG374028 | 1.664 | GE1124932 |
| ribosomal protein L10 | Rpl10 | NM_031100 | 1.516 | GE21165 |
| ribosomal protein L19 | Rpl19 | NM_031103 | 1.419 | GE1140362 |
| Ribosomal protein L27a (predicted) | Rpl27a_predicted | AA900726 | 1.455 | GE1203933 |
| Ribosomal protein L32 | Rpl32 | AA799875 | 1.864 | GE20255 |
| Ribosomal protein L6 | Rpl6 | CB581089 | 0.626 | GE1186818 |
| ribosomal protein S10 | Rps10 | NM_031109 | 1.551 | GE21090 |
| Ribosomal protein S16 | Rps16 | BI273793 | 2.087 | GE1163435 |
| Ribosome binding protein 1 homolog 180kDa (dog) (predicted) | Rrbp1_predicted | AA848501 | 1.407 | GE12437 |
| Ring finger protein 1 | Ring1 | AA944239 | 1.972 | GE1305362 |
| Ring finger protein 187 (predicted) | Rnf187_predicted | AW144084 | 1.65 | GE15932 |
| Ring finger protein 32 | Rnf32 | BF563237 | 1.43 | GE1258603 |
| RNA (guanine-9-) methyltransferase domain containing 1 | Rg9mtd1 | BF554013 | 1.461 | GE1146669 |
| RNA binding motif protein 14 | Rbm14 | AW918441 | 1.603 | GE16910 |
| RNA binding motif protein 24 (predicted) | Rbm24_predicted | AA899570 | 1.452 | GE1100788 |
| RNA-binding protein 12 | Rbm12 | BI290911 | 1.545 | GE1185152 |
| RNA-binding region (RNP1, RRM) containing 1 (predicted) | Rnpc1_predicted | CA506740 | 1.66 | GE1200748 |
| Rous sarcoma oncogene | Src | AF130457 | 1.434 | GE21437 |
| RT1 class Ib, locus Aw2 | RT1-Aw2 | BI275216 | 1.908 | GE1280096 |
| RT1 class II, locus Da | RT1-u; RT1-Daa; RT1-Dab; RT1-Dac; RT1-Dad; RT1-Daf; RT1-Dah; RT1-Dak; RT1-Dal; RT1-Dam; RT1-Dan; MGC112637 | Y00480 | 0.643 | GE21186 |
| runt related transcription factor 1 | Aml1; Cbfa2 | NM_017325 | 1.565 | GE20500 |
| Ryanodine receptor 2, cardiac | Ryr2 | BF549584 | 1.462 | GE1220179 |
| Ryanodine receptor 3 | Ryr3 | AW529195 | 0.672 | GE1227628 |
| SAR1 gene homolog A (S. cerevisiae) | Sar1a | CD371288 | 1.665 | GE1251018 |
| SCF apoptosis response protein 1 | LOC499941 | AI175520 | 1.424 | GE15051 |
| SCY1-like 1 (S. cerevisiae) | Scyl1 | CK474641 | 1.736 | GE1248551 |
| SEC23A (S. cerevisiae) (predicted) | Sec23a_predicted | CA504907 | 1.402 | GE1201417 |
| Sec61 beta subunit (predicted) | Sec61b_predicted | CA507594 | 1.446 | GE1269047 |
| Secreted Ly6/Plaur domain containing 1 (predicted) | Slurp1_predicted | BG371786 | 0.577 | GE13826 |
| selenoprotein S | Sels; Ratsg2 | NM_173120 | 1.405 | GE15419 |
| septin 2 | Nedd5; Vesp11; MGC93254 | NM_057148 | 1.433 | GE1148216 |
| Ser/Thr-like protein kinase lyk4 | Lyk4 | BF420075 | 1.704 | GE1157247 |
| serine/threonine-protein kinase pim-3 | Pim3 | NM_022602 | 1.506 | GE1167704 |
| SERTA domain containing 1 | MGC72577 | BC061808 | 1.583 | GE12856 |
| serum/glucocorticoid regulated kinase | Sgk | NM_019232 | 1.447 | GE1305161 |
| SH2 domain containing 3C (predicted) | Sh2d3c_predicted | BQ205034 | 1.568 | GE18716 |
| SH3-domain GRB2-like 1 | SH3P8 | NM_031239 | 1.511 | GE1170336 |
| Shultzomica06099 Rat lung airway and parenchyma cDNA libraries Rattus norvegicus cDNA clone CP2368 5', mRNA sequence. |  | CF112848 | 0.652 | GE1195236 |
| Signal-induced proliferation-associated 1 like 1 | Sipa1l1 | BE109893 | 1.471 | GE1284364 |
| Signal-induced proliferation-associated 1 like 1 | Sipa1l1 | BF416623 | 0.547 | GE1169078 |
| Silver homolog (mouse) (predicted) | Silv_predicted | BI300443 | 0.651 | GE1220311 |
| Similar to 106 kDa O-GlcNAc transferase-interacting protein (predicted) | RGD1307844_predicted | BE118465 | 0.713 | GE17945 |
| Similar to 1700123O20Rik protein (predicted) | RGD1308430_predicted | BQ207968 | 0.6 | GE1277747 |
| Similar to 2310002F18Rik protein | MGC124824 | AJ225647 | 1.555 | GE15712 |
| Similar to 2410024A21Rik protein | RGD1304878 | BI293637 | 1.519 | GE1262027 |
| Similar to 4921510J17Rik protein | RGD1305423 | CB788639 | 1.495 | GE1118843 |
| similar to 6030490I01Rik | MGC72997 | BC061577 | 1.682 | GE1297321 |
| Similar to A disintegrin-like and metalloprotease (reprolysin type) with thrombospondin type 1 motif, 2 (predicted) | RGD1565950_predicted | BF563201 | 1.452 | GE19382 |
| Similar to adenosine monophosphate deaminase 2 (isoform L) | LOC362015 | BG372402 | 1.438 | GE17694 |
| Similar to alcohol dehydrogenase PAN2 (predicted) | RGD1565196_predicted | AA997435 | 1.436 | GE12983 |
| Similar to ankyrin repeat and SOCS box-containing protein 14 | LOC680076 | AI136492 | 1.444 | GE1295107 |
| Similar to AP2 associated kinase 1 (predicted) | RGD1563580_predicted | AW533060 | 1.453 | GE16367 |
| Similar to AP2 associated kinase 1 (predicted) | RGD1563580_predicted | CB609394 | 0.533 | GE1122163 |
| Similar to Autophagy-specific gene 2 CG1241-PA | LOC689688 | BF557395 | 1.442 | GE19318 |
| Similar to BC002216 protein | MGC94339 | AW144294 | 1.618 | GE15940 |
| Similar to boule (predicted) | RGD1559527_predicted | AW142998 | 0.686 | GE1122864 |
| Similar to Brain protein 44 (predicted) | RGD1563422_predicted | BE112736 | 1.686 | GE1204284 |
| Similar to calmodulin regulated spectrin-associated protein 1 (predicted) | RGD1565022_predicted | BE107223 | 1.602 | GE17392 |
| Similar to cDNA sequence AF096286; pecanex 1 (predicted) | RGD1305883_predicted | BF523626 | 1.455 | GE1244034 |
| Similar to cDNA sequence BC004044 | RGD1311980 | AI178361 | 1.59 | GE14545 |
| Similar to cDNA sequence BC024814 (predicted) | RGD1311433_predicted | BQ196125 | 1.405 | GE1103006 |
| Similar to cDNA sequence BC032204 (predicted) | RGD1310168_predicted | CB749429 | 1.891 | GE1246986 |
| Similar to CG10084-PA | RGD1308297 | AA901298 | 1.406 | GE1175518 |
| Similar to CG11388-PA (predicted) | RGD1308154_predicted | BI284360 | 1.409 | GE14432 |
| Similar to CG18661-PA | RGD1307155 | BQ205817 | 1.438 | GE13604 |
| Similar to CGI-112 protein | RGD1308113 | BG381305 | 1.479 | GE1145175 |
| Similar to CGTHBA protein (-14 gene protein) | RGD1308665 | BF558866 | 1.56 | GE19355 |
| Similar to chromosome 9 open reading frame 7 (predicted) | RGD1311501_predicted | BM389779 | 1.475 | GE14977 |
| Similar to CLIP-170-related protein (predicted) | RGD1306245_predicted | BF550922 | 1.507 | GE1190579 |
| Similar to Coiled-coil domain containing protein 3 precursor (predicted) | RGD1559473_predicted | CB605587 | 1.415 | GE1126620 |
| Similar to dehydrogenase/reductase (SDR family) member 10 | LOC691018 | BF543501 | 0.559 | GE1143489 |
| Similar to Delta-interacting protein A (Hepatitis delta antigen interacting protein A) (predicted) | RGD1565319_predicted | BQ205281 | 1.408 | GE1206638 |
| Similar to Deltex3 (predicted) | RGD1566181_predicted | BI298235 | 1.629 | GE1269550 |
| Similar to DNA segment, Chr 4, ERATO Doi 22, expressed (predicted) | RGD1560286_predicted | BQ206818 | 1.964 | GE1177005 |
| Similar to DnaJ (Hsp40) homolog, subfamily B, member 12 | LOC294513 | CK599186 | 1.583 | GE1169962 |
| Similar to DnaJ (Hsp40) homolog, subfamily B, member 12 | LOC294513 | BF393399 | 1.539 | GE12398 |
| Similar to Dynamin-binding protein (Scaffold protein Tuba) | LOC309362 | CA506248 | 1.5 | GE1280912 |
| Similar to E430002G05Rik protein (predicted) | RGD1308745_predicted | BQ205135 | 0.634 | GE1210573 |
| Similar to Endoplasmic reticulum mannosyl-oligosaccharide 1,2-alpha-mannosidase (ER alpha-1,2-mannosidase) (predicted) | RGD1563595_predicted | BM385415 | 1.885 | GE17424 |
| Similar to ENSANGP00000021391 (predicted) | RGD1309779_predicted | BF550632 | 1.468 | GE1100511 |
| Similar to eukaryotic translation initiation factor 4A, isoform 1 | LOC500654 | BI280182 | 1.614 | GE1211683 |
| Similar to expressed sequence AA408877 | RGD1308513 | BF398568 | 0.529 | GE1128322 |
| Similar to expressed sequence AI317237 (predicted) | RGD1305671_predicted | AI555860 | 1.541 | GE1199326 |
| Similar to expressed sequence AI836003 | MGC114464 | BF400266 | 1.555 | GE1168583 |
| Similar to expressed sequence AW556797 (predicted) | RGD1305138_predicted | H32843 | 1.422 | GE14809 |
| Similar to family with sequence similarity 40, member A | LOC362012 | AI232979 | 1.414 | GE14820 |
| Similar to F-box and leucine-rich repeat protein 18 | LOC678726 | CB615595 | 1.985 | GE1101975 |
| Similar to Flt3 interacting zinc finger protein 1 (predicted) | RGD1306359_predicted | H34394 | 1.458 | GE1135388 |
| Similar to Fus1 protein | LOC501052 | AW916256 | 1.457 | GE1201012 |
| Similar to G protein-coupled receptor GPR75 (predicted) | RGD1566385_predicted | CB605999 | 1.483 | GE1177800 |
| Similar to golgi-specific brefeldin A-resistance guanine nucleotide exchange factor 1 (predicted) | RGD1307160_predicted | CA507087 | 1.468 | GE1234461 |
| Similar to HCV NS3-transactivated protein 1 (predicted) | RGD1306332_predicted | CA504560 | 1.508 | GE1239667 |
| Similar to HSPC288 (predicted) | RGD1310769_predicted | AI763549 | 1.533 | GE1187280 |
| Similar to hypothetical protein | LOC361041 | BF542426 | 1.69 | GE19118 |
| Similar to hypothetical protein | LOC361041 | BI274280 | 1.618 | GE1217431 |
| Similar to Hypothetical protein 6330514E13 (predicted) | RGD1559693_predicted | BE097514 | 1.417 | GE17371 |
| Similar to hypothetical protein A430031N04 (predicted) | RGD1559643_predicted | BE113205 | 1.426 | GE17779 |
| Similar to hypothetical protein A530094D01 (predicted) | RGD1306556_predicted | BF393118 | 0.483 | GE1152484 |
| Similar to hypothetical protein B230397C21 (predicted) | RGD1564725_predicted | BI296117 | 1.436 | GE1187970 |
| Similar to hypothetical protein B930074I24 (predicted) | RGD1560774_predicted | AI548280 | 0.598 | GE1112607 |
| Similar to hypothetical protein D15Ertd785e | MGC114417 | AA955172 | 1.413 | GE12926 |
| Similar to hypothetical protein FLJ11526 (predicted) | RGD1309585_predicted | BF551390 | 1.634 | GE1118411 |
| Similar to hypothetical protein FLJ14800 (predicted) | RGD1561500_predicted | BI276204 | 1.463 | GE1118058 |
| Similar to hypothetical protein FLJ21616 (predicted) | RGD1306787_predicted | AI501096 | 0.619 | GE1151420 |
| Similar to hypothetical protein FLJ22625 | RGD1304696 | AA849788 | 1.462 | GE12473 |
| Similar to hypothetical protein FLJ23518 | MGC94183 | BQ203264 | 1.439 | GE1264806 |
| Similar to hypothetical protein FLJ30596 (predicted) | RGD1306809_predicted | CK598541 | 1.532 | GE1192017 |
| Similar to hypothetical protein FLJ34389 (predicted) | RGD1305243_predicted | BF521643 | 0.534 | GE1179329 |
| Similar to Hypothetical protein LOC73072 (predicted) | RGD1566084_predicted | BE115939 | 1.486 | GE16334 |
| Similar to hypothetical protein MGC20983 (predicted) | RGD1310048_predicted | AI144607 | 0.647 | GE1166513 |
| Similar to hypothetical protein MGC33486 (predicted) | RGD1310680_predicted | AI716115 | 1.667 | GE15640 |
| Similar to hypothetical protein MGC3731 | RGD1305327 | BQ206394 | 1.616 | GE15053 |
| Similar to KCCR13L (predicted) | RGD1310193_predicted | BQ207242 | 1.522 | GE1252691 |
| Similar to Kelch-like protein 3 (predicted) | RGD1565218_predicted | BF416150 | 1.476 | GE18983 |
| Similar to Kell protein (predicted) | RGD1565470_predicted | BF420752 | 0.701 | GE14594 |
| Similar to KIAA1193 protein (predicted) | RGD1307278_predicted | BU759570 | 1.476 | GE1162267 |
| Similar to KIAA1205 protein (predicted) | RGD1309896_predicted | CA506176 | 1.625 | GE1128373 |
| Similar to KIAA1549 protein (predicted) | RGD1306271_predicted | AW530961 | 1.512 | GE1292139 |
| Similar to KIAA1900 protein (predicted) | RGD1310364_predicted | BE096676 | 1.449 | GE1233722 |
| Similar to KIAA1960 protein (predicted) | RGD1308066_predicted | BF393390 | 3.812 | GE1136324 |
| Similar to lactation elevated 1 | LOC502479 | AW921797 | 1.506 | GE17088 |
| Similar to ligatin | LOC498225 | AA799302 | 1.557 | GE1240436 |
| Similar to lipoma HMGIC fusion partner-like 3 (predicted) | RGD1559727_predicted | CB607423 | 0.703 | GE1177763 |
| Similar to methionine sulfoxide reductase B3 isoform 2 | LOC680036 | AI575248 | 1.442 | GE1152564 |
| Similar to MGC15476 protein (predicted) | RGD1563892_predicted | BE112850 | 0.619 | GE1221317 |
| Similar to Mitochondrial 28S ribosomal protein S28 (S28mt) (MRP-S28) | LOC689025 | BQ191819 | 1.646 | GE17133 |
| Similar to mKIAA0518 protein (predicted) | RGD1561597_predicted | BU760111 | 1.531 | GE19346 |
| Similar to mKIAA1002 protein | RGD1310066 | AI176739 | 1.441 | GE14471 |
| Similar to mKIAA1208 protein (predicted) | RGD1564821_predicted | CB576757 | 0.699 | GE1286444 |
| Similar to mKIAA1238 protein (predicted) | RGD1560851_predicted | AI177186 | 1.484 | GE14509 |
| Similar to Msx2-interacting protein (SPEN homolog) (SMART/HDAC1-associated repressor protein) | LOC690911 | BI295169 | 1.574 | GE1281798 |
| Similar to myocyte enhancer factor 2C | LOC309957 | AI231450 | 2.112 | GE14761 |
| Similar to myosin heavy chain Myr 8 | LOC680480 | AW915635 | 2.032 | GE1108210 |
| Similar to N-acetylated alpha-linked acidic dipeptidase 2 (predicted) | RGD1565147_predicted | AW527741 | 1.422 | GE1283613 |
| Similar to neuron navigator 1 | LOC685707 | BE100576 | 1.414 | GE17216 |
| Similar to novel protein (predicted) | RGD1560608_predicted | BF410717 | 0.673 | GE19049 |
| Similar to novel protein similar to multidomain presynaptic cytomatrix protein piccolo (presynaptic cytomatrix protein) (predicted) | RGD1564117_predicted | CB735839 | 1.755 | GE1123863 |
| Similar to novel protein similar to Tensin Tns (predicted) | RGD1564174_predicted | AI145203 | 0.577 | GE1231715 |
| Similar to NYD-SP28 protein | LOC362994 | BQ194689 | 1.538 | GE1104121 |
| Similar to opposite strand transcription unit to Stag3; Gats protein | RGD1304774 | BU760287 | 1.992 | GE15471 |
| Similar to ornithine decarboxylase-like protein | LOC366473 | BE121288 | 1.412 | GE1201352 |
| Similar to pancreatitis-induced protein 49 | RGD1308600 | BG377380 | 1.517 | GE1121855 |
| Similar to PD-1-ligand precursor (predicted) | RGD1566211_predicted | AW521319 | 2.212 | GE1109217 |
| Similar to Phosphatidylserine decarboxylase proenzyme | LOC681361 | AW529876 | 0.567 | GE1180632 |
| Similar to Protein C22orf5 | RGD1306591 | AA955564 | 1.414 | GE17123 |
| Similar to protein phosphatase 1, regulatory subunit 12C | LOC499076 | BF390978 | 1.535 | GE1205079 |
| Similar to RAD54B homolog isoform 1; RAD54, S. cerevisiae, homolog of, B (predicted) | RGD1306507_predicted | BF290076 | 1.822 | GE18389 |
| Similar to RAS-like, estrogen-regulated, growth-inhibitor (predicted) | RGD1562829_predicted | AI059184 | 0.488 | GE14331 |
| Similar to reprimo, TP53 dependent G2 arrest mediator candidate | LOC680110 | CK481640 | 1.448 | GE1155283 |
| Similar to Rho GTPase activating protein 10 | LOC688429 | AI408928 | 1.768 | GE15135 |
| Similar to Rho guanine nucleotide exchange factor 4 isoform a | LOC301334 | CB733647 | 1.606 | GE1222311 |
| Similar to ribosomal protein L10 (predicted) | RGD1564963_predicted | AI705544 | 1.49 | GE1181927 |
| Similar to ribosomal protein L7a | LOC366564 | AI576353 | 0.662 | GE1174266 |
| Similar to RIKEN cDNA 1300010M03 | RGD1306844 | BF388424 | 1.489 | GE1152498 |
| Similar to RIKEN cDNA 1500009M05 (predicted) | RGD1566242_predicted | BQ196258 | 1.409 | GE1172415 |
| Similar to RIKEN cDNA 1500031M22 (predicted) | RGD1311752_predicted | AI043960 | 0.605 | GE1268261 |
| Similar to RIKEN cDNA 1700012G19 gene (predicted) | RGD1307773_predicted | BF420654 | 1.465 | GE19075 |
| Similar to RIKEN cDNA 1700012G19 gene (predicted) | RGD1307773_predicted | BF389521 | 1.464 | GE16807 |
| Similar to RIKEN cDNA 2310008M10 (predicted) | RGD1560708_predicted | BI298029 | 1.414 | GE15567 |
| Similar to RIKEN cDNA 2310035C23 (predicted) | RGD1307235_predicted | CK474870 | 1.433 | GE1238706 |
| Similar to RIKEN cDNA 2310045A20 (predicted) | RGD1562860_predicted | BQ202344 | 1.485 | GE1241303 |
| Similar to RIKEN cDNA 2410025L10 (predicted) | RGD1563342_predicted | BF414010 | 1.579 | GE18944 |
| Similar to RIKEN cDNA 2810409H07 | RGD1307982 | H33539 | 1.408 | GE1246636 |
| Similar to RIKEN cDNA 2810453I06 | LOC498145 | BF389498 | 1.506 | GE1116747 |
| Similar to RIKEN cDNA 3100004P22 | RGD1306184 | H33621 | 1.563 | GE1156484 |
| Similar to RIKEN cDNA 4631403P03 | RGD1305276 | BI287028 | 1.482 | GE1176085 |
| Similar to RIKEN cDNA 4930524B15 (predicted) | RGD1311343_predicted | BG379269 | 0.709 | GE1112253 |
| Similar to RIKEN cDNA 4930570C03 | LOC300191 | CB547595 | 1.431 | GE1301191 |
| Similar to RIKEN cDNA 4932432K03 | RGD1308087 | AW918111 | 0.646 | GE1119755 |
| Similar to RIKEN cDNA 4933406L09 | LOC361016 | BF412190 | 0.623 | GE1170203 |
| Similar to RIKEN cDNA 5133401N09 | MGC125086 | AW915843 | 1.722 | GE16658 |
| Similar to RIKEN cDNA 5230400G24 | RGD1310230 | Z83048 | 1.453 | GE1222279 |
| Similar to RIKEN cDNA 5730470L24 | RGD1311316 | BE102137 | 1.699 | GE1246784 |
| Similar to RIKEN cDNA 5830433M19 | MGC125002 | BF287691 | 1.63 | GE1110831 |
| Similar to RIKEN cDNA 6330406I15 (predicted) | RGD1307396_predicted | BI297908 | 1.445 | GE18118 |
| Similar to RIKEN cDNA A530089I17 (predicted) | RGD1311375_predicted | BQ211080 | 1.464 | GE1255258 |
| Similar to RIKEN cDNA C130022K22 gene | RGD1305225 | AW919046 | 1.587 | GE16978 |
| Similar to RIKEN cDNA C530028O21 gene | RGD1304952 | C06982 | 1.704 | GE19411 |
| Similar to RIKEN cDNA C730048E16 | RGD1309552 | BI278594 | 1.585 | GE1255606 |
| Similar to RIKEN cDNA E130201N16 (predicted) | RGD1311589_predicted | BE113624 | 1.507 | GE17837 |
| Similar to RNA binding protein gene with multiple splicing (predicted) | RGD1561067_predicted | AI231834 | 0.674 | GE14788 |
| Similar to RNA binding protein with multiple splicing 2 (predicted) | RGD1561222_predicted | BM389543 | 0.69 | GE14464 |
| Similar to RRP22 (predicted) | RGD1306100_predicted | H33847 | 1.435 | GE15376 |
| Similar to RUN and FYVE domain-containing 2 | LOC690777 | BF409097 | 1.816 | GE18734 |
| Similar to scaffold attachment factor B2 | LOC301126 | CB750174 | 1.887 | GE1206284 |
| Similar to SEC14 and spectrin domains 1 (predicted) | RGD1562244_predicted | AA946394 | 1.686 | GE12909 |
| Similar to SET binding factor 2 | LOC691036 | BF396622 | 0.651 | GE1269740 |
| Similar to SH3 binding domain protein 5 like | LOC690898 | BF523438 | 1.795 | GE1134201 |
| Similar to SLIT-ROBO Rho GTPase-activating protein 1 (predicted) | RGD1566260_predicted | AI113165 | 0.667 | GE1272962 |
| Similar to solute carrier family 25, member 28 | LOC688811 | CK222388 | 1.511 | GE16549 |
| Similar to Sorting nexin 6 (TRAF4-associated factor 2) (predicted) | RGD1560591_predicted | CB713107 | 1.485 | GE1288495 |
| Similar to sperm antigen HCMOGT-1 | RGD1309718 | CA504602 | 1.515 | GE1229804 |
| Similar to src homology 2 domain-containing transforming protein D | LOC316507 | BE117304 | 1.48 | GE1302039 |
| Similar to SWI/SNF-related matrix-associated actin-dependent regulator of chromatin c2 | LOC685179 | BF396079 | 1.743 | GE18513 |
| Similar to TAFA5 (predicted) | RGD1562115_predicted | BF557676 | 1.433 | GE1244255 |
| Similar to thrombospondin, type I, domain containing 2 (predicted) | RGD1563246_predicted | BG377729 | 1.429 | GE1187341 |
| Similar to translin-associated factor X (Tsnax) interacting protein 1 (predicted) | RGD1565341_predicted | BF400842 | 1.725 | GE1112751 |
| Similar to ubiquitin-conjugating enzyme E2 variant 1 | LOC679539 | AW142658 | 1.489 | GE1250895 |
| Similar to Vacuolar protein sorting 26 homolog (VPS26 protein homolog) | LOC300472 | BQ194401 | 1.445 | GE1118470 |
| Similar to YY1-associated factor 2 | LOC690262 | BG671856 | 1.414 | GE13730 |
| Similar to zinc finger protein 458 | LOC365723 | BQ781825 | 2.462 | GE1294374 |
| Similar to zinc finger protein 652 (predicted) | RGD1566329_predicted | AW921067 | 0.508 | GE1272456 |
| Similar to zinc finger protein 740 | LOC685834 | CB784917 | 0.699 | GE1243682 |
| Similar to zinc finger, BED domain containing 4 | LOC688556 | BF389833 | 1.582 | GE14219 |
| Sjogren syndrome antigen B | Ssb | AW141580 | 1.57 | GE21128 |
| small cell adhesion glycoprotein | MGC124543 | NM_182817 | 1.483 | GE15101 |
| Small nuclear RNA activating complex, polypeptide 2 | Snapc2 | BG375120 | 1.403 | GE14496 |
| Smoothelin | Smtn | AI547603 | 1.609 | GE16141 |
| SNAP25-interacting protein | Snip | BF404355 | 1.445 | GE1206582 |
| SNAP25-interacting protein | Snip | BE120207 | 1.435 | GE1146736 |
| sodium channel, voltage-gated, type 10, alpha polypeptide | PN3; Na(V)1.8 | NM_017247 | 1.452 | GE20447 |
| Solute carrier family 1 (glutamate/neutral amino acid transporter), member 4 | Slc1a4 | BF413057 | 1.407 | GE1239131 |
| Solute carrier family 14 (urea transporter), member 2 | Slc14a2 | AF230638 | 0.574 | GE13494 |
| Solute carrier family 20, member 2 | Slc20a2 | AI409899 | 1.447 | GE15183 |
| solute carrier family 23 (nucleobase transporters), member 1 | SVCT1 | NM_017315 | 0.676 | GE20493 |
| Solute carrier family 26, member 11 (predicted) | Slc26a11_predicted | BF393869 | 1.455 | GE1263971 |
| solute carrier family 26, member 4 | Pds | NM_019214 | 0.655 | GE20589 |
| solute carrier family 35, member E4 | MGC93128 | NM_153316 | 1.491 | GE13436 |
| Solute carrier family 39 (metal ion transporter), member 11 | Slc39a11 | BF553125 | 0.655 | GE1236645 |
| solute carrier family 4, member 3 | Ae3; Aep3 | NM_017049 | 1.451 | GE20303 |
| Solute carrier family 4, sodium bicarbonate transporter-like, member 11 (predicted) | Slc4a11_predicted | AI233752 | 1.449 | GE14848 |
| Solute carrier family 5 (inositol transporters), member 3 | Slc5a3 | AW251238 | 1.542 | GE15990 |
| somatostatin receptor 1 | Gpcrrna | NM_012719 | 1.476 | GE19921 |
| somatostatin receptor 2 | Smstr2 | NM_019348 | 1.506 | GE21868 |
| Son cell proliferation protein | Son | BE107234 | 1.511 | GE17393 |
| Son cell proliferation protein | Son | AW144637 | 1.486 | GE15970 |
| Sortilin-related receptor, L(DLR class) A repeats-containing (predicted) | Sorl1_predicted | BQ208287 | 1.567 | GE1135618 |
| Sortilin-related VPS10 domain containing receptor 2 (predicted) | Sorcs2_predicted | CB696424 | 1.46 | GE1119571 |
| Sortilin-related VPS10 domain containing receptor 2 (predicted) | Sorcs2_predicted | BF403840 | 0.458 | GE1156497 |
| Sp1 transcription factor | Sp1 | CA508480 | 1.518 | GE1223041 |
| SPARC-like 1 (mast9, hevin) | Sc1; Ecm2 | NM_012946 | 1.453 | GE20075 |
| Spastin (predicted) | Spast_predicted | AI715704 | 1.483 | GE1158943 |
| spectrin beta 3 | Sptbn2 | NM_019167 | 1.455 | GE20563 |
| sperm associated antigen 4 | Spag4 | NM_031792 | 1.488 | GE13247 |
| sperm protein 3111 | Sp3111 | NM_181082 | 0.671 | GE1159986 |
| Sprouty protein with EVH-1 domain 1, related sequence | Spred1 | AI409040 | 1.542 | GE15147 |
| squamous cell carcinoma antigen recognized by T-cells 1 | Sart1 | NM_031596 | 1.421 | GE13050 |
| SREBP cleavage activating protein (predicted) | Scap_predicted | R46972 | 1.541 | GE1276817 |
| Striatin, calmodulin binding protein 4 (predicted) | Strn4_predicted | CK474308 | 1.434 | GE1237739 |
| Striatin, calmodulin binding protein 4 (predicted) | Strn4_predicted | CA505964 | 1.414 | GE15467 |
| Suppressor of cytokine signaling 6 (predicted) | Socs6_predicted | CA507026 | 1.436 | GE1255407 |
| Suppressor of Ty 5 homolog (S. cerevisiae) | Supt5h | CB785406 | 1.513 | GE1164050 |
| synapsin I | Syn1 | NM_019133 | 1.691 | GE20537 |
| synapsin I | Syn1 | NM_019133 | 1.655 | GE22090 |
| Synaptobrevin-like 1 | Sybl1 | AB054998 | 1.638 | GE1208828 |
| Synaptosomal-associated protein, 91kDa homolog (mouse) | Snap91 | BE114404 | 1.468 | GE1230101 |
| Synaptotagmin I | Syt1 | AI575646 | 1.483 | GE1154650 |
| synaptotagmin V | SytIX; MGC105442 | NM_019350 | 1.591 | GE20676 |
| synonyms: iPla2, MGC93880; phospholipase A2, group VI (cytosolic, calcium-independent); Rattus norvegicus phospholipase A2, group VI (Pla2g6), mRNA. | Pla2g6 | NM_053892 | 1.435 | GE1249871 |
| Syntaxin binding protein 1 | Stxbp1 | BF390596 | 1.443 | GE1237249 |
| Synuclein, beta | Sncb | BE096373 | 1.447 | GE17120 |
| Tankyrase, TRF1-interacting ankyrin-related ADP-ribose polymerase 2 (predicted) | Tnks2_predicted | CB751499 | 0.676 | GE1162996 |
| TAR (HIV) RNA binding protein 2 | Tarbp2 | BE114132 | 1.513 | GE17691 |
| taste receptor, type 2, member 7 | T2R7 | NM_023997 | 0.538 | GE1174100 |
| Tax1 (human T-cell leukemia virus type I) binding protein 3 | Tax1bp3 | AW144510 | 1.561 | GE15966 |
| TBC1 domain family, member 1 (predicted) | Tbc1d1_predicted | BF405886 | 1.768 | GE1292184 |
| TBC1 domain family, member 14 | Tbc1d14 | BG374930 | 1.487 | GE15337 |
| TBC1 domain family, member 20 | Tbc1d20 | BE116161 | 1.493 | GE1152795 |
| TBC1 domain family, member 20 | Tbc1d20 | BE118229 | 1.437 | GE1272389 |
| TEA domain family member 1 | Tead1; TEF-1 | AY529195 | 2.065 | GE1195198 |
| Testis expressed gene 10 (predicted) | Tex10_predicted | BE098401 | 1.436 | GE1192734 |
| testis specific protein kinase 1 | Tesk1 | NM_031578 | 1.421 | GE19507 |
| Testis-specific protein, Y-encoded-like | Tspyl | BM391076 | 0.622 | GE1256012 |
| Tetraspanin 18 (predicted) | Tspan18_predicted | BE114106 | 0.633 | GE1165177 |
| thimet oligopeptidase 1 | EP24.15; MGC93105 | NM_172075 | 1.61 | GE19716 |
| Thioredoxin 2 | Txn2 | BI288617 | 0.655 | GE16942 |
| thioredoxin-like 2 | PICOT; MGC105380 | NM_032614 | 1.538 | GE13361 |
| Threonyl-tRNA synthetase | Tars | AW914860 | 1.497 | GE16474 |
| Thymoma viral proto-oncogene 1 | Akt1 | AA799664 | 1.471 | GE12234 |
| Thyroid hormone receptor associated protein 5 (predicted) | Thrap5_predicted | BG381442 | 1.709 | GE1186469 |
| Thyroid hormone receptor associated protein 6 (predicted) | Thrap6_predicted | CA504616 | 1.443 | GE16435 |
| tight junction protein 2 | ZO-2; MGC124724 | U75916 | 1.422 | GE1196990 |
| Tousled-like kinase 2 (Arabidopsis) (predicted) | Tlk2_predicted | BM386503 | 1.405 | GE1107168 |
| Transcribed locus |  | BF399329 | 3.848 | GE1239597 |
| Transcribed locus |  | CB790337 | 2.679 | GE1224507 |
| Transcribed locus |  | AW530454 | 2.333 | GE1274614 |
| Transcribed locus |  | BI296548 | 2.294 | GE1226554 |
| Transcribed locus |  | BI291868 | 2.055 | GE12560 |
| Transcribed locus | Slc24a2 | CB581086 | 2.051 | GE1158189 |
| Transcribed locus |  | BF388573 | 2.042 | GE19424 |
| Transcribed locus |  | BF416018 | 1.979 | GE1219601 |
| Transcribed locus |  | BM387112 | 1.921 | GE14794 |
| Transcribed locus |  | CA503774 | 1.892 | GE14651 |
| Transcribed locus |  | AW523929 | 1.856 | GE1235464 |
| Transcribed locus |  | AA818376 | 1.852 | GE1272671 |
| Transcribed locus |  | BQ194740 | 1.836 | GE15821 |
| Transcribed locus |  | AW525238 | 1.82 | GE1233953 |
| Transcribed locus |  | AI412099 | 1.759 | GE15323 |
| Transcribed locus |  | BF398673 | 1.749 | GE1263009 |
| Transcribed locus |  | CA510283 | 1.747 | GE1225943 |
| Transcribed locus |  | BQ210650 | 1.741 | GE12740 |
| Transcribed locus |  | AI511180 | 1.721 | GE1249824 |
| Transcribed locus | ProSAPiP1 | AW523114 | 1.709 | GE16182 |
| Transcribed locus |  | BG374180 | 1.699 | GE1273078 |
| Transcribed locus |  | BF402400 | 1.694 | GE1163544 |
| Transcribed locus |  | BF552829 | 1.688 | GE1167981 |
| Transcribed locus | Dap4 | AI072218 | 1.683 | GE13847 |
| Transcribed locus |  | BF567904 | 1.682 | GE19444 |
| Transcribed locus |  | BF525206 | 1.682 | GE1192812 |
| Transcribed locus |  | BF387433 | 1.671 | GE1184256 |
| Transcribed locus |  | BF410147 | 1.657 | GE1174510 |
| Transcribed locus |  | BM390588 | 1.646 | GE1190650 |
| Transcribed locus |  | AW530928 | 1.64 | GE1271094 |
| Transcribed locus |  | BQ200435 | 1.638 | GE1137335 |
| Transcribed locus |  | CA506662 | 1.638 | GE1252327 |
| Transcribed locus |  | AA900746 | 1.637 | GE1233760 |
| Transcribed locus |  | AW916210 | 1.628 | GE16696 |
| Transcribed locus |  | AA957154 | 1.621 | GE1192532 |
| Transcribed locus |  | BE117113 | 1.619 | GE14229 |
| Transcribed locus |  | AW434528 | 1.602 | GE12355 |
| Transcribed locus |  | BI277137 | 1.595 | GE1217561 |
| Transcribed locus |  | BF405110 | 1.59 | GE18744 |
| Transcribed locus |  | BF411657 | 1.585 | GE1270355 |
| Transcribed locus |  | BF402267 | 1.575 | GE1132952 |
| Transcribed locus |  | BF523712 | 1.573 | GE19104 |
| Transcribed locus |  | BI288560 | 1.561 | GE1266348 |
| Transcribed locus |  | BM387678 | 1.558 | GE1125903 |
| Transcribed locus | Csen | BF411924 | 1.557 | GE1213491 |
| Transcribed locus |  | AA925807 | 1.557 | GE14208 |
| Transcribed locus |  | BG381546 | 1.557 | GE17112 |
| Transcribed locus |  | CB585047 | 1.554 | GE1222046 |
| Transcribed locus |  | BE120016 | 1.553 | GE17969 |
| Transcribed locus |  | BF407455 | 1.547 | GE19131 |
| Transcribed locus |  | BI303727 | 1.544 | GE13812 |
| Transcribed locus |  | BF394576 | 1.539 | GE1108245 |
| Transcribed locus |  | CA505674 | 1.539 | GE1110163 |
| Transcribed locus |  | BI293013 | 1.535 | GE1201163 |
| Transcribed locus |  | BF394285 | 1.534 | GE17268 |
| Transcribed locus |  | BF404888 | 1.528 | GE1124618 |
| Transcribed locus |  | CA509483 | 1.525 | GE1178972 |
| Transcribed locus | Parva | AA964535 | 1.524 | GE12960 |
| Transcribed locus |  | AW527814 | 1.522 | GE1124153 |
| Transcribed locus |  | BF405932 | 1.51 | GE18760 |
| Transcribed locus |  | BQ195765 | 1.508 | GE1262149 |
| Transcribed locus | Mgll | AA892864 | 1.504 | GE12678 |
| Transcribed locus |  | BE120675 | 1.504 | GE1233738 |
| Transcribed locus |  | BF548743 | 1.503 | GE19157 |
| Transcribed locus |  | CF112406 | 1.503 | GE1188642 |
| Transcribed locus |  | CB610109 | 1.501 | GE1259616 |
| Transcribed locus |  | BG380796 | 1.5 | GE1114763 |
| Transcribed locus |  | AA956279 | 1.498 | GE1137661 |
| Transcribed locus |  | AW526202 | 1.494 | GE1251081 |
| Transcribed locus |  | AW528898 | 1.49 | GE16309 |
| Transcribed locus |  | BI299766 | 1.486 | GE18678 |
| Transcribed locus |  | BE107744 | 1.485 | GE19117 |
| Transcribed locus |  | AW914942 | 1.484 | GE1156147 |
| Transcribed locus |  | BF388555 | 1.484 | GE1135782 |
| Transcribed locus |  | BF386678 | 1.482 | GE1257582 |
| Transcribed locus |  | BE117527 | 1.481 | GE17056 |
| Transcribed locus |  | BG380825 | 1.479 | GE15891 |
| Transcribed locus |  | BE099787 | 1.475 | GE1250464 |
| Transcribed locus |  | BF401275 | 1.47 | GE18660 |
| Transcribed locus |  | BE107163 | 1.468 | GE1210129 |
| Transcribed locus |  | BE119699 | 1.465 | GE1144799 |
| Transcribed locus |  | BG379606 | 1.464 | GE1208941 |
| Transcribed locus |  | BE112289 | 1.462 | GE1116324 |
| Transcribed locus |  | BU759582 | 1.461 | GE17955 |
| Transcribed locus |  | BF550889 | 1.46 | GE1146679 |
| Transcribed locus |  | BF567521 | 1.458 | GE1193475 |
| Transcribed locus |  | BI275314 | 1.457 | GE13932 |
| Transcribed locus |  | BE111306 | 1.454 | GE17656 |
| Transcribed locus |  | BI300286 | 1.451 | GE14323 |
| Transcribed locus |  | BE117875 | 1.448 | GE1299290 |
| Transcribed locus |  | BI291814 | 1.445 | GE1195241 |
| Transcribed locus |  | AI576132 | 1.444 | GE1157551 |
| Transcribed locus |  | BF402746 | 1.444 | GE1242843 |
| Transcribed locus |  | BF387193 | 1.444 | GE1296061 |
| Transcribed locus | Clcn7 | AA943100 | 1.444 | GE12784 |
| Transcribed locus |  | BF411990 | 1.44 | GE1305876 |
| Transcribed locus |  | BG381511 | 1.436 | GE1182231 |
| Transcribed locus |  | BE112267 | 1.436 | GE14423 |
| Transcribed locus |  | CF113101 | 1.435 | GE1279730 |
| Transcribed locus | Vegf | BF404901 | 1.434 | GE18731 |
| Transcribed locus |  | AI059849 | 1.434 | GE15881 |
| Transcribed locus |  | BE115963 | 1.433 | GE19363 |
| Transcribed locus | Snph | CA510215 | 1.433 | GE1263614 |
| Transcribed locus |  | AA819618 | 1.429 | GE1170612 |
| Transcribed locus |  | BG373835 | 1.429 | GE15908 |
| Transcribed locus |  | BF549260 | 1.428 | GE19161 |
| Transcribed locus | Slc14a1 | BG378796 | 1.427 | GE1191321 |
| Transcribed locus | Anxa7 | AW916534 | 1.423 | GE18130 |
| Transcribed locus |  | BF418640 | 1.422 | GE1198008 |
| Transcribed locus |  | BF388437 | 1.42 | GE1161048 |
| Transcribed locus |  | BQ205729 | 1.417 | GE1255813 |
| Transcribed locus |  | BM391680 | 1.416 | GE1150187 |
| Transcribed locus |  | BE102958 | 1.416 | GE1145587 |
| Transcribed locus |  | CK480180 | 1.413 | GE1257853 |
| Transcribed locus |  | BQ190594 | 1.41 | GE1262604 |
| Transcribed locus |  | BE120370 | 1.409 | GE1130690 |
| Transcribed locus |  | BM391628 | 1.409 | GE1295381 |
| Transcribed locus |  | BI278901 | 1.409 | GE1237554 |
| Transcribed locus |  | AI717256 | 1.401 | GE1206645 |
| Transcribed locus |  | CK477547 | 1.401 | GE1257943 |
| Transcribed locus |  | AI229202 | 0.712 | GE1222244 |
| Transcribed locus |  | AW528868 | 0.706 | GE1156920 |
| Transcribed locus |  | BQ204385 | 0.702 | GE1126956 |
| Transcribed locus |  | BF290961 | 0.701 | GE1265790 |
| Transcribed locus |  | AI549089 | 0.7 | GE1198111 |
| Transcribed locus |  | AW522987 | 0.698 | GE1187321 |
| Transcribed locus |  | BF393546 | 0.696 | GE1222096 |
| Transcribed locus |  | CK477390 | 0.695 | GE1138867 |
| Transcribed locus |  | BE118440 | 0.692 | GE17942 |
| Transcribed locus | Stat5b | BF414366 | 0.69 | GE1188875 |
| Transcribed locus |  | BM389408 | 0.686 | GE1131641 |
| Transcribed locus |  | BI298233 | 0.685 | GE1197372 |
| Transcribed locus |  | BM387569 | 0.684 | GE14213 |
| Transcribed locus | Slc4a4 | BF392259 | 0.679 | GE1252475 |
| Transcribed locus |  | BQ195455 | 0.676 | GE13773 |
| Transcribed locus |  | AI576725 | 0.669 | GE1168329 |
| Transcribed locus |  | BQ209661 | 0.668 | GE1261225 |
| Transcribed locus |  | BF403185 | 0.666 | GE1235811 |
| Transcribed locus |  | BG378073 | 0.665 | GE1149104 |
| Transcribed locus |  | BM386309 | 0.663 | GE17351 |
| Transcribed locus |  | BF398900 | 0.65 | GE1107445 |
| Transcribed locus |  | AW523944 | 0.649 | GE1209182 |
| Transcribed locus |  | BM385386 | 0.633 | GE1207142 |
| Transcribed locus |  | BI295939 | 0.625 | GE12945 |
| Transcribed locus |  | BU946690 | 0.623 | GE1138227 |
| Transcribed locus |  | BQ193016 | 0.606 | GE1191680 |
| Transcribed locus |  | BF413087 | 0.606 | GE1204381 |
| Transcribed locus |  | BG670955 | 0.605 | GE1193996 |
| Transcribed locus |  | BF567991 | 0.59 | GE1225783 |
| Transcribed locus |  | AI073045 | 0.589 | GE1223870 |
| Transcribed locus |  | BQ211223 | 0.587 | GE1242132 |
| Transcribed locus |  | BF414316 | 0.561 | GE1158146 |
| Transcribed locus |  | BI283806 | 0.549 | GE14688 |
| Transcribed locus | RGD1311863 | BE119823 | 0.499 | GE1164752 |
| Transcribed locus |  | AI501441 | 0.495 | GE1169119 |
| Transcribed locus |  | BM384570 | 0.491 | GE15486 |
| Transcribed locus |  | BF564910 | 0.49 | GE1252065 |
| Transcribed locus, moderately similar to NP_203745.1 hypothetical protein LOC286336 [Homo sapiens] |  | BF392407 | 1.785 | GE1143391 |
| Transcribed locus, moderately similar to XP_515579.1 PREDICTED: hypothetical protein XP_515579 [Pan troglodytes] |  | BG378066 | 1.583 | GE1296008 |
| Transcribed locus, moderately similar to XP_546425.2 PREDICTED: similar to roundabout, axon guidance receptor, homolog 3 [Canis familiaris] |  | BF390065 | 1.998 | GE1287744 |
| Transcribed locus, moderately similar to XP_580018.1 PREDICTED: hypothetical protein XP_580018 [Rattus norvegicus] |  | BI274461 | 0.684 | GE1154676 |
| Transcribed locus, strongly similar to NP_003890.1 Rho guanine nucleotide exchange factor 7 isoform a [Homo sapiens] | Pak3bp | BE104891 | 1.433 | GE17327 |
| Transcribed locus, strongly similar to NP_036828.1 kininogen 1 [Rattus norvegicus] | Ak3 | BG374946 | 1.439 | GE14303 |
| Transcribed locus, strongly similar to NP_071949.1 ribosomal protein L36 [Rattus norvegicus] | Rpl36 | BF524750 | 0.598 | GE1108316 |
| Transcribed locus, strongly similar to NP_666281.2 hypothetical protein LOC232164 [Mus musculus] |  | BQ781583 | 1.403 | GE1119215 |
| Transcribed locus, strongly similar to NP_700436.1 gamma tubulin ring complex protein [Mus musculus] |  | AW920761 | 1.457 | GE17060 |
| Transcribed locus, strongly similar to XP_214825.2 PREDICTED: similar to RNA-binding protein Nova-2 |  | BF420504 | 2.381 | GE1236538 |
| Transcribed locus, strongly similar to XP_216537.3 PREDICTED: similar to Expressed sequence AU040320 [Rattus norvegicus] |  | BG377664 | 1.517 | GE15923 |
| Transcribed locus, strongly similar to XP_217613.3 PREDICTED: similar to CXORF15 [Rattus norvegicus] |  | AW529422 | 1.87 | GE1191999 |
| Transcribed locus, strongly similar to XP_223060.3 PREDICTED: similar to LEK1 [Rattus norvegicus] |  | BQ190232 | 0.68 | GE18554 |
| Transcribed locus, strongly similar to XP_223687.3 PREDICTED: similar to RIKEN cDNA 4933435A13 [Rattus norvegicus] |  | AW144646 | 1.475 | GE15972 |
| Transcribed locus, strongly similar to XP_225257.2 PREDICTED: similar to Thioredoxin domain containing protein 5 precursor (Thioredoxin-like protein p46) (Endoplasmic reticulum protein ERp46) [Rattus norvegicus] |  | H31374 | 1.442 | GE1294848 |
| Transcribed locus, strongly similar to XP_226471.2 PREDICTED: similar to Mtr3 (mRNA transport regulator 3)-homolog [Rattus norvegicus] |  | AW251819 | 0.616 | GE1227353 |
| Transcribed locus, strongly similar to XP_226550.3 PREDICTED: similar to Kelch domain containing 4 [Rattus norvegicus] |  | CK469848 | 1.713 | GE1194318 |
| Transcribed locus, strongly similar to XP_236620.3 PREDICTED: similar to RIKEN cDNA 6430571L13 gene [Rattus norvegicus] |  | BF552415 | 1.424 | GE1215538 |
| Transcribed locus, strongly similar to XP_342709.2 PREDICTED: similar to receptor expression enhancing protein 1 [Rattus norvegicus] |  | CA505336 | 1.467 | GE18244 |
| Transcribed locus, strongly similar to XP_343328.1 PREDICTED: desert hedgehog homolog [Rattus norvegicus] | Dhh | AW529714 | 0.704 | GE1179703 |
| Transcribed locus, strongly similar to XP_523419.1 PREDICTED: similar to zinc and ring finger protein 1; nerve injury gene 283 [Pan troglodytes] | Ldhd | BI286754 | 1.612 | GE19102 |
| Transcribed locus, strongly similar to XP_537630.2 PREDICTED: similar to CG10802-PA [Canis familiaris] |  | BF524517 | 1.421 | GE15492 |
| Transcribed locus, strongly similar to XP_574462.1 PREDICTED: similar to hypothetical protein C230069C04 [Rattus norvegicus] |  | BE111548 | 1.417 | GE1296589 |
| Transcribed locus, strongly similar to XP_576460.1 PREDICTED: similar to hypothetical protein PB402898.00.0 [Rattus norvegicus] |  | BM391969 | 1.436 | GE1245867 |
| Transcribed locus, strongly similar to XP_579767.1 PREDICTED: hypothetical protein XP_579767 [Rattus norvegicus] |  | BF551531 | 1.468 | GE1102439 |
| Transcribed locus, strongly similar to XP_579953.1 PREDICTED: hypothetical protein XP_579953 [Rattus norvegicus] | Slk | CK227972 | 1.413 | GE19286 |
| Transcribed locus, strongly similar to XP_580013.1 PREDICTED: hypothetical protein XP_580013 [Rattus norvegicus] |  | AW920546 | 0.594 | GE1136098 |
| Transcribed locus, strongly similar to XP_580081.1 PREDICTED: hypothetical protein XP_580081 [Rattus norvegicus] |  | CK359848 | 1.636 | GE1293956 |
| Transcribed locus, weakly similar to NP_001003141.1 protein tyrosine kinase fer [Canis familiaris] |  | BE117327 | 0.657 | GE16643 |
| Transcribed locus, weakly similar to NP_473118.2 hypothetical protein [Plasmodium falciparum 3D7] |  | BQ202905 | 1.466 | GE1294215 |
| Transcribed locus, weakly similar to XP_342574.1 PREDICTED: similar to Csr1 [Rattus norvegicus] |  | BF403856 | 1.516 | GE1232431 |
| Transcribed locus, weakly similar to XP_421392.1 PREDICTED: similar to Protein phosphatase 1, regulatory (inhibitor) subunit 13B [Gallus gallus] |  | BM391983 | 1.598 | GE1279021 |
| Transcribed locus, weakly similar to XP_512872.1 PREDICTED: similar to Zinc finger protein 83 (HPF1) [Pan troglodytes] |  | AI236841 | 2.109 | GE1165363 |
| Transcribed locus, weakly similar to XP_529735.1 PREDICTED: hypothetical protein XP_529735 [Pan troglodytes] |  | CB782037 | 0.647 | GE1244031 |
| Transcribed locus, weakly similar to XP_576810.1 PREDICTED: similar to RIKEN cDNA 4930555G01 [Rattus norvegicus] |  | CB606098 | 1.638 | GE1219990 |
| Transcribed locus, weakly similar to XP_580018.1 PREDICTED: hypothetical protein XP_580018 [Rattus norvegicus] |  | BQ190641 | 2.024 | GE1255843 |
| Transcribed locus, weakly similar to XP_580018.1 PREDICTED: hypothetical protein XP_580018 [Rattus norvegicus] |  | BI278180 | 1.445 | GE1248493 |
| transcription factor 4 | Tcf4 | NM_053369 | 1.589 | GE20851 |
| Transcription factor CP2-like 4 (predicted) | Tfcp2l4_predicted | BE109781 | 1.551 | GE1169834 |
| Transcription factor Pur-beta | pur-beta | BF399993 | 1.435 | GE18637 |
| Transducer of ERBB2, 2 | Tob2 | CA505243 | 1.48 | GE1108322 |
| Transforming, acidic coiled-coil containing protein 1 | Tacc1 | BG373358 | 1.401 | GE1171330 |
| Transient receptor potential cation channel, subfamily C, member 4 associated protein | Trpc4ap | BM390154 | 1.565 | GE1181121 |
| translocase of outer mitochondrial membrane 20 homolog (yeast) | MGC93136 | NM_152935 | 1.541 | GE21935 |
| Transmembrane emp24 protein transport domain containing 9 | Tmed9 | CA508371 | 1.444 | GE1213656 |
| Transmembrane protein 2 (predicted) | Tmem2_predicted | CA506692 | 1.428 | GE1128197 |
| Treacher Collins Franceschetti syndrome 1, homolog (predicted) | Tcof1_predicted | AA996517 | 0.684 | GE12972 |
| Tribbles homolog 1 (Drosophila) | Trib1 | BQ196607 | 1.887 | GE1100517 |
| Tripartite motif protein 28 | Trim28 | BE104143 | 1.429 | GE17313 |
| tripartite motif protein 3 | Berp; Rnf22 | NM_031786 | 1.421 | GE1185629 |
| Tropomyosin 3, gamma | Tpm3 | BF406206 | 1.546 | GE1175093 |
| tryptase gamma 1 | Tpsg1 | NM_175593 | 0.59 | GE1155399 |
| Tryptophanyl-tRNA synthetase | Wars | BE110929 | 1.472 | GE1268362 |
| Tuftelin interacting protein 11 | Tfip11 | BI295096 | 1.977 | GE1130656 |
| Tumor protein p53 inducible protein 13 (predicted) | Trp53i13_predicted | H35532 | 1.693 | GE14715 |
| Tweety homolog 3 (Drosophila) (predicted) | Ttyh3_predicted | CA512117 | 0.549 | GE1213706 |
| Type I keratin KA15 | Ka15 | AW529723 | 0.471 | GE16317 |
| tyrosine 3-monooxygenase/tryptophan 5-monooxygenase activation protein, eta polypeptide | 14-3-3e; MGC93547 | NM_013052 | 1.575 | GE20141 |
| Tyrosyl-tRNA synthetase 2 (mitochondrial) | Yars2 | BQ206370 | 1.495 | GE1231356 |
| Ubiquinol-cytochrome c reductase hinge protein | Uqcrh | BM391636 | 1.513 | GE18506 |
| Ubiquitin carboxyl-terminal esterase L3 (ubiquitin thiolesterase) | Uchl3 | BF415734 | 1.408 | GE1105086 |
| Ubiquitin specific peptidase 9, X chromosome (predicted) | Usp9x_predicted | CA513386 | 1.42 | GE1175798 |
| UDP glycosyltransferase 1 family, polypeptide A8 | Ugt1; Ugt1a9 | NM_175846 | 2.199 | GE1265904 |
| UDP-glucuronate decarboxylase 1 | UGD; MGC93157 | NM_139336 | 1.556 | GE1107316 |
| UDP-N-acetyl-alpha-D-galactosamine:polypeptide N-acetylgalactosaminyltransferase 9 (predicted) | Galnt9_predicted | CB773332 | 1.663 | GE1176056 |
| UDP-N-acetyl-alpha-D-galactosamine:polypeptide N-acetylgalactosaminyltransferase-like 1 | Galntl1 | CB773272 | 1.449 | GE1225431 |
| UI-R-BJ1-awc-b-10-0-UI.s1 UI-R-BJ1 Rattus norvegicus cDNA clone UI-R-BJ1-awc-b-10-0-UI 3', mRNA sequence. |  | BE113157 | 0.706 | GE17769 |
| UI-R-BJ1-azo-g-06-0-UI.s1 UI-R-BJ1 Rattus norvegicus cDNA clone UI-R-BJ1-azo-g-06-0-UI 3', mRNA sequence. |  | BE118277 | 1.436 | GE12588 |
| UI-R-BJ2-bqa-e-05-0-UI.s1 UI-R-BJ2 Rattus norvegicus cDNA clone UI-R-BJ2-bqa-e-05-0-UI 3', mRNA sequence. |  | BF420813 | 1.595 | GE1188140 |
| UI-R-BT1-ako-a-10-0-UI.r1 UI-R-BT1 Rattus norvegicus cDNA clone UI-R-BT1-ako-a-10-0-UI 5', mRNA sequence. |  | BF565629 | 0.621 | GE1185048 |
| UI-R-C0-gx-d-08-0-UI.r1 UI-R-C0 Rattus norvegicus cDNA clone UI-R-C0-gx-d-08-0-UI 5', mRNA sequence. |  | BF560852 | 0.552 | GE1275386 |
| UI-R-C2p-qz-f-05-0-UI.r1 UI-R-C2p Rattus norvegicus cDNA clone UI-R-C2p-qz-f-05-0-UI 5', mRNA sequence. |  | BF522996 | 1.59 | GE1280874 |
| UI-R-CW0-bwn-h-04-0-UI.s1 UI-R-CW0 Rattus norvegicus cDNA clone UI-R-CW0-bwn-h-04-0-UI 3', mRNA sequence. |  | BI276081 | 0.639 | GE15231 |
| UI-R-DM1-ckd-n-01-0-UI.s1 UI-R-DM1 Rattus norvegicus cDNA clone UI-R-DM1-ckd-n-01-0-UI 3', mRNA sequence. |  | BM385621 | 0.617 | GE1142003 |
| UI-R-DZ1-cnm-c-22-0-UI.s1 NCI_CGAP_DZ1 Rattus norvegicus cDNA clone IMAGE:7347504 3', mRNA sequence. |  | BQ206808 | 0.408 | GE1229311 |
| UI-R-DZ1-cnm-g-20-0-UI.s1 NCI_CGAP_DZ1 Rattus norvegicus cDNA clone IMAGE:7347598 3', mRNA sequence. |  | BQ206828 | 1.617 | GE12390 |
| UI-R-E1-fe-b-07-0-UI.s1 UI-R-E1 Rattus norvegicus cDNA clone UI-R-E1-fe-b-07-0-UI 3', mRNA sequence. |  | AA956031 | 0.572 | GE1116227 |
| UI-R-E1-fy-b-11-0-UI.s1 UI-R-E1 Rattus norvegicus cDNA clone UI-R-E1-fy-b-11-0-UI 3', mRNA sequence. |  | AA957361 | 0.661 | GE1236005 |
| UI-R-FS0-cry-k-23-0-UI.s1 NCI_CGAP_FS0 Rattus norvegicus cDNA clone IMAGE:7361137 3', mRNA sequence. |  | CB327893 | 1.402 | GE1119735 |
| UI-R-Y0-vk-b-12-0-UI.r1 UI-R-Y0 Rattus norvegicus cDNA clone UI-R-Y0-vk-b-12-0-UI 5', mRNA sequence. |  | BF522813 | 0.536 | GE1183543 |
| Uncharacterized protein family UPF0227 member RGD1359682 | RGD1359682 | BE118442 | 1.625 | GE1121042 |
| upstream binding transcription factor, RNA polymerase I | Tcfubf | M61726 | 1.995 | GE19718 |
| Vacuolar protein sorting 37B (yeast) (predicted) | Vps37b_predicted | BG377624 | 1.647 | GE1243870 |
| Vacuolar protein sorting 4a (yeast) | Vps4a | AA894099 | 1.74 | GE12718 |
| Vascular early response gene protein | Verge | AW523915 | 2.677 | GE1279845 |
| Vesicle amine transport protein 1 homolog (T californica) | Vat1 | BF414142 | 1.482 | GE1225537 |
| Vesicle transport through interaction with t-SNAREs homolog 1A (yeast) | Vti1a | CB730537 | 1.513 | GE1224645 |
| vitamin K epoxide reductase complex, subunit 1-like 1 | Vkorc1l1 | NM_203338 | 1.519 | GE1158827 |
| WD repeat domain 32 (predicted) | Wdr32_predicted | AA944709 | 1.624 | GE1103965 |
| WD repeat domain 39 | Wdr39 | BI295885 | 1.446 | GE1242643 |
| WW domain containing E3 ubiquitin protein ligase 2 (predicted) | Wwp2_predicted | BF551945 | 0.639 | GE1150970 |
| X Kell blood group precursor related family member 8 homolog | Xkr8 | AI407409 | 1.466 | GE15067 |
| Y box protein 1 | Yb1; Byb1; Cbfa; Dbpb; Ef1a; Msy1; Nsep1 | NM_031563 | 1.457 | GE19732 |
| Yip1 interacting factor homolog (S. cerevisiae) | Yif1 | AI137644 | 0.709 | GE22077 |
| YTH domain family 2 (predicted) | Ythdf2_predicted | AW535358 | 2.512 | GE16404 |
| Zinc finger and BTB domain containing 16 | Zbtb16 | BF409027 | 1.645 | GE1257656 |
| Zinc finger homeobox 2 | Zfhx2 | BE118113 | 1.766 | GE15661 |
| zinc finger protein 148 | Zbp-89; Znf148 | NM_031615 | 1.646 | GE20903 |
| Zinc finger protein 189 (predicted) | Zfp189_predicted | BG671710 | 1.702 | GE12289 |
| zinc finger protein 238 | Rp58 | NM_022678 | 1.553 | GE1248713 |
| Zinc finger protein 297 | Zfp297 | AW921218 | 1.707 | GE17076 |
| Zinc finger protein 410 (predicted) | Zfp410_predicted | AA957730 | 1.587 | GE1293437 |
| Zinc finger protein 523 (predicted) | Zfp523_predicted | AI102904 | 1.524 | GE13949 |
| zinc finger protein 99 | Rlzfy; MGC114256 | NM_145724 | 1.469 | GE21360 |
| Zinc finger, DHHC domain containing 8 | Zdhhc8 | BM390303 | 1.404 | GE1285219 |
| Zinc finger, FYVE domain containing 20 (predicted) | Zfyve20_predicted | AA901014 | 1.444 | GE1107211 |
| Zinc fingers and homeoboxes 3 | Zhx3 | AA891165 | 1.57 | GE1101957 |
| ZUBR1 | Rbaf600 | CA507471 | 1.462 | GE17339 |

**mPFC**

**Naïve vs. 100-day**

| **Gene** | **Alias** | **Accession #** | **Fold Change** | **Probe ID** |
| --- | --- | --- | --- | --- |
| a disintegrin and metalloprotease domain 3 (cyritestin) | tMDCI | NM_020302 | 0.666 | GE20730 |
| acrosin | Acro | NM_012490 | 0.663 | GE19780 |
| actin related protein 2/3 complex, subunit 1B | Arpc1b | NM_019289 | 0.271 | GE20638 |
| activity regulated cytoskeletal-associated protein | rg3.1 | NM_019361 | 1.932 | GE20683 |
| Acyl-Coenzyme A dehydrogenase family, member 9 | Acad9 | CK601387 | 0.69 | GE1127239 |
| acyl-Coenzyme A oxidase 1, palmitoyl | RATACOA1 | NM_017340 | 0.649 | GE21638 |
| Adenylate cyclase 7 | Adcy7 | CB759352 | 0.462 | GE1215923 |
| adenylate cyclase activating polypeptide 1 receptor 1 | Adcyap1r1 | NM_133511 | 0.479 | GE19473 |
| adenylate kinase 2 | Ak2 | NM_030986 | 1.918 | GE21558 |
| Adenylosuccinate lyase (predicted) | Adsl_predicted | AI763587 | 1.425 | GE1263292 |
| AGENCOURT_17182824 NIH_MGC_233 Rattus norvegicus cDNA clone IMAGE:7101024 5', mRNA sequence. | Grlf1_predicted | CK359453 | 0.639 | GE1215330 |
| AGENCOURT_17578897 NIH_MGC_232 Rattus norvegicus cDNA clone IMAGE:7124785 5', mRNA sequence. |  | CK477071 | 0.541 | GE1299668 |
| AGENCOURT_17638475 NIH_MGC_235 Rattus norvegicus cDNA clone IMAGE:7107569 5', mRNA sequence. |  | CK481115 | 0.621 | GE1151175 |
| aldo-keto reductase family 1, member D1 | Akr1d1 | NM_138884 | 0.705 | GE1253447 |
| AlkB, alkylation repair homolog 5 (E. coli) (predicted) | Alkbh5_predicted | BQ203074 | 0.613 | GE1250528 |
| AMGNNUC:NRDG1-00003-G3-A nrdg1 (10855) Rattus norvegicus cDNA clone nrdg1-00003-g3 5', mRNA sequence. | Amigo | CB547987 | 1.447 | GE1169415 |
| AMGNNUC:NRDG1-00145-E11-A nrdg1 (10855) Rattus norvegicus cDNA clone nrdg1-00145-e11 5', mRNA sequence. |  | CB582467 | 0.647 | GE1289178 |
| AMGNNUC:NRHY1-00111-C2-A W Rat hypothalamus (10480) Rattus norvegicus cDNA clone nrhy1-00111-c2 5', mRNA sequence. |  | CB544357 | 0.685 | GE1279272 |
| AMGNNUC:NRHY5-00234-A11-A W Rat hypothalamus (10471) Rattus norvegicus cDNA clone nrhy5-00234-a11 5', mRNA sequence. |  | CB583629 | 0.651 | GE1192718 |
| AMGNNUC:NRHY5-00404-B11-A W Rat hypothalamus (10471) Rattus norvegicus cDNA clone nrhy5-00404-b11 5', mRNA sequence. |  | CB608757 | 0.663 | GE1115690 |
| AMGNNUC:NRHY5-00407-H1-A W Rat hypothalamus (10471) Rattus norvegicus cDNA clone nrhy5-00407-h1 5', mRNA sequence. |  | CB607888 | 0.654 | GE1269772 |
| AMGNNUC:NRHY7-00040-D4-A nrhy7 (10850) Rattus norvegicus cDNA clone nrhy7-00040-d4 5', mRNA sequence. |  | CB612893 | 0.487 | GE1198670 |
| AMGNNUC:NRHY7-00041-G1-A nrhy7 (10850) Rattus norvegicus cDNA clone nrhy7-00041-g1 5', mRNA sequence. |  | CB582955 | 0.503 | GE1235739 |
| AMGNNUC:NRHY7-00043-A10-A nrhy7 (10850) Rattus norvegicus cDNA clone nrhy7-00043-a10 5', mRNA sequence. | LOC689479 | CB586104 | 0.681 | GE1110368 |
| AMGNNUC:NRPI4-00014-E2-A W Rat pituitary (10472) Rattus norvegicus cDNA clone nrpi4-00014-e2 5', mRNA sequence. |  | CB774187 | 0.608 | GE1248166 |
| AMGNNUC:NRWA3-00067-G6-A white adipose tiss (10469) Rattus norvegicus cDNA clone nrwa3-00067-g6 5', mRNA sequence. |  | CB749234 | 0.702 | GE1238867 |
| Androgen receptor-related apoptosis-associated protein CBL27 | Cbl27 | AI501035 | 1.66 | GE1209550 |
| Ankyrin 3, epithelial | Ank3 | BF392810 | 1.414 | GE1297394 |
| Ankyrin repeat and MYND domain containing 2 (predicted) | Ankmy2_predicted | BF560671 | 1.788 | GE1251814 |
| Ankyrin repeat and SOCS box-containing protein 11 (predicted) | Asb11_predicted | BF284816 | 0.697 | GE18260 |
| Ankyrin repeat and SOCS box-containing protein 6 | Asb6 | BM387956 | 1.414 | GE18464 |
| Anterior pharynx defective 1a homolog (C. elegans) | Aph1a | BF411371 | 1.752 | GE16545 |
| Apolipoprotein M | Apom | Z83066 | 1.408 | GE21933 |
| aquaporin 6 | Aqp6 | NM_022181 | 0.654 | GE1301850 |
| Arsenic (+3 oxidation state) methyltransferase | As3mt | CK227096 | 0.599 | GE1203640 |
| Aryl-hydrocarbon receptor repressor | Ahrr | BI304155 | 0.611 | GE1201179 |
| Ataxin 10 | Atxn10 | BF404085 | 1.964 | GE1206673 |
| ATP synthase, H+ transporting, mitochondrial F1 complex, epsilon subunit | Atp5e | NM_139099 | 1.716 | GE1202898 |
| ATPase, Ca++ transporting, type 2C, member 2 | Spca2 | NM_134462 | 0.472 | GE1266022 |
| ATP-binding cassette, sub-family C (CFTR/MRP), member 1 | Abcc1 | CB578695 | 0.189 | GE1220959 |
| B-cell leukemia/lymphoma 3 (predicted) | Bcl3_predicted | AW532224 | 2.087 | GE1180697 |
| BMP and activin membrane-bound inhibitor, homolog (Xenopus laevis) | Bambi | NM_139082 | 2.089 | GE18823 |
| brain abundant, membrane attached signal protein 1 | NAP22 | NM_022300 | 1.447 | GE1197450 |
| Brain-specific angiogenesis inhibitor 3 (predicted) | Bai3_predicted | BF401132 | 1.469 | GE18670 |
| BTB (POZ) domain containing 14A | Btbd14a | BF414548 | 0.623 | GE1225273 |
| C1q and tumor necrosis factor related protein 4 (predicted) | C1qtnf4_predicted | AW918311 | 1.513 | GE16897 |
| C1q and tumor necrosis factor related protein 7 (predicted) | C1qtnf7_predicted | AI501709 | 0.651 | GE1117178 |
| Cadherin 11 | Cdh11 | BQ208832 | 1.445 | GE1220820 |
| Cadherin 16 | Cdh16 | AA891858 | 0.624 | GE12623 |
| Cadherin 2 | Cdh2 | BF388128 | 1.93 | GE1303179 |
| calcitonin/calcitonin-related polypeptide, alpha | CAL6; CGRP; Cal1; Calc; RATCAL6; calcitonin | NM_017338 | 0.0796 | GE22081 |
| calcium channel, voltage-dependent, beta 3 subunit | CACH3B | NM_012828 | 1.483 | GE19992 |
| calcium/calmodulin-dependent protein kinase II inhibitor 1 | Camk2n1 | NM_173337 | 1.476 | GE13529 |
| calcium/calmodulin-dependent protein kinase II, alpha | PKCCD; PK2CDD | AF237778 | 1.783 | GE13501 |
| calcium/calmodulin-dependent protein kinase II, beta | Ck2b | NM_021739 | 1.906 | GE20786 |
| calcium-sensing receptor | Casr | NM_016996 | 1.584 | GE20263 |
| Caldesmon 1 | Cald1 | AW532335 | 0.679 | GE1111716 |
| Caldesmon 1 | Cald1 | BF400764 | 0.432 | GE1268528 |
| calponin 1 | Cnn1 | NM_031747 | 0.425 | GE22181 |
| CAMP responsive element binding protein 3-like 2 | Creb3l2 | CB741722 | 0.238 | GE1281249 |
| carcinoembryonic antigen-related cell adhesion molecule 3 | Cgm1; RATCEAA; MGC114355 | NM_012702 | 0.581 | GE19911 |
| Carnitine palmitoyltransferase 2 | Cpt2 | BF551857 | 0.214 | GE1180423 |
| casein beta | Csnb | NM_017120 | 0.711 | GE1275746 |
| CASK-interacting protein CIP98 | Cip98 | AW530332 | 1.409 | GE16327 |
| Catenin (cadherin-associated protein), alpha 1 | Catna1 | AA817722 | 1.514 | GE12339 |
| Cathepsin C | Ctsc | AA858815 | 0.536 | GE1236503 |
| Cathepsin K | Ctsk | BE121118 | 0.663 | GE1288902 |
| CD302 antigen | Cd302 | BM386753 | 0.675 | GE16655 |
| CD47 antigen (Rh-related antigen, integrin-associated signal transducer) | Cd47 | AW141154 | 2.954 | GE21321 |
| CDC-like kinase 3 | Clk3 | NM_134340 | 1.402 | GE22207 |
| CDNA BC060737 (predicted) | BC060737_predicted | CK365821 | 0.29 | GE1161677 |
| CDNA clone IMAGE:7103416 |  | CK355741 | 0.586 | GE1204068 |
| CDNA clone IMAGE:7303896 |  | H34951 | 1.414 | GE15391 |
| CDNA clone IMAGE:7309127 |  | CB736048 | 0.675 | GE1148293 |
| CDNA clone IMAGE:7324860 | MGC94226 | BU759303 | 1.431 | GE1271845 |
| CDNA clone IMAGE:7375734 |  | CB791260 | 2.069 | GE1124365 |
| CDNA clone MGC:95041 IMAGE:7123253 |  | BE113935 | 1.506 | GE19018 |
| CEA-related cell adhesion molecule 12 (predicted) | Ceacam12_predicted | CB613595 | 0.685 | GE1251214 |
| Cellular repressor of E1A-stimulated genes (predicted) | Creg_predicted | BE112223 | 0.657 | GE15784 |
| Centaurin, gamma 1 | Centg1 | AW526039 | 1.574 | GE16248 |
| Checkpoint suppressor 1 (predicted) | Ches1_predicted | CB578537 | 0.579 | GE1142600 |
| chemokine (C-C motif) receptor 3 | Cmkbr3 | NM_053958 | 0.393 | GE22239 |
| chemokine binding protein 2 | D6; MGC105345 | NM_078621 | 0.581 | GE22070 |
| Chemokine orphan receptor 1 | Cmkor1 | AI029016 | 3.268 | GE1126478 |
| CHK2 checkpoint homolog (S. pombe) | Chk2; Rad53 | NM_053677 | 0.52 | GE13370 |
| Cholinergic receptor, muscarinic 3 | Chrm3 | BF411828 | 1.552 | GE1137759 |
| cholinergic receptor, nicotinic, alpha polypeptide 5 | Chrna5 | NM_017078 | 0.71 | GE20324 |
| chondroitin sulfate proteoglycan 3 | neurocan | NM_031653 | 1.455 | GE19775 |
| claudin 11 | Cldn11 | NM_053457 | 1.509 | GE13590 |
| Cnksr family member 3 | Cnksr3 | BM386248 | 0.517 | GE1201634 |
| Coiled-coil domain containing 37 (predicted) | Ccdc37_predicted | CA509611 | 0.681 | GE1103230 |
| Coilin | Coil | BF398409 | 0.688 | GE1252497 |
| CUB and Sushi multiple domains 1 | Csmd1 | BF410323 | 1.993 | GE1273177 |
| cut-like 1 (Drosophila) | Cutl1 | U09229 | 1.63 | GE1175878 |
| CXXC finger 5 | Cxxc5 | H33001 | 1.475 | GE15397 |
| Cyclin D3 | Ccnd3 | BI280392 | 1.615 | GE1112083 |
| cyclin D3 | MGC93643; MGC108760 | NM_012766 | 1.419 | GE1148424 |
| Cytochrome b5 reductase 4 | Cyb5r4 | AI145486 | 1.564 | GE1152529 |
| Cytochrome c oxidase, subunit VIa, polypeptide 1 | Cox6a1 | BM387708 | 1.426 | GE1269464 |
| Cytochrome c oxidase, subunit VIIIa | Cox8a | BE116231 | 1.52 | GE1137837 |
| cytochrome P450, family 1, subfamily a, polypeptide 2 | CYPD45; P-450d; RATCYPD45 | NM_012541 | 0.451 | GE21665 |
| cytochrome P450, subfamily 3A, polypeptide 3 | CYP; RL33; cDEX; CYP3A1; CYP3A23; MGC108757 | NM_013105 | 0.583 | GE21745 |
| Cytochrome P450-like protein | Loc266761 | AI556897 | 0.364 | GE1152017 |
| DEAD (Asp-Glu-Ala-Asp) box polypeptide 50 | Ddx50 | BF389731 | 1.469 | GE1223116 |
| decidual/trophoblast prolactin-related protein | Dprp; d/tPRP | NM_022846 | 0.315 | GE1246761 |
| decorin | MGC94682 | X59859 | 0.61 | GE22148 |
| dihydropyrimidinase-like 4 | Crmp3; Dpys4 | U52103 | 0.671 | GE20960 |
| dihydropyrimidine dehydrogenase | Dpyd | NM_031027 | 0.463 | GE21615 |
| discs, large homolog 4 (Drosophila) | PSD95; Sap90 | NM_019621 | 0.71 | GE22051 |
| DnaJ (Hsp40) related, subfamily B, member 13 | Dnajb13 | BF406540 | 1.458 | GE1212239 |
| dopamine receptor D4 | D4RA | NM_012944 | 0.653 | GE21846 |
| dopamine receptor D5 | Drd5 | NM_012768 | 0.406 | GE19955 |
| DRACOB08 Rat DRG Library Rattus norvegicus cDNA clone DRACOB08 5', mRNA sequence. |  | BG666106 | 1.492 | GE13896 |
| D-serine modulator-1 | Dsm-1 | AA944561 | 1.817 | GE12850 |
| Dual endothelin 1, angiotensin II receptor | Dear | CA506902 | 1.705 | GE17632 |
| dual specificity phosphatase 1 | Mkp1; CL100; MKP-1; 3CH134; Ptpn16 | NM_053769 | 1.611 | GE1248349 |
| Dual specificity phosphatase 9 | Dusp9 | BE116394 | 0.645 | GE1206064 |
| Dynactin 1 | Dctn1 | AA964012 | 2.382 | GE1188053 |
| dynamin 2 | DYIIAAB | NM_013199 | 0.571 | GE20243 |
| dynamin 3 | dynamin-2 | NM_138538 | 1.472 | GE1174180 |
| dynein cytoplasmic 1 light intermediate chain 1 | Dncli1; Dnclic1 | NM_145772 | 0.494 | GE13434 |
| Early growth response 3 | Egr3 | BF410052 | 1.495 | GE1265585 |
| Eph receptor A7 | Epha7 | NM_134331 | 1.465 | GE1295941 |
| Eph receptor B1 | Ephb1 | BE119691 | 1.595 | GE1273675 |
| EST224191 Normalized rat spleen, Bento Soares Rattus sp. cDNA clone RSPCX78 3' end, mRNA sequence. |  | AI180445 | 0.394 | GE1139734 |
| EST224922 Normalized rat brain, Bento Soares Rattus sp. cDNA clone RBRCS15 3' end, mRNA sequence. |  | AI228227 | 0.428 | GE1123093 |
| EST240215 Normalized rat kidney, Bento Soares Rattus sp. cDNA clone RKIER55 3' end, mRNA sequence. |  | AI411921 | 0.697 | GE1279773 |
| EST350850 Rat gene index, normalized rat, norvegicus, Bento Soares Rattus norvegicus cDNA clone RGIFY73 5' end, mRNA sequence. |  | AW919546 | 0.606 | GE1262370 |
| EST351688 Rat gene index, normalized rat, norvegicus, Bento Soares Rattus norvegicus cDNA clone RGIGU94 5' end, mRNA sequence. |  | AW920384 | 0.659 | GE1288290 |
| EST352073 Rat gene index, normalized rat, norvegicus, Bento Soares Rattus norvegicus cDNA clone RGIHC11 5' end, mRNA sequence. |  | AW920769 | 0.626 | GE17062 |
| EST352151 Rat gene index, normalized rat, norvegicus, Bento Soares Rattus norvegicus cDNA clone RGIHD82 5' end, mRNA sequence. |  | AW920847 | 2.929 | GE1230031 |
| EST453765 Rat Gene Index, normalized rat, Rattus norvegicus cDNA Rattus norvegicus cDNA clone RGIHB92 3' sequence, mRNA sequence. |  | BF289174 | 0.674 | GE1226341 |
| EST455770 Rat Gene Index, normalized rat, Rattus norvegicus cDNA Rattus norvegicus cDNA clone RGIIJ72 3' sequence, mRNA sequence. |  | BF291179 | 0.563 | GE1129670 |
| EST531153 Rat gene index, normalized rat, norvegicus Rattus norvegicus cDNA clone RGIAA90 5' end similar to mitochondrial D-loop nascent strand-like region |  | BM986373 | 0.655 | GE1170372 |
| estrogen receptor 2 beta | Erb2; ERbeta; ER-beta | NM_012754 | 0.712 | GE21254 |
| Ets variant gene 4 (E1A enhancer binding protein, E1AF) (predicted) | Etv4_predicted | AW253928 | 1.401 | GE16074 |
| Eukaryotic translation initiation factor 5B | Eif5b | BF523090 | 0.667 | GE1276284 |
| Exocyst complex component 4 | Exoc4 | CA507773 | 0.676 | GE1195881 |
| Far upstream element (FUSE) binding protein 1 | Fubp1 | BF407775 | 1.435 | GE17389 |
| farnesyltransferase, CAAX box, beta | MGC105303 | NM_172034 | 1.409 | GE19731 |
| FBJ murine osteosarcoma viral oncogene homolog | Fos | BF415939 | 1.534 | GE18981 |
| FBJ murine osteosarcoma viral oncogene homolog | Fos | AW915240 | 1.501 | GE16552 |
| F-box protein 46 | Fbxo46 | BF564168 | 0.474 | GE1288733 |
| FGF receptor activating protein 1 | PGAP2 | NM_053895 | 1.477 | GE22009 |
| Fibroblast activation protein | Fap | BQ203726 | 0.559 | GE1101999 |
| Fibroblast growth factor receptor substrate 3 | Frs3 | BF416732 | 0.713 | GE1292727 |
| Fibronectin type III and SPRY domain containing 2 (predicted) | Fsd2_predicted | AI714110 | 0.683 | GE1133626 |
| Forkhead box O3a (predicted) | Foxo3a_predicted | AI231684 | 0.7 | GE14766 |
| Forty-two-three domain containing 1 | Fyttd1 | BF406646 | 1.469 | GE18782 |
| fumarylacetoacetate hydrolase | Fah | NM_017181 | 0.563 | GE20401 |
| G kinase anchoring protein 1 | Gkap1 | CB783308 | 0.276 | GE1206517 |
| GA repeat binding protein, alpha (predicted) | Gabpa_predicted | CA512584 | 0.492 | GE1266545 |
| gastrin releasing peptide receptor | Grpr | NM_012706 | 0.673 | GE22135 |
| glutamate cysteine ligase, modifier subunit | Glclr | NM_017305 | 1.43 | GE20488 |
| glutamate receptor, ionotropic, N-methyl D-aspartate 2B | Grin2b | NM_012574 | 1.854 | GE1205376 |
| Glutaminase 2 (liver, mitochondrial) | Gls2 | CB606833 | 0.64 | GE1234987 |
| Glycerol-3-phosphate dehydrogenase 1 (soluble) | Gpd1 | BI277358 | 1.459 | GE1166453 |
| glycogen synthase kinase 3 beta | Gsk3b | NM_032080 | 1.504 | GE21138 |
| glycoprotein 2 (zymogen granule membrane) | Gp2 | NM_134418 | 0.703 | GE1137686 |
| Glycoprotein 49b | Gp49b | CA508780 | 0.541 | GE1222504 |
| Glypican 4 | Gpc4 | CK483936 | 0.479 | GE1286745 |
| gonadotropin releasing hormone receptor | Lhrhr | NM_031038 | 0.59 | GE1115963 |
| GTPase, IMAP family member 7 | Gimap7; Ian3; MGC108919 | BM388319 | 0.569 | GE1164596 |
| guanine nucleotide binding protein, alpha inhibiting 2 | Galphai2 | NM_031035 | 1.421 | GE1126245 |
| Guanine nucleotide binding protein, alpha o | Gnao | BF287080 | 1.461 | GE1250065 |
| Gup1, glycerol uptake/transporter homolog (yeast) (predicted) | Gup1_predicted | AI172352 | 1.561 | GE14360 |
| heat shock 70kDa protein 5 binding protein 1 | Gbp | NM_178021 | 1.405 | GE1154612 |
| Hermansky-Pudlak syndrome 1 homolog (human) | Hps1 | CK222076 | 0.684 | GE1158633 |
| high mobility group AT-hook 1 | Hmgi; Hmgiy | X62875 | 1.471 | GE1132911 |
| hippocalcin-like 4 | NVP-2; Nvjp2 | D14819 | 0.677 | GE21571 |
| HMT1 hnRNP methyltransferase-like 1 (S. cerevisiae) | Hrmt1l1 | CK477970 | 0.612 | GE1274003 |
| Homeo box D10 (predicted) | Hoxd10_predicted | AA818600 | 0.667 | GE1112712 |
| Hook homolog 1 (Drosophila) (predicted) | Hook1_predicted | BF555362 | 0.568 | GE1102812 |
| Huntingtin interacting protein 1 | Hip1 | AW253339 | 1.65 | GE16054 |
| huntingtin-associated protein 1 | Hap1 | NM_177982 | 0.517 | GE1124539 |
| Hypothetical LOC287938 | LOC287938 | BE107630 | 1.432 | GE1289346 |
| Hypothetical LOC292874 (predicted) | RGD1309036_predicted | AA925385 | 3.01 | GE12769 |
| Hypothetical LOC298504 (predicted) | RGD1310174_predicted | AI178784 | 1.487 | GE14563 |
| Hypothetical LOC362710 | LOC362710 | CK652861 | 0.666 | GE1244623 |
| Hypothetical protein LOC680485 | LOC680485 | AA964868 | 1.552 | GE1270188 |
| Hypothetical protein LOC685890 | LOC685890 | CB747249 | 0.52 | GE1261487 |
| inhibin beta-A | Inhba | NM_017128 | 1.576 | GE1246338 |
| Inhibitor of growth family, member 3 | Ing3 | BI296426 | 0.702 | GE1258826 |
| inositol polyphosphate-5-phosphatase D | Inpp5d | NM_019311 | 0.69 | GE22001 |
| Integrin, beta 5 | Itgb5 | CK482040 | 0.708 | GE1264479 |
| interleukin 1 alpha | IL-1 alpha | NM_017019 | 0.7 | GE20278 |
| Interleukin 17 receptor (predicted) | Il17r_predicted | CK478421 | 1.809 | GE1204809 |
| interleukin 22 receptor, alpha 2 | Crf2-s1 | AJ555485 | 0.661 | GE1283339 |
| Junctophilin 2 | Jph2 | AW252152 | 0.618 | GE16028 |
| K+ voltage-gated channel, subfamily S, 1 | Kv9.1 | NM_053954 | 1.44 | GE21208 |
| kalirin, RhoGEF kinase | Duo; Hapip; Kalirin | NM_032062 | 0.644 | GE13496 |
| kelch-like 12 (Drosophila) | C3ip1; MGC93127 | NM_153730 | 0.532 | GE1304749 |
| Kelch-like 22 (Drosophila) (predicted) | Klhl22_predicted | CB548033 | 0.4 | GE1267932 |
| kidney specific organic anion transporter | Slc21a4; OAT-K1; OAT-K2; rOAT-K1; rOAT-K2; rOAT-K3; rOAT-K5; rOAT-K6; rOAT-K7; rOAT-K8; rOAT-K9; rOAT-K11; rOAT-K13; rOAT-K14 | NM_030837 | 1.421 | GE19518 |
| killer cell lectin-like receptor subfamily C, member 1 | rNKG2A | AF021350 | 0.709 | GE1301839 |
| Kinesin family member C1 | Kifc1 | AW253880 | 0.645 | GE16069 |
| Leucine rich repeat neuronal 1 | Lrrn1 | CA505190 | 0.664 | GE1262406 |
| Leucine-rich repeats and immunoglobulin-like domains 1 (predicted) | Lrig1_predicted | CB548206 | 0.691 | GE1266853 |
| Like-glycosyltransferase (predicted) | Large_predicted | BF387136 | 1.449 | GE1261014 |
| lin-7 homolog b (C. elegans) | Veli2; Veli1a | NM_021758 | 1.401 | GE20797 |
| Low density lipoprotein receptor-related protein 11 (predicted) | Lrp11_predicted | BG378716 | 0.579 | GE19242 |
| Low density lipoprotein-related protein 12 (predicted) | Lrp12_predicted | CB544479 | 0.658 | GE1187131 |
| LRP16 protein | Lrp16 | BU758317 | 0.702 | GE1150729 |
| Lymphocyte antigen 6 complex, locus H (predicted) | Ly6h_predicted | AW434178 | 1.452 | GE16116 |
| Lysozyme-like 4 (predicted) | Lyzl4_predicted | BF563386 | 1.506 | GE1213071 |
| MAD homolog 1 (Drosophila) | Madh1 | NM_013130 | 1.453 | GE21379 |
| mannan-binding lectin serine peptidase 1 | Masp3; Masp1/3 | NM_022257 | 0.508 | GE15721 |
| Max dimerization protein 4 (predicted) | Mxd4_predicted | BI281135 | 1.464 | GE17106 |
| microtubule-associated protein 1 light chain 3 alpha | MGC105263 | NM_199500 | 1.467 | GE1175605 |
| Microtubule-associated protein tau | Mapt | AW521314 | 1.574 | GE1194698 |
| Mitochondrial protein, 18 kDa | MGC94604 | AA943734 | 1.418 | GE12796 |
| Mitochondrial ribosomal protein S34 (predicted) | Mrps34_predicted | BI303683 | 1.472 | GE14781 |
| Mitogen activated protein kinase kinase kinase kinase 2 (predicted) | Map4k2_predicted | CB760256 | 0.655 | GE1170412 |
| Mitogen-activated protein kinase kinase kinase 14 (predicted) | Map3k14_predicted | CB585517 | 0.644 | GE1101647 |
| Mitsugumin 29 (predicted) | Mg29_predicted | CB806992 | 0.228 | GE1212995 |
| Muscle and microspikes RAS | Mras | AI407001 | 1.421 | GE15048 |
| muscle, skeletal, receptor tyrosine kinase | Nsk1 | NM_031061 | 0.617 | GE20914 |
| MutS homolog 2 (E. coli) | Msh2 | BF386107 | 0.659 | GE1124351 |
| Myeloid/lymphoid or mixed-lineage leukemia 5 (trithorax homolog, Drosophila) | Mll5 | CB730739 | 0.59 | GE1128740 |
| Myomesin 1 (skelemin) 185kDa | Myom1 | BM383962 | 0.657 | GE1270223 |
| Myosin binding protein C, cardiac (predicted) | Mybpc3_predicted | BI287614 | 0.6 | GE1292401 |
| myosin, light polypeptide 2 | MLC2; Mylpf; Myolc1 | NM_012605 | 3.724 | GE19856 |
| N-acetylgalactosamine 4-sulfate 6-O-sulfotransferase | GalNAc4S6ST | BF545362 | 0.696 | GE1242956 |
| NADH dehydrogenase (ubiquinone) 1 beta subcomplex 3 (predicted) | Ndufb3_predicted | BF417510 | 1.427 | GE1186325 |
| NADH dehydrogenase (ubiquinone) 1 beta subcomplex, 2 (predicted) | Ndufb2_predicted | BM386606 | 1.851 | GE15461 |
| NADH dehydrogenase (ubiquinone) Fe-S protein 3 (predicted) | Ndufs3_predicted | BG379944 | 1.428 | GE1159927 |
| nephrosis 2 homolog, podocin (human) | podocin; MGC112589 | NM_130828 | 0.526 | GE1196651 |
| Neuronal growth regulator 1 | Negr1 | AW533779 | 3.064 | GE1212294 |
| neuropeptide Y | NPY02; RATNPY; RATNPY02 | NM_012614 | 1.53 | GE19864 |
| Nuclear receptor coactivator 3 | Ncoa3 | BF552560 | 0.662 | GE1247284 |
| Nuclear receptor co-repressor 1 | Ncor1 | BF524021 | 0.464 | GE1143281 |
| nucleobindin 1 | Nucb | NM_053463 | 0.689 | GE22043 |
| Odd Oz/ten-m homolog 2 (Drosophila) | Odz2 | BF409534 | 0.679 | GE1217075 |
| Oncoprotein induced transcript 1 homolog (mouse) (predicted) | Oit1_predicted | BM383221 | 0.552 | GE1183882 |
| Oral cancer overexpressed 1 (predicted) | Oraov1_predicted | CB718662 | 0.698 | GE1223961 |
| oxidized low density lipoprotein (lectin-like) receptor 1 | Olr1; LOX-1; Oldr1 | NM_133306 | 0.71 | GE21240 |
| P55 protein | LOC652956 | AI229529 | 1.516 | GE14687 |
| paired-like homeodomain transcription factor 3 | Pitx3 | NM_019247 | 0.695 | GE1208633 |
| pancreatic lipase | PANLI | NM_013161 | 1.509 | GE20216 |
| PCTAIRE-motif protein kinase 1 | Pctk1 | NM_031077 | 1.452 | GE20919 |
| Peroxin 2 | Pex2 | BF401595 | 1.441 | GE1130036 |
| phosphatidylcholine transfer protein | Pctp | NM_017225 | 1.471 | GE20435 |
| Phosphatidylinositol 4-kinase, catalytic, beta polypeptide | Pik4cb | BF403933 | 1.453 | GE1118538 |
| Phosphodiesterase 10A | Pde10a | CB749279 | 0.654 | GE1248132 |
| Phosphodiesterase 9A | Pde9a | BE114417 | 1.517 | GE1276473 |
| Phosphoinositide-3-kinase, regulatory subunit 4, p150 (predicted) | Pik3r4_predicted | CB806898 | 0.7 | GE1247893 |
| Phospholipase A2, group VI | Pla2g6 | BM382819 | 1.432 | GE1156356 |
| Phospholipase C, beta 4 | Plcb4 | BF401170 | 0.344 | GE1203515 |
| Placentae and embryos oncofetal gene | Pem | BI289519 | 1.64 | GE1256119 |
| Plakophilin 4 (predicted) | Pkp4_predicted | BE119535 | 1.843 | GE1182785 |
| Platelet derived growth factor, B polypeptide | Pdgfb | CK599757 | 0.472 | GE1124702 |
| poly(A) binding protein, cytoplasmic 1 | Pabp; Pabp1; MGC91496 | NM_134353 | 1.494 | GE15731 |
| Polymerase (DNA directed), alpha 2 | Pola2 | BQ209427 | 0.572 | GE1109345 |
| potassium inwardly-rectifying channel, subfamily J, member 5 | MGC93525 | NM_017297 | 0.603 | GE21728 |
| potassium inwardly-rectifying channel, subfamily J, member 6 | Kcnj6 | NM_013192 | 0.694 | GE20238 |
| potassium inwardly-rectifying channel, subfamily J, member 9 | Girk3; Kir3.3 | NM_053834 | 0.472 | GE1245341 |
| Potassium voltage-gated channel, shaker-related subfamily, beta member 1 | Kcnab1 | BF396103 | 0.683 | GE1188737 |
| potassium voltage-gated channel, subfamily H (eag-related), member 1 | Kcnh1 | NM_031742 | 0.119 | GE22257 |
| POU domain, class 3, transcription factor 3 | Pou3f3 | BF386744 | 0.702 | GE1269907 |
| Pre-B-cell leukemia transcription factor 1 (predicted) | Pbx1_predicted | CB581827 | 0.678 | GE1177013 |
| Prickle-like 1 (Drosophila) | Prickle1 | AI137981 | 1.655 | GE1179121 |
| Procollagen, type IV, alpha 3 (Goodpasture antigen) binding protein (predicted) | Col4a3bp_predicted | CF115281 | 1.668 | GE1205418 |
| Procollagen, type VI, alpha 1 (predicted) | Col6a1_predicted | AI598402 | 1.598 | GE15511 |
| Procollagen, type XI, alpha 1 | Col11a1 | AW142047 | 1.587 | GE15798 |
| Progesterone receptor | Pgr | BE106259 | 1.629 | GE1110148 |
| progesterone receptor | Pgr | NM_022847 | 0.692 | GE1107647 |
| progesterone receptor membrane component 1 | MPR; 25Dx; VEMA; 25-Dx | NM_021766 | 0.641 | GE20803 |
| progesterone receptor membrane component 1 | MPR; 25Dx; VEMA; 25-Dx | NM_021766 | 0.556 | GE21461 |
| prominin 2 | Trprp; Promrp; Prom-rp | NM_138857 | 1.768 | GE1293040 |
| Prostaglandin E receptor 3 (subtype EP3) | Ptger3 | CB607058 | 0.493 | GE1128978 |
| Protein disulfide isomerase associated 4 | Pdia4 | AI237120 | 0.705 | GE1172620 |
| Protein inhibitor of activated STAT, 4 | Pias4 | BE108748 | 1.533 | GE17471 |
| Protein kinase N3 | Pkn3 | BF282185 | 0.289 | GE18080 |
| Protein kinase, AMP-activated, gamma 2 non-catalytic subunit | Prkag2 | BE110973 | 1.607 | GE16265 |
| protein phosphatase 1, regulatory (inhibitor) subunit 12A | M110; MBSP; Mypt1 | NM_053890 | 1.59 | GE21994 |
| Protein phosphatase 2 (formerly 2A), regulatory subunit B (PR 52), beta isoform | Ppp2r2b | AW529564 | 1.566 | GE1144404 |
| protein tyrosine phosphatase, non-receptor type 2 | Ptpn2 | NM_053990 | 1.89 | GE22143 |
| Protein tyrosine phosphatase, receptor type, R | Ptprr | BF410445 | 0.436 | GE1306025 |
| Protocadherin gamma subfamily C, 3 | Pcdhgc3 | CB796773 | 0.698 | GE1237378 |
| Pseudouridylate synthase 7 homolog (S. cerevisiae) (predicted) | Pus7_predicted | BM390168 | 2.441 | GE1172168 |
| Putative homeodomain transcription factor 1 | Phtf1 | AI137699 | 4.445 | GE1290449 |
| putative pheromone receptor (Go-VN5) | LOC286914 | NM_173130 | 0.663 | GE13181 |
| putative pheromone receptor (Go-VN6) | LOC286982 | NM_173316 | 0.158 | GE13182 |
| Putative regulation protein GS3 | Gs3 | AI555706 | 2.019 | GE1283155 |
| Pyroglutamyl-peptidase I | Pgpep1 | BG377859 | 1.464 | GE1272049 |
| queuine tRNA-ribosyltransferase 1 | Tgt; Tgut | NM_022250 | 1.453 | GE1187376 |
| RAB12, member RAS oncogene family | Rab12 | CK603156 | 0.361 | GE19754 |
| RAB3A interacting protein | Rab3ip | BF411674 | 1.448 | GE18626 |
| RAS guanyl releasing protein 4 | Rasgrp4 | NM_130824 | 0.659 | GE1166304 |
| Ras homolog gene family, member C (predicted) | Rhoc_predicted | AA891940 | 1.627 | GE12629 |
| Ras homolog gene family, member G | Rhog | BE118414 | 1.413 | GE17940 |
| Ras-related GTP binding B | Ragb; MGC105357 | NM_053972 | 2.278 | GE21163 |
| Ratsg2 | Ratsg2 | CA340492 | 0.68 | GE1216950 |
| Regulating synaptic membrane exocytosis 1 | Rims1 | BF400263 | 0.66 | GE1288765 |
| Regulator of G-protein signaling 7 | Rgs7 | BF402644 | 1.468 | GE1305032 |
| Retina zinc finger homeodomain protein | ZFH | NM_199492 | 1.485 | GE1121301 |
| retinal degeneration, slow | Prph2; RSRDS | NM_013021 | 0.437 | GE20121 |
| REX1, RNA exonuclease 1 homolog (S. cerevisiae) | Rexo1 | BE113060 | 1.727 | GE17753 |
| RGD1559909 (predicted) | RGD1559909_predicted | BG373356 | 1.445 | GE1254896 |
| RGD1565616 (predicted) | RGD1565616_predicted | BF405294 | 0.613 | GE1135173 |
| Rho-associated coiled-coil forming kinase 2 | ROK | NM_013022 | 1.449 | GE20122 |
| Ribosomal protein L27a (predicted) | Rpl27a_predicted | AA900726 | 1.529 | GE1203933 |
| Ribosomal protein L8 | Rpl8 | CA509313 | 0.629 | GE1303265 |
| Ring finger protein 1 | Ring1 | AA944239 | 1.588 | GE1305362 |
| RNA binding motif protein 14 | Rbm14 | BF555455 | 0.705 | GE1136992 |
| RNA binding motif protein, X chromosome retrogene (predicted) | Rbmxrt_predicted | CD372755 | 0.713 | GE1161044 |
| Roundabout homolog 1 (Drosophila) | Robo1 | CB739433 | 2.617 | GE1114779 |
| roundabout homolog 4 (Drosophila) | Robo4 | NM_181375 | 0.695 | GE1281541 |
| RT1 class Ib, locus Aw2 | RT1-Aw2 | CF111110 | 0.714 | GE1265065 |
| RT1 class Ib, locus Aw2 | RT1-Aw2 | AI012250 | 0.593 | GE13726 |
| RT1 class II, locus Da | RT1-u; RT1-Daa; RT1-Dab; RT1-Dac; RT1-Dad; RT1-Daf; RT1-Dah; RT1-Dak; RT1-Dal; RT1-Dam; RT1-Dan; MGC112637 | Y00480 | 0.594 | GE21186 |
| sec1 family domain containing 1 | Sly1; RA410; rSly1 | NM_019364 | 0.447 | GE20686 |
| Sel1 (suppressor of lin-12) 1 homolog (C. elegans) | Sel1l | NM_177933 | 1.738 | GE19385 |
| Sema domain, transmembrane domain (TM), and cytoplasmic domain, (semaphorin) 6A (predicted) | Sema6a_predicted | CB605722 | 1.484 | GE1102732 |
| Sema domain, transmembrane domain (TM), and cytoplasmic domain, (semaphorin) 6C | Sema6c | CB738424 | 0.514 | GE1136432 |
| Ser/Thr-like protein kinase lyk4 | Lyk4 | BF420075 | 1.496 | GE1157247 |
| Serologically defined colon cancer antigen 1 | Sdccag1 | BF391855 | 0.558 | GE1110576 |
| SERTA domain containing 1 | MGC72577 | BC061808 | 1.456 | GE12856 |
| SET and MYND domain containing 5 (predicted) | Smyd5_predicted | CB728445 | 0.645 | GE1200696 |
| SH3 domain binding protein CR16 | Cr16 | NM_147211 | 0.627 | GE20895 |
| SH3-domain GRB2-like 1 | SH3P8 | NM_031239 | 1.596 | GE1170336 |
| Sh3kbp1 binding protein 1 (predicted) | Shkbp1_predicted | BF414961 | 1.422 | GE1278422 |
| Shultzomica03302 Rat lung airway and parenchyma cDNA libraries Rattus norvegicus cDNA clone Contig2910 5', mRNA sequence. |  | CF110051 | 0.71 | GE1227809 |
| Sidekick homolog 2 (chicken) (predicted) | Sdk2_predicted | CB727215 | 0.615 | GE1105673 |
| sideroflexin 5 | BBG-TCC | NM_153298 | 1.614 | GE15472 |
| Signal transducing adaptor molecule (SH3 domain and ITAM motif) 2 | Stam2 | CB806056 | 1.74 | GE1249153 |
| Signal-induced proliferation-associated 1 like 1 | Sipa1l1 | BE109893 | 1.416 | GE1284364 |
| Signal-induced proliferation-associated 1 like 1 | Sipa1l1 | BF416623 | 0.645 | GE1169078 |
| Similar to 1700123O20Rik protein (predicted) | RGD1308430_predicted | BQ207968 | 0.71 | GE1277747 |
| Similar to 4930566A11Rik protein (predicted) | RGD1306674_predicted | BE118092 | 2.556 | GE1211104 |
| Similar to A830059I20Rik protein (predicted) | RGD1564695_predicted | BF414617 | 2.104 | GE1129617 |
| Similar to actin-binding LIM protein 1 long isoform (predicted) | RGD1565768_predicted | BF563597 | 0.696 | GE1237350 |
| Similar to adipocyte-specific protein 4 | RGD1308813 | BE116554 | 1.55 | GE1129748 |
| Similar to beta-catenin-interacting protein ICAT | LOC503000 | BF551361 | 1.41 | GE19228 |
| Similar to C330016O16Rik protein | RGD1304861 | CB794424 | 0.708 | GE1185986 |
| Similar to Calponin-2 (Calponin H2, smooth muscle) (Neutral calponin) | LOC690976 | AI230762 | 1.487 | GE14737 |
| Similar to cDNA sequence BC004044 | RGD1311980 | AI178361 | 1.544 | GE14545 |
| Similar to CG6878-PA | LOC679572 | BM387684 | 1.422 | GE1221469 |
| Similar to chromosome 1 open reading frame 172 | RGD1303271 | BM385475 | 0.458 | GE1171770 |
| Similar to chromosome 13 open reading frame 3 (predicted) | RGD1307201_predicted | CB745518 | 0.611 | GE1223934 |
| Similar to Coatomer gamma-2 subunit (Gamma-2 coat protein) (Gamma-2 COP) (predicted) | RGD1566215_predicted | BF563307 | 0.687 | GE1102163 |
| Similar to dehydrogenase/reductase (SDR family) member 10 | LOC691018 | BF543501 | 0.541 | GE1143489 |
| Similar to Delta-interacting protein A (Hepatitis delta antigen interacting protein A) (predicted) | RGD1565319_predicted | BQ205281 | 1.483 | GE1206638 |
| Similar to DNA segment, Chr 4, ERATO Doi 22, expressed (predicted) | RGD1560286_predicted | BQ206818 | 1.786 | GE1177005 |
| Similar to DnaJ (Hsp40) homolog, subfamily B, member 12 | LOC294513 | BF393399 | 1.445 | GE12398 |
| Similar to doublesex and mab-3 related transcription factor 8.1 isoform a | LOC680068 | CB795726 | 0.465 | GE1130841 |
| Similar to E2f3 protein (predicted) | RGD1561600_predicted | AW251447 | 0.526 | GE1302601 |
| Similar to E430002G05Rik protein (predicted) | RGD1308745_predicted | BQ205135 | 0.696 | GE1210573 |
| Similar to Endoplasmic reticulum mannosyl-oligosaccharide 1,2-alpha-mannosidase (ER alpha-1,2-mannosidase) (predicted) | RGD1563595_predicted | BM385415 | 1.571 | GE17424 |
| Similar to expressed sequence AA407526 isoform a (predicted) | RGD1310774_predicted | BE105991 | 2.696 | GE1145259 |
| Similar to expressed sequence AI317237 (predicted) | RGD1305671_predicted | AI555860 | 1.516 | GE1199326 |
| Similar to Fc receptor-like protein 1 | LOC680665 | BQ782064 | 0.653 | GE1192636 |
| Similar to FLI-LRR associated protein-1 | LOC367314 | AI407982 | 1.402 | GE15095 |
| Similar to forkhead box K1 isoform alpha | LOC679672 | BI289361 | 1.628 | GE1102862 |
| Similar to HCV NS3-transactivated protein 1 (predicted) | RGD1306332_predicted | CA504560 | 1.408 | GE1239667 |
| Similar to HECT type E3 ubiquitin ligase (predicted) | RGD1561038_predicted | BG664527 | 1.434 | GE1210664 |
| Similar to hypothetical protein | LOC361041 | BF542426 | 1.586 | GE19118 |
| Similar to hypothetical protein (predicted) | RGD1305269_predicted | BE097634 | 1.443 | GE1193139 |
| Similar to Hypothetical protein BC014729 | MGC105560 | CK474924 | 0.554 | GE1259651 |
| Similar to hypothetical protein C130032F08 (predicted) | RGD1304910_predicted | AA859631 | 1.457 | GE12568 |
| Similar to hypothetical protein DKFZp434G156 (predicted) | RGD1564459_predicted | BF416630 | 0.63 | GE1123073 |
| Similar to hypothetical protein F730001J03 (predicted) | RGD1311757_predicted | CB807084 | 0.236 | GE1174224 |
| Similar to hypothetical protein FLJ10154 | RGD1310061 | BM387709 | 1.433 | GE1227105 |
| Similar to hypothetical protein FLJ11526 (predicted) | RGD1309585_predicted | BF551390 | 1.52 | GE1118411 |
| Similar to hypothetical protein FLJ13188 (predicted) | RGD1305500_predicted | CK603514 | 3.163 | GE1131676 |
| Similar to hypothetical protein FLJ20604 | RGD1311092 | BF407799 | 2.019 | GE18807 |
| Similar to hypothetical protein FLJ21986 | LOC500046 | BQ207240 | 0.639 | GE1170093 |
| Similar to Hypothetical protein KIAA0373 (predicted) | RGD1311640_predicted | AI556611 | 1.501 | GE1247309 |
| Similar to hypothetical protein MGC33486 (predicted) | RGD1310680_predicted | AI716115 | 1.513 | GE15640 |
| Similar to integral membrane protein 1 (predicted) | RGD1565793_predicted | CB706111 | 0.622 | GE1151453 |
| Similar to Kell protein (predicted) | RGD1565470_predicted | BF549303 | 0.521 | GE1250108 |
| Similar to KIAA0605 gene product (predicted) | RGD1305459_predicted | BE107590 | 0.697 | GE1130034 |
| Similar to KIAA0833 protein | LOC362665 | AI231088 | 1.534 | GE14745 |
| Similar to KIAA1749 protein (predicted) | RGD1304623_predicted | CK474957 | 0.599 | GE1262159 |
| Similar to KIAA1862 protein (predicted) | RGD1306967_predicted | AI008701 | 1.442 | GE13618 |
| Similar to lipoma HMGIC fusion partner-like 3 (predicted) | RGD1559727_predicted | CB607423 | 0.697 | GE1177763 |
| Similar to MAPK-interacting and spindle-stabilizing protein (predicted) | RGD1311455_predicted | AI044759 | 0.71 | GE1242329 |
| Similar to mitogen-activated protein kinase kinase kinase kinase 5 isoform 2 (predicted) | RGD1562028_predicted | AI136330 | 2.258 | GE1132885 |
| Similar to mKIAA1045 protein (predicted) | RGD1559864_predicted | BE119028 | 0.634 | GE1170753 |
| Similar to mKIAA1724 protein (predicted) | RGD1560938_predicted | CK475845 | 0.582 | GE1295713 |
| Similar to Myelin P2 protein | LOC688790 | BG672078 | 0.666 | GE1150782 |
| Similar to myocyte enhancer factor 2C | LOC309957 | AI231450 | 1.873 | GE14761 |
| Similar to myosin heavy chain Myr 8 | LOC680480 | AW915635 | 1.768 | GE1108210 |
| Similar to novel protein (predicted) | RGD1563106_predicted | CK478096 | 0.564 | GE1306665 |
| Similar to PACRG (predicted) | RGD1561027_predicted | BE098419 | 0.0846 | GE13700 |
| Similar to peptide N-glycanase | LOC361014 | CK597752 | 0.677 | GE1197654 |
| Similar to phosphatidylinositol-3-phosphate/phosphatidylinositol 5-kinase, type III isoform 2 | LOC316457 | CK596190 | 0.71 | GE1142272 |
| Similar to Phosphatidylserine decarboxylase proenzyme | LOC681361 | AW529876 | 0.649 | GE1180632 |
| Similar to potassium channel tetramerisation domain containing 15 | LOC499129 | BI300259 | 1.408 | GE17305 |
| Similar to potassium voltage-gated channel, Isk-related family, member 1-like | LOC681190 | AI710064 | 1.508 | GE18048 |
| Similar to Probable phospholipid-transporting ATPase ID (ATPase class I type 8B member 2) | LOC685152 | AW522339 | 0.489 | GE1145931 |
| Similar to pseudouridylate synthase-like 1 | LOC362681 | AW533698 | 1.413 | GE1191040 |
| Similar to RAD54B homolog isoform 1; RAD54, S. cerevisiae, homolog of, B (predicted) | RGD1306507_predicted | BF290076 | 1.884 | GE18389 |
| Similar to Ras GTPase-activating-like protein IQGAP2 (predicted) | RGD1561455_predicted | BQ202025 | 1.497 | GE1209214 |
| Similar to Rho GTPase activating protein 20 (predicted) | RGD1561485_predicted | AW527941 | 0.577 | GE1242510 |
| Similar to Rho guanine nucleotide exchange factor 4 isoform a | LOC301334 | BG381432 | 1.409 | GE1168209 |
| Similar to Ribulose-5-phosphate-3-epimerase | MGC124653 | BF409753 | 1.56 | GE1213722 |
| Similar to RIKEN cDNA 1700012G19 gene (predicted) | RGD1307773_predicted | BF420654 | 1.518 | GE19075 |
| Similar to RIKEN cDNA 1810057C19 | MGC108778 | AI535446 | 1.845 | GE1269361 |
| Similar to RIKEN cDNA 2610200G18 (predicted) | RGD1561205_predicted | CA512493 | 0.557 | GE1201880 |
| Similar to RIKEN cDNA 4930455F23 | RGD1309708 | CK595039 | 0.467 | GE1285997 |
| Similar to RIKEN cDNA 5031400M07 | RGD1307343 | CB800617 | 0.396 | GE1265363 |
| Similar to RIKEN cDNA 5830433M19 | MGC125002 | BF287691 | 1.529 | GE1110831 |
| Similar to RIKEN cDNA 5830457O10 (predicted) | RGD1306894_predicted | H31074 | 0.711 | GE1202358 |
| Similar to RIKEN cDNA 6530401L14 gene | RGD1309107 | BE109109 | 1.595 | GE17521 |
| Similar to RIKEN cDNA 8030451K01 (predicted) | RGD1565414_predicted | CB795709 | 0.493 | GE1227028 |
| Similar to selenoprotein SelM (predicted) | RGD1565037_predicted | BQ211773 | 1.778 | GE19181 |
| Similar to SH3 binding domain protein 5 like | LOC690898 | BQ196398 | 0.46 | GE1155308 |
| Similar to SLIT-ROBO Rho GTPase-activating protein 1 (predicted) | RGD1566260_predicted | AI113165 | 0.667 | GE1272962 |
| Similar to sperm antigen HCMOGT-1 | RGD1309718 | CA504602 | 1.583 | GE1229804 |
| Similar to StAR-related protein 1-4E (predicted) | RGD1561783_predicted | BF549814 | 0.672 | GE1269681 |
| Similar to sterile alpha motif domain containing 12 (predicted) | RGD1561402_predicted | BF418960 | 1.846 | GE1199299 |
| Similar to transcriptional regulating protein 132 (predicted) | RGD1306119_predicted | BF407311 | 1.709 | GE1233824 |
| Similar to tropomyosin 1, embryonic fibroblast - rat | MGC109519 | BI281112 | 0.547 | GE18410 |
| Similar to Tubulin--tyrosine ligase-like protein 11 | LOC689746 | BE108128 | 2.837 | GE1179900 |
| Similar to Zinc finger and SCAN domain containing protein 2 (Zinc finger protein 29) (predicted) | RGD1559747_predicted | BE096201 | 0.636 | GE1129170 |
| Similar to Zinc finger protein 133 | LOC499900 | CK601936 | 0.537 | GE1195714 |
| Similar to zinc finger, CCHC domain containing 3 | LOC690005 | BE115479 | 1.948 | GE1261677 |
| SNAP25-interacting protein | Snip | BF404355 | 1.588 | GE1206582 |
| SNRPN upstream reading frame | Snrpn | NM_130738 | 0.618 | GE1176067 |
| sodium channel, voltage-gated, type IV, beta | Scn4b | AF544988 | 1.627 | GE1192467 |
| sodium channel, voltage-gated, type IV, beta | Scn4b | AF544988 | 1.431 | GE14109 |
| Solute carrier family 14 (urea transporter), member 2 | Slc14a2 | AF230638 | 0.634 | GE13494 |
| Solute carrier family 23 (nucleobase transporters), member 2 | Slc23a2 | BE116526 | 1.425 | GE1106544 |
| Solute carrier family 24 (sodium/potassium/calcium exchanger), member 2 | Slc24a2 | BF400617 | 0.606 | GE1289440 |
| Solute carrier family 30 (zinc transporter), member 3 | Slc30a3 | CK474873 | 0.711 | GE1306471 |
| solute carrier family 35, member E4 | MGC93128 | NM_153316 | 1.503 | GE13436 |
| solute carrier family 4, member 3 | Ae3; Aep3 | NM_017049 | 1.458 | GE20303 |
| Son cell proliferation protein | Son | AW144637 | 1.472 | GE15970 |
| Sortilin-related receptor, L(DLR class) A repeats-containing (predicted) | Sorl1_predicted | BQ208287 | 1.549 | GE1135618 |
| Sortilin-related VPS10 domain containing receptor 2 (predicted) | Sorcs2_predicted | BF403840 | 0.441 | GE1156497 |
| Sp1 transcription factor | Sp1 | CA508480 | 1.706 | GE1223041 |
| Sparc/osteonectin, cwcv and kazal-like domains proteoglycan 2 (predicted) | Spock2_predicted | CK355557 | 0.674 | GE1181653 |
| spectrin beta 3 | Sptbn2 | NM_019167 | 1.494 | GE20563 |
| SplA/ryanodine receptor domain and SOCS box containing 1 (predicted) | Spsb1_predicted | BE349698 | 1.516 | GE18019 |
| SRY-box containing gene 4 (predicted) | Sox4_predicted | CA512057 | 1.412 | GE14102 |
| succinate-CoA ligase, GDP-forming, alpha subunit | Suclg1 | NM_053752 | 0.664 | GE19570 |
| suppression of tumorigenicity 18 | Nzf3; r-MyT3 | NM_153310 | 0.703 | GE21000 |
| synaptic Ras GTPase activating protein 1 homolog (rat) | Syngap | AF058790 | 0.557 | GE21272 |
| Tax1 (human T-cell leukemia virus type I) binding protein 3 | Tax1bp3 | AW144510 | 1.43 | GE15966 |
| TBC1 domain family, member 1 (predicted) | Tbc1d1_predicted | BF405886 | 1.606 | GE1292184 |
| TBC1 domain family, member 20 | Tbc1d20 | BE116161 | 1.418 | GE1152795 |
| testis expressed gene 101 | Tec21 | NM_139037 | 0.662 | GE1163705 |
| Testis expressed gene 261 | Tex261 | BE115923 | 0.712 | GE21352 |
| Testis-specific serine kinase 2 | Tssk2 | BE110023 | 0.293 | GE1188415 |
| Thioredoxin 2 | Txn2 | BI288617 | 0.669 | GE16942 |
| Thioredoxin domain containing 11 (predicted) | Txndc11_predicted | CB801729 | 0.517 | GE1104790 |
| Thyroid hormone receptor associated protein 5 (predicted) | Thrap5_predicted | BG381442 | 1.511 | GE1186469 |
| Trafficking protein, kinesin binding 2 | Trak2 | BG373530 | 1.748 | GE1199567 |
| Transcribed locus | Ak3 | BF408893 | 2.737 | GE18844 |
| Transcribed locus |  | BE120454 | 2.239 | GE1180897 |
| Transcribed locus |  | AA819186 | 2.065 | GE1216426 |
| Transcribed locus |  | AA957047 | 2.064 | GE12944 |
| Transcribed locus |  | BF416018 | 1.953 | GE1219601 |
| Transcribed locus | Slc24a2 | CB581086 | 1.951 | GE1158189 |
| Transcribed locus |  | BF419716 | 1.875 | GE1220071 |
| Transcribed locus |  | CA503774 | 1.864 | GE14651 |
| Transcribed locus |  | BE116825 | 1.839 | GE1138402 |
| Transcribed locus |  | BF403271 | 1.821 | GE1186113 |
| Transcribed locus |  | BE095962 | 1.82 | GE1195401 |
| Transcribed locus |  | AI071563 | 1.749 | GE1234786 |
| Transcribed locus | MGC72630 | BE116939 | 1.722 | GE1145702 |
| Transcribed locus |  | BQ200251 | 1.695 | GE1214362 |
| Transcribed locus |  | BF552829 | 1.683 | GE1167981 |
| Transcribed locus |  | BE120016 | 1.616 | GE17969 |
| Transcribed locus |  | BI275314 | 1.601 | GE13932 |
| Transcribed locus |  | AW916210 | 1.577 | GE16696 |
| Transcribed locus |  | BE117113 | 1.566 | GE14229 |
| Transcribed locus |  | BQ210650 | 1.565 | GE12740 |
| Transcribed locus |  | BF404445 | 1.562 | GE1104918 |
| Transcribed locus |  | CA506897 | 1.544 | GE1217593 |
| Transcribed locus |  | BF401650 | 1.531 | GE1174385 |
| Transcribed locus |  | BI288592 | 1.521 | GE1108396 |
| Transcribed locus |  | AW523282 | 1.518 | GE1227438 |
| Transcribed locus | Timm8b | BI300245 | 1.503 | GE1171870 |
| Transcribed locus |  | BF564013 | 1.491 | GE1129679 |
| Transcribed locus |  | BF389145 | 1.489 | GE18347 |
| Transcribed locus |  | BF413021 | 1.482 | GE1107667 |
| Transcribed locus | Dap4 | AI072218 | 1.481 | GE13847 |
| Transcribed locus |  | AI234810 | 1.48 | GE14876 |
| Transcribed locus |  | AA999082 | 1.473 | GE1120977 |
| Transcribed locus |  | AI717256 | 1.472 | GE1206645 |
| Transcribed locus |  | BF548743 | 1.472 | GE19157 |
| Transcribed locus |  | BM387354 | 1.466 | GE1216534 |
| Transcribed locus |  | BE120675 | 1.462 | GE1233738 |
| Transcribed locus | Rbl2 | BE119501 | 1.458 | GE1208085 |
| Transcribed locus |  | BG371790 | 1.451 | GE1108126 |
| Transcribed locus |  | BE112790 | 1.45 | GE12902 |
| Transcribed locus |  | BI294015 | 1.449 | GE1305006 |
| Transcribed locus |  | AI111413 | 1.448 | GE1101563 |
| Transcribed locus |  | CA505374 | 1.439 | GE1166294 |
| Transcribed locus |  | BE118444 | 1.438 | GE1103635 |
| Transcribed locus |  | BF405110 | 1.434 | GE18744 |
| Transcribed locus |  | AI575096 | 1.432 | GE1245049 |
| Transcribed locus |  | AI071758 | 1.425 | GE1237077 |
| Transcribed locus |  | BM391628 | 1.423 | GE1295381 |
| Transcribed locus |  | BE111126 | 1.422 | GE1177808 |
| Transcribed locus |  | BF405810 | 1.419 | GE1224934 |
| Transcribed locus |  | BG373265 | 1.418 | GE1278307 |
| Transcribed locus | Syncrip | BU760298 | 1.416 | GE17487 |
| Transcribed locus |  | AW525238 | 1.41 | GE1233953 |
| Transcribed locus |  | BF386678 | 1.407 | GE1257582 |
| Transcribed locus |  | CA510283 | 1.403 | GE1225943 |
| Transcribed locus |  | BF285946 | 1.402 | GE18325 |
| Transcribed locus |  | BF394576 | 1.401 | GE1108245 |
| Transcribed locus |  | BF568017 | 0.713 | GE1184602 |
| Transcribed locus |  | BF400238 | 0.711 | GE1240834 |
| Transcribed locus |  | BF413922 | 0.71 | GE1274102 |
| Transcribed locus |  | BF397814 | 0.71 | GE1231030 |
| Transcribed locus |  | BG377517 | 0.709 | GE1250226 |
| Transcribed locus |  | CK477390 | 0.706 | GE1138867 |
| Transcribed locus |  | CB719837 | 0.704 | GE1154190 |
| Transcribed locus |  | BI295939 | 0.704 | GE12945 |
| Transcribed locus |  | BI292017 | 0.702 | GE1237046 |
| Transcribed locus |  | BI283806 | 0.697 | GE14688 |
| Transcribed locus |  | BF524872 | 0.691 | GE19108 |
| Transcribed locus |  | BF403185 | 0.689 | GE1235811 |
| Transcribed locus |  | BM389972 | 0.687 | GE1142251 |
| Transcribed locus |  | BM391716 | 0.684 | GE1266681 |
| Transcribed locus |  | BG371778 | 0.684 | GE1140374 |
| Transcribed locus |  | AW917390 | 0.683 | GE16779 |
| Transcribed locus |  | BE104430 | 0.68 | GE1195863 |
| Transcribed locus |  | BF386187 | 0.678 | GE1138919 |
| Transcribed locus |  | BQ211223 | 0.677 | GE1242132 |
| Transcribed locus |  | BF418034 | 0.669 | GE1238886 |
| Transcribed locus |  | BF393546 | 0.664 | GE1222096 |
| Transcribed locus |  | CK602984 | 0.663 | GE1290424 |
| Transcribed locus | Slc4a4 | BF392259 | 0.659 | GE1252475 |
| Transcribed locus |  | BF283033 | 0.658 | GE1104989 |
| Transcribed locus |  | BF392479 | 0.654 | GE1299897 |
| Transcribed locus |  | BF398933 | 0.652 | GE1160132 |
| Transcribed locus |  | AI058421 | 0.65 | GE1142618 |
| Transcribed locus | Hif1a | BF415930 | 0.645 | GE1101411 |
| Transcribed locus |  | AI029272 | 0.63 | GE1175267 |
| Transcribed locus |  | BF524742 | 0.627 | GE1265954 |
| Transcribed locus |  | BF416303 | 0.621 | GE1178021 |
| Transcribed locus |  | CK366578 | 0.621 | GE1127107 |
| Transcribed locus |  | AI145548 | 0.619 | GE1109341 |
| Transcribed locus | Adcy5 | BF407938 | 0.608 | GE1250850 |
| Transcribed locus |  | BE119817 | 0.608 | GE1119740 |
| Transcribed locus |  | CK366904 | 0.606 | GE1178622 |
| Transcribed locus | Plm | AI231802 | 0.604 | GE14783 |
| Transcribed locus |  | BF553038 | 0.603 | GE1140770 |
| Transcribed locus |  | BM391697 | 0.571 | GE1259526 |
| Transcribed locus |  | AI233214 | 0.555 | GE1291721 |
| Transcribed locus |  | CB771266 | 0.549 | GE1251641 |
| Transcribed locus |  | BF394813 | 0.543 | GE1193136 |
| Transcribed locus |  | AW535975 | 0.538 | GE1199689 |
| Transcribed locus |  | BF563097 | 0.526 | GE1138376 |
| Transcribed locus |  | BE115804 | 0.526 | GE12330 |
| Transcribed locus |  | AI071669 | 0.514 | GE1254344 |
| Transcribed locus | RGD1311863 | BE119823 | 0.514 | GE1164752 |
| Transcribed locus |  | BF397708 | 0.509 | GE1301768 |
| Transcribed locus |  | BF564197 | 0.506 | GE1139142 |
| Transcribed locus |  | BE117485 | 0.493 | GE1194566 |
| Transcribed locus |  | BM383349 | 0.478 | GE1238211 |
| Transcribed locus |  | AI763575 | 0.461 | GE12669 |
| Transcribed locus |  | BM389481 | 0.443 | GE1195112 |
| Transcribed locus |  | AI113112 | 0.421 | GE1201658 |
| Transcribed locus |  | BF396126 | 0.374 | GE1275672 |
| Transcribed locus |  | AI501311 | 0.347 | GE1216548 |
| Transcribed locus |  | BM390996 | 0.311 | GE1287953 |
| Transcribed locus |  | BF285379 | 0.307 | GE1133757 |
| Transcribed locus |  | BI300295 | 0.28 | GE1103458 |
| Transcribed locus |  | BF413839 | 0.263 | GE1187669 |
| Transcribed locus |  | BG371456 | 0.26 | GE1274830 |
| Transcribed locus | Prkce | H31660 | 0.247 | GE1185997 |
| Transcribed locus |  | BE100769 | 0.158 | GE1176284 |
| Transcribed locus, moderately similar to XP_529632.1 PREDICTED: hypothetical protein XP_529632 [Pan troglodytes] | Porf1 | BM384356 | 1.606 | GE1178712 |
| Transcribed locus, moderately similar to XP_546425.2 PREDICTED: similar to roundabout, axon guidance receptor, homolog 3 [Canis familiaris] |  | BF390065 | 1.906 | GE1287744 |
| Transcribed locus, moderately similar to XP_576460.1 PREDICTED: similar to hypothetical protein PB402898.00.0 [Rattus norvegicus] |  | BI281220 | 0.102 | GE1194109 |
| Transcribed locus, moderately similar to XP_577201.1 PREDICTED: similar to envelope protein [Rattus norvegicus] |  | AA866233 | 0.336 | GE1103079 |
| Transcribed locus, moderately similar to XP_580187.1 PREDICTED: hypothetical protein XP_580187 [Rattus norvegicus] |  | AA998232 | 1.781 | GE1196255 |
| Transcribed locus, strongly similar to NP_003890.1 Rho guanine nucleotide exchange factor 7 isoform a [Homo sapiens] | Pak3bp | BE104891 | 1.408 | GE17327 |
| Transcribed locus, strongly similar to NP_005651.1 solute carrier family 35 member A2 isoform a [Homo sapiens] |  | BM390740 | 1.402 | GE1158064 |
| Transcribed locus, strongly similar to NP_071949.1 ribosomal protein L36 [Rattus norvegicus] | Rpl36 | BF524750 | 0.552 | GE1108316 |
| Transcribed locus, strongly similar to NP_071949.1 ribosomal protein L36 [Rattus norvegicus] |  | CB761118 | 0.176 | GE1300775 |
| Transcribed locus, strongly similar to NP_775545.1 ubiquitin carboxyl-terminal hydrolase CYLD [Mus musculus] |  | BF391248 | 0.6 | GE1202629 |
| Transcribed locus, strongly similar to NP_942122.2 G protein-coupled receptor 133 [Homo sapiens] |  | CB556941 | 0.702 | GE1280121 |
| Transcribed locus, strongly similar to XP_214069.3 PREDICTED: similar to PES1 protein [Rattus norvegicus] |  | AW919092 | 0.575 | GE16981 |
| Transcribed locus, strongly similar to XP_214253.3 PREDICTED: similar to muscleblind-like 2 isoform 1 [Rattus norvegicus] |  | BF399362 | 1.409 | GE1142425 |
| Transcribed locus, strongly similar to XP_214662.3 PREDICTED: similar to hypothetical protein BC004337 [Rattus norvegicus] |  | AA800507 | 0.565 | GE12296 |
| Transcribed locus, strongly similar to XP_215875.3 PREDICTED: similar to Cystatin-related protein 2 precursor (Prostatic 22 kDa glycoprotein P22K15) [Rattus norvegicus] |  | BM384539 | 0.497 | GE1198460 |
| Transcribed locus, strongly similar to XP_217223.2 PREDICTED: similar to solute carrier family 9 (sodium/hydrogen exchanger), isoform 9 [Rattus norvegicus] |  | BQ195654 | 0.46 | GE1187352 |
| Transcribed locus, strongly similar to XP_218502.3 PREDICTED: similar to ARX [Rattus norvegicus] |  | AW524750 | 1.455 | GE1207405 |
| Transcribed locus, strongly similar to XP_221232.3 PREDICTED: similar to 5730455P16Rik protein [Rattus norvegicus] |  | BG378024 | 1.403 | GE1119782 |
| Transcribed locus, strongly similar to XP_222491.3 PREDICTED: cadherin 7, type 2 (predicted) [Rattus norvegicus] | Cdh7 | BF409052 | 1.525 | GE1274494 |
| Transcribed locus, strongly similar to XP_226249.3 PREDICTED: similar to amyloid beta precursor protein binding protein 1 [Rattus norvegicus] |  | AW534448 | 0.563 | GE13971 |
| Transcribed locus, strongly similar to XP_226471.2 PREDICTED: similar to Mtr3 (mRNA transport regulator 3)-homolog [Rattus norvegicus] |  | AW251819 | 0.568 | GE1227353 |
| Transcribed locus, strongly similar to XP_228025.3 PREDICTED: similar to ICBP90 binding protein 1 [Rattus norvegicus] |  | CB799616 | 0.694 | GE1126396 |
| Transcribed locus, strongly similar to XP_229108.3 PREDICTED: similar to zinc finger, DHHC domain containing 9 [Rattus norvegicus] |  | BM388260 | 1.401 | GE1199912 |
| Transcribed locus, strongly similar to XP_231594.3 PREDICTED: similar to hypothetical protein FLJ32786 [Rattus norvegicus] |  | BF392798 | 1.524 | GE18691 |
| Transcribed locus, strongly similar to XP_341580.2 PREDICTED: similar to Niemann Pick type C1 [Rattus norvegicus] |  | CK364910 | 0.701 | GE1227488 |
| Transcribed locus, strongly similar to XP_341868.2 PREDICTED: similar to ubiquitin protein ligase E3A isoform 3 [Rattus norvegicus] |  | AA859604 | 1.401 | GE1103948 |
| Transcribed locus, strongly similar to XP_343328.1 PREDICTED: desert hedgehog homolog [Rattus norvegicus] | Dhh | AW529714 | 0.704 | GE1179703 |
| Transcribed locus, strongly similar to XP_573673.1 PREDICTED: similar to 5330440M15Rik protein [Rattus norvegicus] |  | AA955234 | 0.66 | GE1275228 |
| Transcribed locus, strongly similar to XP_573981.1 PREDICTED: similar to heterogeneous nuclear ribonucleoprotein A0 [Rattus norvegicus] |  | BF397834 | 1.517 | GE18569 |
| Transcribed locus, strongly similar to XP_579894.1 PREDICTED: hypothetical protein XP_579894 [Rattus norvegicus] |  | BG376805 | 0.531 | GE1287970 |
| Transcribed locus, strongly similar to XP_579947.1 PREDICTED: hypothetical protein XP_579947 [Rattus norvegicus] | Chrm1 | AI547415 | 0.53 | GE1242826 |
| Transcribed locus, strongly similar to XP_580013.1 PREDICTED: hypothetical protein XP_580013 [Rattus norvegicus] |  | AW920546 | 0.685 | GE1136098 |
| Transcribed locus, strongly similar to XP_580127.1 PREDICTED: hypothetical protein XP_580127 [Rattus norvegicus] |  | CB719701 | 0.706 | GE1205932 |
| Transcribed locus, strongly similar to XP_580137.1 PREDICTED: hypothetical protein XP_580137 [Rattus norvegicus] |  | BM384213 | 0.391 | GE14933 |
| Transcribed locus, strongly similar to XP_580223.1 PREDICTED: hypothetical protein XP_580223 [Rattus norvegicus] |  | AW534182 | 1.425 | GE1105505 |
| Transcribed locus, weakly similar to NP_001003141.1 protein tyrosine kinase fer [Canis familiaris] |  | BE117327 | 0.666 | GE16643 |
| Transcribed locus, weakly similar to XP_422276.1 PREDICTED: hypothetical protein XP_422276 [Gallus gallus] |  | BQ211274 | 1.951 | GE17558 |
| Transcribed locus, weakly similar to XP_512769.1 PREDICTED: hypothetical protein XP_512769 [Pan troglodytes] | MGC72567 | AW526656 | 0.195 | GE1290543 |
| Transcribed locus, weakly similar to XP_529735.1 PREDICTED: hypothetical protein XP_529735 [Pan troglodytes] |  | CB782037 | 0.696 | GE1244031 |
| Transcribed locus, weakly similar to XP_576810.1 PREDICTED: similar to RIKEN cDNA 4930555G01 [Rattus norvegicus] |  | CB606098 | 1.496 | GE1219990 |
| Transcribed locus, weakly similar to XP_580018.1 PREDICTED: hypothetical protein XP_580018 [Rattus norvegicus] |  | BI278180 | 1.979 | GE1248493 |
| Transcribed locus, weakly similar to XP_853549.1 PREDICTED: similar to GTPase, IMAP family member 8 [Canis familiaris] |  | BQ192367 | 0.648 | GE1240209 |
| Transcription elongation regulator 1 (CA150) (predicted) | Tcerg1_predicted | BF552916 | 1.724 | GE19236 |
| Transcription factor 20 (mapped) | Tcf20_mapped | BE120354 | 0.665 | GE17976 |
| Transcription factor 21 | Tcf21 | BI295017 | 0.685 | GE1286063 |
| transcription factor 4 | Tcf4 | NM_053369 | 1.551 | GE20851 |
| Transient receptor potential cation channel, subfamily C, member 4 associated protein | Trpc4ap | BM390154 | 1.458 | GE1181121 |
| Transmembrane protein 103 (predicted) | Tmem103_predicted | BG381479 | 1.405 | GE1265209 |
| Transmembrane protein with EGF-like and two follistatin-like domains 1 | Tmeff1 | BI295864 | 1.623 | GE1203147 |
| Treacher Collins Franceschetti syndrome 1, homolog (predicted) | Tcof1_predicted | AA996517 | 0.601 | GE12972 |
| tripartite motif protein 17 | Rnf16 | NM_022798 | 1.745 | GE1157559 |
| Tropomodulin 3 | Tmod3 | BF566487 | 0.536 | GE1184123 |
| tumor necrosis factor, alpha-induced protein 1 (endothelial) | Edp1 | NM_182950 | 0.674 | GE1129236 |
| Tumor protein p53 inducible protein 13 (predicted) | Trp53i13_predicted | H35532 | 1.634 | GE14715 |
| Tweety homolog 1 (Drosophila) (predicted) | Ttyh1_predicted | AI412746 | 1.915 | GE15374 |
| Tweety homolog 3 (Drosophila) (predicted) | Ttyh3_predicted | CA512117 | 0.616 | GE1213706 |
| Type I keratin KA15 | Ka15 | AW529723 | 0.656 | GE16317 |
| tyrosine 3-monooxygenase/tryptophan 5-monooxygenase activation protein, eta polypeptide | 14-3-3e; MGC93547 | NM_013052 | 1.448 | GE20141 |
| tyrosine aminotransferase | Tat | NM_012668 | 0.713 | GE19890 |
| Ubiquitin specific peptidase 49 (predicted) | Usp49_predicted | AA998397 | 0.441 | GE1116010 |
| ubiquitin specific protease 48 | Usp31; synUSP | NM_198785 | 0.707 | GE16834 |
| Ubiquitination factor E4B, UFD2 homolog (S. cerevisiae) (predicted) | Ube4b_predicted | BG375372 | 1.441 | GE1171881 |
| Ubiquitin-conjugating enzyme E2E 2 (UBC4/5 homolog, yeast) | Ube2e2 | CB793719 | 0.711 | GE1298244 |
| UI-R-AB1-yr-h-09-0-UI.s1 UI-R-AB1 Rattus norvegicus cDNA clone UI-R-AB1-yr-h-09-0-UI 3', mRNA sequence. |  | AI703615 | 0.561 | GE1156573 |
| UI-R-BJ0-aes-d-07-0-UI.s1 UI-R-BJ0 Rattus norvegicus cDNA clone UI-R-BJ0-aes-d-07-0-UI 3', mRNA sequence. |  | AW253709 | 0.516 | GE1160750 |
| UI-R-BO0-agp-h-01-0-UI.r1 UI-R-BO0 Rattus norvegicus cDNA clone UI-R-BO0-agp-h-01-0-UI 5', mRNA sequence. |  | BF567545 | 0.309 | GE1255329 |
| UI-R-BO0-agu-h-09-0-UI.r1 UI-R-BO0 Rattus norvegicus cDNA clone UI-R-BO0-agu-h-09-0-UI 5', mRNA sequence. |  | BF567678 | 0.19 | GE1187980 |
| UI-R-BO1-ajq-f-07-0-UI.r1 UI-R-BO1 Rattus norvegicus cDNA clone UI-R-BO1-ajq-f-07-0-UI 5', mRNA sequence. |  | BF565885 | 0.661 | GE1120122 |
| UI-R-BO1-ajv-e-05-0-UI.s1 UI-R-BO1 Rattus norvegicus cDNA clone UI-R-BO1-ajv-e-05-0-UI 3', mRNA sequence. |  | AW527545 | 0.6 | GE1234291 |
| UI-R-BO1-apo-e-02-0-UI.s1 UI-R-BO1 Rattus norvegicus cDNA clone UI-R-BO1-apo-e-02-0-UI 3', mRNA sequence. |  | BE095642 | 0.693 | GE1100215 |
| UI-R-BT0-pl-f-02-0-UI.r1 UI-R-BT0 Rattus norvegicus cDNA clone UI-R-BT0-pl-f-02-0-UI 5', mRNA sequence. |  | BF553686 | 0.117 | GE1121913 |
| UI-R-BU0-amv-f-01-0-UI.r1 UI-R-BU0 Rattus norvegicus cDNA clone UI-R-BU0-amv-f-01-0-UI 5', mRNA sequence. |  | BF564699 | 0.689 | GE1104759 |
| UI-R-C0-hl-g-10-0-UI.r1 UI-R-C0 Rattus norvegicus cDNA clone UI-R-C0-hl-g-10-0-UI 5', mRNA sequence. |  | BF561292 | 0.67 | GE1299848 |
| UI-R-C0-it-f-02-0-UI.s1 UI-R-C0 Rattus norvegicus cDNA clone UI-R-C0-it-f-02-0-UI 3', mRNA sequence. |  | AI030258 | 0.457 | GE1216973 |
| UI-R-C0-jp-b-11-0-UI.s1 UI-R-C0 Rattus norvegicus cDNA clone UI-R-C0-jp-b-11-0-UI 3', mRNA sequence. |  | AI031031 | 1.597 | GE1207952 |
| UI-R-C1-kk-g-10-0-UI.s1 UI-R-C1 Rattus norvegicus cDNA clone UI-R-C1-kk-g-10-0-UI 3', mRNA sequence. |  | AI045178 | 0.641 | GE1134313 |
| UI-R-C2-nf-c-06-0-UI.r1 UI-R-C2 Rattus norvegicus cDNA clone UI-R-C2-nf-c-06-0-UI 5', mRNA sequence. |  | BF552988 | 0.359 | GE1250521 |
| UI-R-C2p-qz-f-05-0-UI.r1 UI-R-C2p Rattus norvegicus cDNA clone UI-R-C2p-qz-f-05-0-UI 5', mRNA sequence. |  | BF522996 | 1.553 | GE1280874 |
| UI-R-CA0-axk-c-12-0-UI.s1 UI-R-CA0 Rattus norvegicus cDNA clone UI-R-CA0-axk-c-12-0-UI 3', mRNA sequence. |  | BE110207 | 0.309 | GE17611 |
| UI-R-CA1-bls-c-01-0-UI.s1 UI-R-CA1 Rattus norvegicus cDNA clone UI-R-CA1-bls-c-01-0-UI 3', mRNA sequence. |  | BF409738 | 1.74 | GE1198667 |
| UI-R-E1-fe-b-07-0-UI.s1 UI-R-E1 Rattus norvegicus cDNA clone UI-R-E1-fe-b-07-0-UI 3', mRNA sequence. |  | AA956031 | 0.544 | GE1116227 |
| UI-R-FJ0-cpw-k-11-0-UI.r1 UI-R-FJ0 Rattus norvegicus cDNA clone UI-R-FJ0-cpw-k-11-0-UI 5', mRNA sequence. |  | CA511222 | 0.707 | GE1188394 |
| UI-R-FS0-crs-p-08-0-UI.s1 NCI_CGAP_FS0 Rattus norvegicus cDNA clone IMAGE:7358938 3', mRNA sequence. |  | CB327498 | 2.054 | GE1225035 |
| UI-R-GO0-csi-g-20-0-UI.r1 UI-R-GO0 Rattus norvegicus cDNA clone UI-R-GO0-csi-g-20-0-UI 5', mRNA sequence. |  | CD373148 | 0.594 | GE1130589 |
| unc-13 homolog B (C. elegans) | Unc13h2; Munc13-2 | NM_022862 | 0.55 | GE1122413 |
| utrophin | Utrn | NM_013070 | 0.687 | GE1255350 |
| Vacuolar protein sorting 4a (yeast) | Vps4a | AA894099 | 1.419 | GE12718 |
| Vasodilator-stimulated phosphoprotein (predicted) | Vasp_predicted | CA333858 | 0.628 | GE1177827 |
| Vesicle transport through interaction with t-SNAREs homolog 1A (yeast) | Vti1a | CB730537 | 1.537 | GE1224645 |
| vitronectin | Vn; Aa1018; MGC124961 | NM_019156 | 0.681 | GE20554 |
| WD repeat domain 59 | Wdr59 | BQ201885 | 1.432 | GE1297800 |
| WD repeats and SOF domain containing 1 (predicted) | Wdsof1_predicted | BF286579 | 0.691 | GE1279423 |
| WW domain containing E3 ubiquitin protein ligase 2 (predicted) | Wwp2_predicted | BF551945 | 0.681 | GE1150970 |
| YTH domain family 2 (predicted) | Ythdf2_predicted | AF473850 | 1.42 | GE1157433 |
| Zinc finger and BTB domain containing 10 | Zbtb10 | BI287941 | 1.811 | GE1236171 |
| Zinc finger homeobox 2 | Zfhx2 | BE118113 | 1.436 | GE15661 |
| Zinc finger protein 612 (predicted) | Zfp612_predicted | BF410174 | 0.0847 | GE1208322 |
| Zinc finger protein 74 | Zfp74 | AW535772 | 0.697 | GE1240622 |
| Zinc finger protein 84 (predicted) | Zfp84_predicted | AI598638 | 0.665 | GE1281504 |
| ZW10 homolog, centromere/kinetochore protein (Drosophila) | Zw10 | AI230413 | 0.32 | GE14717 |

**mPFC**

Both (Naïve vs. 1-day and Naïve vs. 100-day)

|  | **Naïve v 1-day** | **Naïve v 10-days** |  |
| --- | --- | --- | --- |
| **Gene** | **Fold Change** | **Fold Change** | **Probe ID** |
| activity regulated cytoskeletal-associated protein | 3.109 | 1.932 | GE20683 |
| AGENCOURT_17182824 NIH_MGC_233 Rattus norvegicus cDNA clone IMAGE:7101024 5', mRNA sequence. | 0.696 | 0.639 | GE1215330 |
| AGENCOURT_17578897 NIH_MGC_232 Rattus norvegicus cDNA clone IMAGE:7124785 5', mRNA sequence. | 0.528 | 0.541 | GE1299668 |
| AGENCOURT_17638475 NIH_MGC_235 Rattus norvegicus cDNA clone IMAGE:7107569 5', mRNA sequence. | 0.524 | 0.621 | GE1151175 |
| aldo-keto reductase family 1, member D1 | 0.513 | 0.705 | GE1253447 |
| AMGNNUC:NRDG1-00003-G3-A nrdg1 (10855) Rattus norvegicus cDNA clone nrdg1-00003-g3 5', mRNA sequence. | 1.598 | 1.447 | GE1169415 |
| AMGNNUC:NRHY5-00234-A11-A W Rat hypothalamus (10471) Rattus norvegicus cDNA clone nrhy5-00234-a11 5', mRNA sequence. | 0.543 | 0.651 | GE1192718 |
| Ankyrin repeat and MYND domain containing 2 (predicted) | 1.601 | 1.788 | GE1251814 |
| Ankyrin repeat and SOCS box-containing protein 11 (predicted) | 0.41 | 0.697 | GE18260 |
| Anterior pharynx defective 1a homolog (C. elegans) | 1.503 | 1.752 | GE16545 |
| Arsenic (+3 oxidation state) methyltransferase | 0.604 | 0.599 | GE1203640 |
| ATP synthase, H+ transporting, mitochondrial F1 complex, epsilon subunit | 1.6 | 1.716 | GE1202898 |
| BMP and activin membrane-bound inhibitor, homolog (Xenopus laevis) | 1.955 | 2.089 | GE18823 |
| brain abundant, membrane attached signal protein 1 | 1.547 | 1.447 | GE1197450 |
| BTB (POZ) domain containing 14A | 0.6 | 0.623 | GE1225273 |
| C1q and tumor necrosis factor related protein 4 (predicted) | 1.586 | 1.513 | GE16897 |
| Cadherin 16 | 0.655 | 0.624 | GE12623 |
| calcium channel, voltage-dependent, beta 3 subunit | 1.451 | 1.483 | GE19992 |
| calcium/calmodulin-dependent protein kinase II, beta | 1.496 | 1.906 | GE20786 |
| Caldesmon 1 | 0.5 | 0.679 | GE1111716 |
| carcinoembryonic antigen-related cell adhesion molecule 3 | 0.57 | 0.581 | GE19911 |
| CASK-interacting protein CIP98 | 1.546 | 1.409 | GE16327 |
| CD47 antigen (Rh-related antigen, integrin-associated signal transducer) | 2.246 | 2.954 | GE21321 |
| CDNA BC060737 (predicted) | 0.413 | 0.29 | GE1161677 |
| CDNA clone IMAGE:7303896 | 1.424 | 1.414 | GE15391 |
| CDNA clone IMAGE:7324860 | 1.521 | 1.431 | GE1271845 |
| CDNA clone IMAGE:7375734 | 2.28 | 2.069 | GE1124365 |
| CDNA clone MGC:95041 IMAGE:7123253 | 1.472 | 1.506 | GE19018 |
| Centaurin, gamma 1 | 1.559 | 1.574 | GE16248 |
| chemokine binding protein 2 | 0.463 | 0.581 | GE22070 |
| chondroitin sulfate proteoglycan 3 | 1.527 | 1.455 | GE19775 |
| Coiled-coil domain containing 37 (predicted) | 0.713 | 0.681 | GE1103230 |
| Coilin | 0.615 | 0.688 | GE1252497 |
| CXXC finger 5 | 1.527 | 1.475 | GE15397 |
| Cyclin D3 | 1.87 | 1.615 | GE1112083 |
| Cytochrome c oxidase, subunit VIa, polypeptide 1 | 1.483 | 1.426 | GE1269464 |
| Cytochrome c oxidase, subunit VIIIa | 1.588 | 1.52 | GE1137837 |
| cytochrome P450, subfamily 3A, polypeptide 3 | 0.528 | 0.583 | GE21745 |
| DEAD (Asp-Glu-Ala-Asp) box polypeptide 50 | 1.689 | 1.469 | GE1223116 |
| decorin | 0.652 | 0.61 | GE22148 |
| dopamine receptor D4 | 0.581 | 0.653 | GE21846 |
| D-serine modulator-1 | 1.524 | 1.817 | GE12850 |
| Dual endothelin 1, angiotensin II receptor | 1.627 | 1.705 | GE17632 |
| dual specificity phosphatase 1 | 2.348 | 1.611 | GE1248349 |
| dynamin 3 | 1.788 | 1.472 | GE1174180 |
| Early growth response 3 | 1.634 | 1.495 | GE1265585 |
| EST350850 Rat gene index, normalized rat, norvegicus, Bento Soares Rattus norvegicus cDNA clone RGIFY73 5' end, mRNA sequence. | 0.597 | 0.606 | GE1262370 |
| EST531153 Rat gene index, normalized rat, norvegicus Rattus norvegicus cDNA clone RGIAA90 5' end similar to mitochondrial D-loop nascent strand-like region, mRNA sequence. | 0.594 | 0.655 | GE1170372 |
| Far upstream element (FUSE) binding protein 1 | 1.587 | 1.435 | GE17389 |
| FBJ murine osteosarcoma viral oncogene homolog | 2.651 | 1.534 | GE18981 |
| FBJ murine osteosarcoma viral oncogene homolog | 2.535 | 1.501 | GE16552 |
| F-box protein 46 | 0.362 | 0.474 | GE1288733 |
| Fibroblast growth factor receptor substrate 3 | 0.69 | 0.713 | GE1292727 |
| Fibronectin type III and SPRY domain containing 2 (predicted) | 0.698 | 0.683 | GE1133626 |
| glutamate cysteine ligase, modifier subunit | 1.592 | 1.43 | GE20488 |
| glutamate receptor, ionotropic, N-methyl D-aspartate 2B | 2.481 | 1.854 | GE1205376 |
| glycoprotein 2 (zymogen granule membrane) | 0.634 | 0.703 | GE1137686 |
| heat shock 70kDa protein 5 binding protein 1 | 1.586 | 1.405 | GE1154612 |
| Hermansky-Pudlak syndrome 1 homolog (human) | 0.691 | 0.684 | GE1158633 |
| high mobility group AT-hook 1 | 1.566 | 1.471 | GE1132911 |
| Hypothetical LOC292874 (predicted) | 2.985 | 3.01 | GE12769 |
| kalirin, RhoGEF kinase | 0.655 | 0.644 | GE13496 |
| kidney specific organic anion transporter | 1.757 | 1.421 | GE19518 |
| Leucine rich repeat neuronal 1 | 0.557 | 0.664 | GE1262406 |
| microtubule-associated protein 1 light chain 3 alpha | 1.533 | 1.467 | GE1175605 |
| Mitochondrial protein, 18 kDa | 1.445 | 1.418 | GE12796 |
| Mitogen activated protein kinase kinase kinase kinase 2 (predicted) | 0.53 | 0.655 | GE1170412 |
| Mitogen-activated protein kinase kinase kinase 14 (predicted) | 0.594 | 0.644 | GE1101647 |
| Muscle and microspikes RAS | 1.665 | 1.421 | GE15048 |
| muscle, skeletal, receptor tyrosine kinase | 0.65 | 0.617 | GE20914 |
| Myomesin 1 (skelemin) 185kDa | 0.65 | 0.657 | GE1270223 |
| NADH dehydrogenase (ubiquinone) 1 beta subcomplex 3 (predicted) | 1.433 | 1.427 | GE1186325 |
| NADH dehydrogenase (ubiquinone) 1 beta subcomplex, 2 (predicted) | 1.833 | 1.851 | GE15461 |
| NADH dehydrogenase (ubiquinone) Fe-S protein 3 (predicted) | 1.428 | 1.428 | GE1159927 |
| Nuclear receptor coactivator 3 | 0.664 | 0.662 | GE1247284 |
| Odd Oz/ten-m homolog 2 (Drosophila) | 0.603 | 0.679 | GE1217075 |
| P55 protein | 1.578 | 1.516 | GE14687 |
| Phosphoinositide-3-kinase, regulatory subunit 4, p150 (predicted) | 0.703 | 0.7 | GE1247893 |
| potassium inwardly-rectifying channel, subfamily J, member 6 | 0.617 | 0.694 | GE20238 |
| Potassium voltage-gated channel, shaker-related subfamily, beta member 1 | 0.549 | 0.683 | GE1188737 |
| POU domain, class 3, transcription factor 3 | 0.612 | 0.702 | GE1269907 |
| Procollagen, type IV, alpha 3 (Goodpasture antigen) binding protein (predicted) | 1.706 | 1.668 | GE1205418 |
| Procollagen, type VI, alpha 1 (predicted) | 1.506 | 1.598 | GE15511 |
| Progesterone receptor | 1.551 | 1.629 | GE1110148 |
| Protein disulfide isomerase associated 4 | 0.664 | 0.705 | GE1172620 |
| protein phosphatase 1, regulatory (inhibitor) subunit 12A | 1.601 | 1.59 | GE21994 |
| Protein phosphatase 2 (formerly 2A), regulatory subunit B (PR 52), beta isoform | 1.669 | 1.566 | GE1144404 |
| Protocadherin gamma subfamily C, 3 | 0.603 | 0.698 | GE1237378 |
| Pseudouridylate synthase 7 homolog (S. cerevisiae) (predicted) | 2.337 | 2.441 | GE1172168 |
| putative pheromone receptor (Go-VN5) | 0.615 | 0.663 | GE13181 |
| queuine tRNA-ribosyltransferase 1 | 1.457 | 1.453 | GE1187376 |
| Ras homolog gene family, member C (predicted) | 1.619 | 1.627 | GE12629 |
| Ras homolog gene family, member G | 1.574 | 1.413 | GE17940 |
| Rho-associated coiled-coil forming kinase 2 | 1.773 | 1.449 | GE20122 |
| Ribosomal protein L27a (predicted) | 1.455 | 1.529 | GE1203933 |
| Ring finger protein 1 | 1.972 | 1.588 | GE1305362 |
| RT1 class II, locus Da | 0.643 | 0.594 | GE21186 |
| Ser/Thr-like protein kinase lyk4 | 1.704 | 1.496 | GE1157247 |
| SERTA domain containing 1 | 1.583 | 1.456 | GE12856 |
| SH3-domain GRB2-like 1 | 1.511 | 1.596 | GE1170336 |
| Signal-induced proliferation-associated 1 like 1 | 1.471 | 1.416 | GE1284364 |
| Signal-induced proliferation-associated 1 like 1 | 0.547 | 0.645 | GE1169078 |
| Similar to 1700123O20Rik protein (predicted) | 0.6 | 0.71 | GE1277747 |
| Similar to cDNA sequence BC004044 | 1.59 | 1.544 | GE14545 |
| Similar to dehydrogenase/reductase (SDR family) member 10 | 0.559 | 0.541 | GE1143489 |
| Similar to Delta-interacting protein A (Hepatitis delta antigen interacting protein A) (predicted) | 1.408 | 1.483 | GE1206638 |
| Similar to DNA segment, Chr 4, ERATO Doi 22, expressed (predicted) | 1.964 | 1.786 | GE1177005 |
| Similar to DnaJ (Hsp40) homolog, subfamily B, member 12 | 1.539 | 1.445 | GE12398 |
| Similar to E430002G05Rik protein (predicted) | 0.634 | 0.696 | GE1210573 |
| Similar to Endoplasmic reticulum mannosyl-oligosaccharide 1,2-alpha-mannosidase (ER alpha-1,2-mannosidase) (predicted) | 1.885 | 1.571 | GE17424 |
| Similar to expressed sequence AI317237 (predicted) | 1.541 | 1.516 | GE1199326 |
| Similar to HCV NS3-transactivated protein 1 (predicted) | 1.508 | 1.408 | GE1239667 |
| Similar to hypothetical protein | 1.69 | 1.586 | GE19118 |
| Similar to hypothetical protein FLJ11526 (predicted) | 1.634 | 1.52 | GE1118411 |
| Similar to hypothetical protein MGC33486 (predicted) | 1.667 | 1.513 | GE15640 |
| Similar to lipoma HMGIC fusion partner-like 3 (predicted) | 0.703 | 0.697 | GE1177763 |
| Similar to myocyte enhancer factor 2C | 2.112 | 1.873 | GE14761 |
| Similar to myosin heavy chain Myr 8 | 2.032 | 1.768 | GE1108210 |
| Similar to Phosphatidylserine decarboxylase proenzyme | 0.567 | 0.649 | GE1180632 |
| Similar to RAD54B homolog isoform 1; RAD54, S. cerevisiae, homolog of, B (predicted) | 1.822 | 1.884 | GE18389 |
| Similar to RIKEN cDNA 1700012G19 gene (predicted) | 1.465 | 1.518 | GE19075 |
| Similar to RIKEN cDNA 5830433M19 | 1.63 | 1.529 | GE1110831 |
| Similar to SLIT-ROBO Rho GTPase-activating protein 1 (predicted) | 0.667 | 0.667 | GE1272962 |
| Similar to sperm antigen HCMOGT-1 | 1.515 | 1.583 | GE1229804 |
| SNAP25-interacting protein | 1.445 | 1.588 | GE1206582 |
| Solute carrier family 14 (urea transporter), member 2 | 0.574 | 0.634 | GE13494 |
| solute carrier family 35, member E4 | 1.491 | 1.503 | GE13436 |
| solute carrier family 4, member 3 | 1.451 | 1.458 | GE20303 |
| Son cell proliferation protein | 1.486 | 1.472 | GE15970 |
| Sortilin-related receptor, L(DLR class) A repeats-containing (predicted) | 1.567 | 1.549 | GE1135618 |
| Sortilin-related VPS10 domain containing receptor 2 (predicted) | 0.458 | 0.441 | GE1156497 |
| Sp1 transcription factor | 1.518 | 1.706 | GE1223041 |
| spectrin beta 3 | 1.455 | 1.494 | GE20563 |
| Tax1 (human T-cell leukemia virus type I) binding protein 3 | 1.561 | 1.43 | GE15966 |
| TBC1 domain family, member 1 (predicted) | 1.768 | 1.606 | GE1292184 |
| TBC1 domain family, member 20 | 1.493 | 1.418 | GE1152795 |
| Thioredoxin 2 | 0.655 | 0.669 | GE16942 |
| Thyroid hormone receptor associated protein 5 (predicted) | 1.709 | 1.511 | GE1186469 |
| Transcribed locus | 2.051 | 1.953 | GE1219601 |
| Transcribed locus | 1.979 | 1.951 | GE1158189 |
| Transcribed locus | 1.892 | 1.864 | GE14651 |
| Transcribed locus | 1.82 | 1.683 | GE1167981 |
| Transcribed locus | 1.747 | 1.616 | GE17969 |
| Transcribed locus | 1.741 | 1.601 | GE13932 |
| Transcribed locus | 1.688 | 1.577 | GE16696 |
| Transcribed locus | 1.683 | 1.566 | GE14229 |
| Transcribed locus | 1.628 | 1.565 | GE12740 |
| Transcribed locus | 1.619 | 1.481 | GE13847 |
| Transcribed locus | 1.59 | 1.472 | GE1206645 |
| Transcribed locus | 1.553 | 1.472 | GE19157 |
| Transcribed locus | 1.539 | 1.462 | GE1233738 |
| Transcribed locus | 1.504 | 1.434 | GE18744 |
| Transcribed locus | 1.503 | 1.423 | GE1295381 |
| Transcribed locus | 1.482 | 1.41 | GE1233953 |
| Transcribed locus | 1.457 | 1.407 | GE1257582 |
| Transcribed locus | 1.409 | 1.403 | GE1225943 |
| Transcribed locus | 1.401 | 1.401 | GE1108245 |
| Transcribed locus | 0.696 | 0.706 | GE1138867 |
| Transcribed locus | 0.695 | 0.704 | GE12945 |
| Transcribed locus | 0.679 | 0.697 | GE14688 |
| Transcribed locus | 0.666 | 0.689 | GE1235811 |
| Transcribed locus | 0.625 | 0.677 | GE1242132 |
| Transcribed locus | 0.587 | 0.664 | GE1222096 |
| Transcribed locus | 0.549 | 0.659 | GE1252475 |
| Transcribed locus | 0.499 | 0.514 | GE1164752 |
| Transcribed locus, moderately similar to XP_546425.2 PREDICTED: similar to roundabout, axon guidance receptor, homolog 3 [Canis familiaris] | 1.998 | 1.906 | GE1287744 |
| Transcribed locus, strongly similar to NP_003890.1 Rho guanine nucleotide exchange factor 7 isoform a [Homo sapiens] | 1.433 | 1.408 | GE17327 |
| Transcribed locus, strongly similar to NP_071949.1 ribosomal protein L36 [Rattus norvegicus] | 0.598 | 0.552 | GE1108316 |
| Transcribed locus, strongly similar to XP_226471.2 PREDICTED: similar to Mtr3 (mRNA transport regulator 3)-homolog [Rattus norvegicus] | 0.616 | 0.568 | GE1227353 |
| Transcribed locus, strongly similar to XP_343328.1 PREDICTED: desert hedgehog homolog [Rattus norvegicus] | 0.704 | 0.704 | GE1179703 |
| Transcribed locus, strongly similar to XP_580013.1 PREDICTED: hypothetical protein XP_580013 [Rattus norvegicus] | 0.594 | 0.685 | GE1136098 |
| Transcribed locus, weakly similar to NP_001003141.1 protein tyrosine kinase fer [Canis familiaris] | 0.657 | 0.666 | GE16643 |
| Transcribed locus, weakly similar to XP_529735.1 PREDICTED: hypothetical protein XP_529735 [Pan troglodytes] | 0.647 | 0.696 | GE1244031 |
| Transcribed locus, weakly similar to XP_576810.1 PREDICTED: similar to RIKEN cDNA 4930555G01 [Rattus norvegicus] | 1.638 | 1.496 | GE1219990 |
| Transcribed locus, weakly similar to XP_580018.1 PREDICTED: hypothetical protein XP_580018 [Rattus norvegicus] | 1.445 | 1.979 | GE1248493 |
| transcription factor 4 | 1.589 | 1.551 | GE20851 |
| Transient receptor potential cation channel, subfamily C, member 4 associated protein | 1.565 | 1.458 | GE1181121 |
| Treacher Collins Franceschetti syndrome 1, homolog (predicted) | 0.684 | 0.601 | GE12972 |
| Tumor protein p53 inducible protein 13 (predicted) | 1.693 | 1.634 | GE14715 |
| Tweety homolog 3 (Drosophila) (predicted) | 0.549 | 0.616 | GE1213706 |
| Type I keratin KA15 | 0.471 | 0.656 | GE16317 |
| tyrosine 3-monooxygenase/tryptophan 5-monooxygenase activation protein, eta polypeptide | 1.575 | 1.448 | GE20141 |
| UI-R-C2p-qz-f-05-0-UI.r1 UI-R-C2p Rattus norvegicus cDNA clone UI-R-C2p-qz-f-05-0-UI 5', mRNA sequence. | 1.59 | 1.553 | GE1280874 |
| UI-R-E1-fe-b-07-0-UI.s1 UI-R-E1 Rattus norvegicus cDNA clone UI-R-E1-fe-b-07-0-UI 3', mRNA sequence. | 0.572 | 0.544 | GE1116227 |
| Vacuolar protein sorting 4a (yeast) | 1.74 | 1.419 | GE12718 |
| Vesicle transport through interaction with t-SNAREs homolog 1A (yeast) | 1.513 | 1.537 | GE1224645 |
| WW domain containing E3 ubiquitin protein ligase 2 (predicted) | 0.639 | 0.681 | GE1150970 |
| Zinc finger homeobox 2 | 1.766 | 1.436 | GE15661 |

**NAc**

Naïve vs. 1-day

| **Gene** | **Alias** | **Accession #** | **Fold Change** | **Probe ID** |
| --- | --- | --- | --- | --- |
| Actinin, alpha 1 | Actn1 | BE119541 | 0.714 | GE1122643 |
| AMGNNUC:NRHY1-00109-B12-A W Rat hypothalamus (10480) Rattus norvegicus cDNA clone nrhy1-00109-b12 5' |  | CB796752 | 0.675 | GE1177181 |
| B-cell translocation gene 2, anti-proliferative | An; Agl; Pc3; an-1; Tis21 | NM_017259 | 1.75 | GE20453 |
| BM259 protein | Bm259 | BE119385 | 0.587 | GE1163730 |
| CDKN1A interacting zinc finger protein 1 (predicted) | Ciz1_predicted | BF389892 | 1.432 | GE1221544 |
| Complement factor D (adipsin) | Cfd | BM391572 | 1.617 | GE1112269 |
| Cyclin-dependent kinase 5, regulatory subunit 1 (p35) | Cdk5r1 | CB746161 | 0.612 | GE1201166 |
| Cytochrome b-561 domain containing 2 | Cyb561d2 | BG377940 | 1.4 | GE1262585 |
| discs, large (Drosophila) homolog-associated protein 3 | Dap3; DAP-3; SAPAP3 | NM_173138 | 0.698 | GE21005 |
| dual specificity phosphatase 1 | Mkp1; CL100; MKP-1; 3CH134; Ptpn16 | NM_053769 | 1.82 | GE1248349 |
| Dystrobrevin, beta | Dtnb | BE112436 | 1.419 | GE1193731 |
| EST455273 Rat Gene Index, normalized rat, Rattus norvegicus cDNA Rattus norvegicus cDNA clone RGIHX85 3' sequence |  | BF290682 | 1.402 | GE1194682 |
| Exosome component 4 (predicted) | Exosc4_predicted | BG379644 | 1.421 | GE18789 |
| FBJ murine osteosarcoma viral oncogene homolog | Fos | BF415939 | 1.888 | GE18981 |
| Fibronectin type III domain containing 1 | Fndc1 | BM391732 | 1.637 | GE1273897 |
| Glutamate-ammonia ligase (glutamine synthase) | Glul | BF563467 | 1.402 | GE19389 |
| guanylate cyclase 1, soluble, alpha 3 | SGC; Gucy1a1 | NM_017090 | 1.407 | GE20333 |
| Gup1, glycerol uptake/transporter homolog (yeast) (predicted) | Gup1_predicted | AI172352 | 1.627 | GE14360 |
| HnRNP-associated with lethal yellow | Raly | BF417338 | 1.442 | GE13844 |
| homeo box, msh-like 1 | Msx1 | NM_031059 | 0.697 | GE21611 |
| Hypothetical LOC300663 | RGD1305351 | AA924450 | 0.565 | GE19281 |
| Interferon regulatory factor 7 | Irf7 | BF284803 | 1.699 | GE18258 |
| Intersex-like (Drosophila) (predicted) | Ixl_predicted | AW524989 | 1.411 | GE1235224 |
| Kruppel-like factor 4 (gut) | GKLF; MGC93286 | NM_053713 | 1.488 | GE14597 |
| LOC361774 (predicted) | RGD1306116_predicted | BF549121 | 1.406 | GE19160 |
| Lysyl oxidase-like 1 | Loxl1 | AI599031 | 1.536 | GE15532 |
| melanocortin 4 receptor | Mc4r | NM_013099 | 1.527 | GE22026 |
| melanoma inhibitory activity 1 | Mia; Cdrap | NM_030852 | 1.451 | GE21007 |
| Monoamine oxidase A | Maoa | CA507269 | 0.709 | GE1103943 |
| RAB22A, member RAS oncogene family (predicted) | Rab22a_predicted | AI235347 | 1.429 | GE14891 |
| RAS guanyl releasing protein 2 (calcium and DAG-regulated) (predicted) | Rasgrp2_predicted | BF524415 | 1.566 | GE19107 |
| Receptor transporter protein 4 (predicted) | Rtp4_predicted | BI299688 | 1.533 | GE1244655 |
| ribosomal protein L18 | Rpl18 | NM_031102 | 1.405 | GE1124008 |
| RT1 class Ib, locus Aw2 | RT1-Aw2 | BI275216 | 1.683 | GE1280096 |
| S100 calcium binding protein A16 (predicted) | S100a16_predicted | AI406499 | 1.427 | GE15006 |
| Sec11-like 1 (S. cerevisiae) | Spc18 | NM_031723 | 1.49 | GE21683 |
| serine/threonine kinase 16 | F52; PKL12 | NM_173142 | 1.411 | GE1273797 |
| Similar to Agrin (predicted) | RGD1306592_predicted | AW434898 | 1.419 | GE1202035 |
| Similar to Cartilage-associated protein precursor (predicted) | RGD1565180_predicted | BM385740 | 1.405 | GE17261 |
| Similar to cDNA sequence BC025641 | RGD1306730 | CF111601 | 1.427 | GE1217839 |
| Similar to hypothetical protein | MGC94915 | BE107768 | 0.633 | GE1269993 |
| Similar to hypothetical protein FLJ30656 (predicted) | RGD1309441_predicted | BE113248 | 1.432 | GE17790 |
| Similar to novel protein (predicted) | RGD1560608_predicted | BF410717 | 0.606 | GE1286816 |
| Similar to potassium voltage-gated channel, Isk-related family, member 1-like | LOC681190 | AI710064 | 1.526 | GE18048 |
| Similar to Prefoldin subunit 2 | LOC685607 | CB607836 | 1.471 | GE1188535 |
| Similar to Probable serine/threonine-protein kinase KIAA1811 (predicted) | RGD1563268_predicted | CB613769 | 0.67 | GE1166867 |
| Similar to RIKEN cDNA 9430031J16 (predicted) | RGD1310964_predicted | CB718281 | 0.516 | GE1161553 |
| Similar to RIKEN cDNA A430005L14 | RGD1304567 | BF414161 | 1.414 | GE18766 |
| Similar to RIKEN cDNA B230118H07 (predicted) | RGD1309730_predicted | BF418835 | 1.59 | GE19341 |
| Tenascin C | Tnc | AA892824 | 1.469 | GE12674 |
| Tetraspanin 5 | Tspan5 | AW528482 | 1.503 | GE1191963 |
| Thioredoxin domain containing 1 | Txndc1 | AI236083 | 1.535 | GE14915 |
| Transcribed locus | Gpd1 | BM390544 | 2.38 | GE1206187 |
| Transcribed locus |  | BQ209985 | 1.568 | GE1290535 |
| Transcribed locus |  | BF386024 | 1.445 | GE1299295 |
| Transcribed locus |  | BQ195587 | 1.44 | GE18787 |
| Transcribed locus |  | BI273760 | 1.439 | GE18797 |
| Transcribed locus |  | BF396948 | 1.425 | GE18553 |
| Transcribed locus |  | AW917673 | 0.706 | GE16819 |
| Transcribed locus |  | BF285935 | 0.691 | GE1250104 |
| Transcribed locus |  | BF405604 | 0.664 | GE1199090 |
| Transcribed locus |  | CB579259 | 0.586 | GE1166857 |
| Transcribed locus |  | BU671238 | 0.559 | GE1151491 |
| Transcribed locus, strongly similar to XP_238535.3 PREDICTED: similar to ankyrin repeat domain 25 [Rattus norvegicus] |  | AW920557 | 1.422 | GE17050 |
| Transducin (beta)-like 1 X-linked (predicted) | Tbl1x_predicted | BF388744 | 0.695 | GE1260210 |
| UI-R-C4-alb-e-04-0-UI.r1 UI-R-C4 Rattus norvegicus cDNA clone UI-R-C4-alb-e-04-0-UI 5', mRNA sequence. |  | BF565848 | 17.54 | GE1304612 |
| UI-R-E1-fg-c-12-0-UI.s1 UI-R-E1 Rattus norvegicus cDNA clone UI-R-E1-fg-c-12-0-UI 3', mRNA sequence. |  | AA955744 | 0.684 | GE1141489 |
| Vascular early response gene protein | Verge | AW523915 | 1.806 | GE1279845 |

**NAc**

**Naïve vs. 10-day**

| **Gene** | **Alias** | **Accession #** | **Fold Change** | **Probe ID** |
| --- | --- | --- | --- | --- |
| 7-dehydrocholesterol reductase | MGC93039 | NM_022389 | 1.462 | GE1237543 |
| Abhydrolase domain containing 7 (predicted) | Abhd7_predicted | BF563879 | 1.424 | GE1115306 |
| Achalasia, adrenocortical insufficiency, alacrimia (Allgrove, triple-A) (predicted) | Aaas_predicted | BF283385 | 1.423 | GE18175 |
| acidic ribosomal phosphoprotein P0 | Arbp | NM_022402 | 0.631 | GE1282452 |
| Actinin alpha 2 (predicted) | Actn2_predicted | BF284889 | 1.61 | GE18272 |
| Acyl-Coenzyme A oxidase 3, pristanoyl | Acox3 | AW916127 | 1.651 | GE16683 |
| Adducin 1 (alpha) | Add1 | BF562284 | 1.468 | GE1301349 |
| AGENCOURT_17635429 NIH_MGC_235 Rattus norvegicus cDNA clone IMAGE:7109457 5', mRNA sequence. | Hadha | CK483660 | 0.641 | GE20527 |
| aldehyde dehydrogenase 2 | Aldh2 | NM_032416 | 1.67 | GE1233937 |
| aldolase A | Aldo1; RNALDOG5 | NM_012495 | 0.631 | GE1233936 |
| AMGNNUC:MRMN3-00020-G5-A mrmn3 (10218) Rattus norvegicus cDNA clone mrmn3-00020-g5 5', mRNA sequence. | Fgf12 | CB697972 | 0.664 | GE1113024 |
| Arylacetamide deacetylase-like 1 (predicted) | Aadacl1_predicted | CB747046 | 0.702 | GE1269328 |
| ATG16 autophagy related 16-like 1 (S. cerevisiae) (predicted) | Atg16l1_predicted | BU759880 | 1.409 | GE19325 |
| ATPase family, AAA domain containing 1 | Atad1 | BF289272 | 0.691 | GE18383 |
| ATPase type 13A1 (predicted) | Atp13a1_predicted | AA893621 | 1.477 | GE12710 |
| ATPase, Ca++ transporting, plasma membrane 1 | Atp2b1 | AI136859 | 1.491 | GE1299332 |
| ATPase, H+ transporting, lysosomal V0 subunit A1 | Atp6n1; Atp6n1a | NM_031604 | 1.458 | GE19712 |
| ATPase, Na+/K+ transporting, alpha 2 polypeptide | RATATPA2 | NM_012505 | 0.68 | GE19791 |
| ATP-binding cassette, sub-family B (MDR/TAP), member 9 | Tapl | NM_022238 | 1.854 | GE1140540 |
| baculoviral IAP repeat-containing 5 | AP14 | NM_022274 | 1.401 | GE1300831 |
| B-cell receptor-associated protein 31 | Bcap31 | H35185 | 0.702 | GE15185 |
| benzodiazepine receptor, peripheral | MBR; Ptbzr; PTBZR02; RATPTBZR02 | NM_012515 | 0.682 | GE19794 |
| brain-specific angiogenesis inhibitor 1-associated protein 2 | Irsp53 | NM_057196 | 1.625 | GE15873 |
| Bromodomain containing 8 | Brd8 | D86668 | 1.412 | GE1227012 |
| BTB (POZ) domain containing 3 (predicted) | Btbd3_predicted | AW915047 | 0.467 | GE16517 |
| cadherin 23 (otocadherin) | W | NM_053644 | 0.686 | GE1168339 |
| Calcium/calmodulin-dependent protein kinase IV | Camk4 | BF409573 | 1.406 | GE1126485 |
| calcium/calmodulin-dependent protein kinase kinase 2, beta | Camkk2 | NM_031338 | 1.579 | GE1150517 |
| Calcyclin binding protein | Cacybp | BG668238 | 0.629 | GE1182366 |
| Calcyclin binding protein | Cacybp | AW918443 | 0.459 | GE16911 |
| Carnitine acetyltransferase | Crat | BG374943 | 1.444 | GE15303 |
| Cell division cycle 2-like 6 (CDK8-like) (predicted) | Cdc2l6_predicted | BE117167 | 1.421 | GE12731 |
| Cell division cycle 6 homolog (S. cerevisiae) (predicted) | Cdc6_predicted | BE117020 | 0.657 | GE1175040 |
| cell growth regulator with EF hand domain 1 | Cgr11 | NM_139087 | 0.646 | GE20997 |
| Centaurin, beta 5 (predicted) | Centb5_predicted | BQ211239 | 1.407 | GE1278000 |
| Chromobox homolog 6 | Cbx6 | BF283454 | 1.49 | GE18184 |
| Cleavage and polyadenylation specific factor 2 (predicted) | Cpsf2_predicted | BU759468 | 1.474 | GE1141348 |
| cocaine and amphetamine regulated transcript | Cart | NM_017110 | 1.66 | GE20347 |
| Colony stimulating factor 1 (macrophage) | Csf1 | BQ207341 | 0.689 | GE1200712 |
| Cyclin M3 (predicted) | Cnnm3_predicted | BE112177 | 1.422 | GE1186459 |
| cytochrome c oxidase subunit VIIb | Cox7b | NM_182819 | 0.671 | GE12579 |
| Cytomatrix protein p110 | Cmbp | AW527656 | 1.411 | GE1179302 |
| DAZ interacting protein 1 | Dzip1 | BE109120 | 1.552 | GE17524 |
| DEAH (Asp-Glu-Ala-His) box polypeptide 35 (predicted) | Dhx35_predicted | AW525179 | 1.444 | GE1120398 |
| Death associated transcription factor 1 (predicted) | Datf1_predicted | CB741625 | 1.509 | GE1289366 |
| dickkopf homolog 3 (Xenopus laevis) | p29; Reic | NM_138519 | 1.505 | GE13508 |
| dihydropyrimidinase-like 5 | Crmp5; Ulip6 | NM_023023 | 1.463 | GE1257977 |
| distal-less homeobox 5 | RDLX | NM_012943 | 1.495 | GE1208545 |
| dopamine receptor D1A | D1a; Drd1; Drd-1 | NM_012546 | 1.596 | GE1273034 |
| Doublecortin and CaM kinase-like 3 (predicted) | Dcamkl3_predicted | BF566030 | 1.471 | GE1304375 |
| Down syndrome critical region homolog 6 (human) (predicted) | Dscr6_predicted | BE111863 | 0.701 | GE12835 |
| Ectodermal-neural cortex 1 | Enc1 | BF408402 | 1.431 | GE14609 |
| ectonucleotide pyrophosphatase/phosphodiesterase 2 | MGC93258 | NM_057104 | 0.659 | GE19483 |
| EGF-like module containing, mucin-like, hormone receptor-like sequence 1 | Emr1 | BE100625 | 0.682 | GE1292466 |
| Eph receptor A7 | Epha7 | NM_134331 | 1.414 | GE1295941 |
| ERBB receptor feedback inhibitor 1 | Errfi1 | CA508583 | 1.403 | GE1257314 |
| EST291160 Normalized rat brain, Bento Soares Rattus sp. cDNA clone RGIBD01 5' end similar to H.sapiens hypothetical protein KIAA0383 |  | AW141128 | 0.662 | GE15757 |
| eukaryotic translation elongation factor 1 alpha 2 | Ps10; Stnl; RATPS10 | NM_012660 | 0.69 | GE1220113 |
| eukaryotic translation initiation factor 2 alpha kinase 1 | Hri | NM_013223 | 1.427 | GE1279626 |
| Eyes absent 2 homolog (Drosophila) | Eya2 | BF386078 | 1.401 | GE1110950 |
| F-box only protein 22 | Fbxo22 | BQ195344 | 1.467 | GE1234917 |
| F-box protein 46 | Fbxo46 | BF564168 | 0.619 | GE1288733 |
| FK506 binding protein 5 | Fkbp5 | BF551250 | 1.433 | GE19218 |
| flavin containing monooxygenase 1 | RFMO1A | NM_012792 | 1.551 | GE21850 |
| Follistatin-like 1 | Fstl1 | BQ207459 | 1.547 | GE1118048 |
| Follistatin-like 4 (predicted) | Fstl4_predicted | BF411514 | 1.512 | GE1175210 |
| Friend leukemia integration 1 | Fli1 | AA818789 | 1.466 | GE1199176 |
| Ftsj homolog 1 (E. coli) (predicted) | Ftsj1_predicted | BU759770 | 0.645 | GE1257319 |
| G protein-coupled receptor 56 | Gpr56 | NM_152242 | 1.524 | GE1141408 |
| glucosamine | Uae1 | NM_053765 | 1.416 | GE21189 |
| glutaminase 2 (liver, mitochondrial) | Ga | NM_138904 | 0.651 | GE19583 |
| Golgi SNAP receptor complex member 1 | Gosr1 | AI169291 | 0.672 | GE14176 |
| G-protein coupled receptor 88 | Gpr88 | NM_031696 | 1.604 | GE13127 |
| Guanine nucleotide binding protein, beta 1 | Gnb1 | CA508122 | 1.456 | GE1125839 |
| H2A histone family, member Z | MGC72814; MGC105426 | NM_022674 | 0.645 | GE1221561 |
| heterogeneous nuclear ribonucleoprotein A1 | Hnrpa1 | NM_017248 | 0.578 | GE20448 |
| Hippocalcin-like 4 | Hpcal4 | AI044838 | 1.404 | GE1127294 |
| hyaluronan and proteoglycan link protein 2 | Bral1 | AB049056 | 0.375 | GE13140 |
| Hypothetical LOC305452 (predicted) | RGD1309634_predicted | AW523899 | 1.408 | GE16203 |
| Hypothetical protein LOC307347 | LOC307347 | CA509552 | 1.418 | GE1301990 |
| Hypothetical protein LOC679525 | LOC679525 | BF400028 | 1.414 | GE1221933 |
| Inhibitor of growth family, member 3 | Ing3 | BI296426 | 1.518 | GE1258826 |
| Inosine triphosphatase (nucleoside triphosphate pyrophosphatase) (mapped) | Itpa_mapped | AA900405 | 1.5 | GE1125586 |
| inositol 1,4,5-trisphosphate 3-kinase A | Itpka | NM_031045 | 1.448 | GE19685 |
| Inositol polyphosphate-4-phosphatase, type II | Inpp4b | AI716504 | 1.406 | GE1229070 |
| Karyopherin (importin) alpha 4 | Kpna4 | AA851290 | 1.417 | GE1235898 |
| Kelch-like 22 (Drosophila) (predicted) | Klhl22_predicted | CB548033 | 0.67 | GE1267932 |
| Kinesin family member 23 (predicted) | Kif23_predicted | BE113443 | 1.502 | GE1196702 |
| Kinesin heavy chain family, member 2 | Kif2 | BF544320 | 0.559 | GE19125 |
| Leucine rich repeat containing 8 family, member D | Lrrc8d | CA508383 | 1.402 | GE1180450 |
| Like-glycosyltransferase (predicted) | Large_predicted | BF398777 | 1.439 | GE14673 |
| LSM4 homolog, U6 small nuclear RNA associated (S. cerevisiae) (predicted) | Lsm4_predicted | BG373486 | 1.445 | GE1195574 |
| MAF1 homolog (S. cerevisiae) | Maf1 | AI172177 | 1.556 | GE14333 |
| megakaryocyte-associated tyrosine kinase | Batk; MGC105436 | NM_021859 | 1.681 | GE20824 |
| megakaryocyte-associated tyrosine kinase | Batk; MGC105436 | NM_021859 | 1.677 | GE1230939 |
| Methyltransferase like 6 | Mettl6 | AI177369 | 1.433 | GE14511 |
| Methyltransferase-like 3 | Mettl3 | BG381760 | 1.422 | GE1130222 |
| Microspherule protein 1 | Mcrs1 | BQ200835 | 1.488 | GE1129163 |
| Microtubule-associated protein tau | Mapt | AW521314 | 0.665 | GE1194698 |
| Minichromosome maintenance deficient 6 (MIS5 homolog, S. pombe) (S. cerevisiae) | Mcm6 | CA505103 | 1.41 | GE1290882 |
| Mitochondrial ribosomal protein L36 (predicted) | Mrpl36_predicted | BI282120 | 0.663 | GE1297063 |
| Mitochondrial tumor suppressor 1 | Mtus1 | CB577480 | 1.533 | GE1298648 |
| muskelin 1, intracellular mediator containing kelch motifs | Mkln1 | NM_031359 | 0.705 | GE13133 |
| myosin, heavy polypeptide 2, skeletal muscle, adult | LOC691644 | L13606 | 1.431 | GE1262519 |
| Nedd4 family interacting protein 1 | Ndfip1 | AI104295 | 0.661 | GE14001 |
| neurexophilin 4 | Nph4 | NM_021680 | 0.706 | GE20769 |
| Nibrin | Nbn | AW530937 | 1.406 | GE1117339 |
| Nischarin | Nisch | AI237243 | 1.605 | GE14956 |
| Nitric oxide synthase trafficker | Nostrin | BQ209652 | 1.476 | GE1109064 |
| nuclear receptor subfamily 4, group A, member 1 | HMR; Nur77; Ngfi-b | NM_024388 | 2.559 | GE1248064 |
| nuclear receptor subfamily 4, group A, member 3 | NOR-2 | NM_017352 | 1.942 | GE1302764 |
| Nucleoporin 93 | Nup93 | H34909 | 1.428 | GE1177266 |
| Nucleoporin 98 | Nup98 | BE119066 | 1.463 | GE1274925 |
| Paf1, RNA polymerase II associated factor, homolog (S. cerevisiae) | Paf1 | CK480766 | 0.698 | GE1167671 |
| palmitoyl-protein thioesterase 2 | PPT-2 | NM_019367 | 1.668 | GE20687 |
| Pannexin 2 | Panx2 | BQ204587 | 1.445 | GE1156037 |
| Pantothenate kinase 2 (Hallervorden-Spatz syndrome) (predicted) | Pank2_predicted | BG379810 | 1.576 | GE1173101 |
| paralemmin | Palm | NM_130829 | 1.616 | GE16438 |
| Peroxisomal delta3, delta2-enoyl-Coenzyme A isomerase | Peci | AW919017 | 0.703 | GE16975 |
| Peter pan homolog (Drosophila) | Ppan | AI178019 | 1.495 | GE14532 |
| Phosphatase and actin regulator 1 | Phactr1 | BF409384 | 1.559 | GE1177750 |
| phosphodiesterase 10A | Pde10a3 | NM_022236 | 1.687 | GE13095 |
| phospholipase C, gamma 1 | PPLCA | NM_013187 | 1.491 | GE20234 |
| PiggyBac transposable element derived 5 (predicted) | Pgbd5_predicted | AW523614 | 1.443 | GE16190 |
| platelet derived growth factor receptor, beta polypeptide | Pdgfrb | NM_031525 | 1.419 | GE1300875 |
| Platelet-derived growth factor, C polypeptide | Pdgfc | BE118653 | 1.404 | GE1274568 |
| Poly (A) polymerase beta (testis specific) | Papolb | BF406637 | 1.42 | GE18781 |
| Postmeiotic segregation increased 2 (S. cerevisiae) (predicted) | Pms2_predicted | AW916463 | 1.442 | GE16711 |
| Potassium channel tetramerisation domain containing 3 | Kctd3 | BF407757 | 1.507 | GE18806 |
| potassium intermediate/small conductance calcium-activated channel, subfamily N, member 2 | Kcnn2 | NM_019314 | 0.708 | GE20656 |
| Progestin and adipoQ receptor family member V | Paqr5 | BQ189834 | 1.408 | GE1268911 |
| prolactin induced protein | Pip | NM_022708 | 0.665 | GE1221765 |
| prominin 1 | Prom | NM_021751 | 1.407 | GE1211483 |
| prominin 2 | Trprp; Promrp; Prom-rp | NM_138857 | 1.496 | GE1293040 |
| Proprotein convertase subtilisin/kexin type 2 | Pcsk2 | BF412932 | 1.429 | GE1293991 |
| proprotein convertase subtilisin/kexin type3 | Pace; Furin | NM_019331 | 1.486 | GE20664 |
| Protein kinase C, eta | Prkch | AA799981 | 1.466 | GE12253 |
| protein kinase C, zeta | Pkcz; r14-3-3; 14-3-3-zetaisoform | NM_022507 | 1.417 | GE21950 |
| Protein kinase, AMP-activated, beta 2 non-catalytic subunit | Prkab2 | BM386547 | 1.417 | GE14247 |
| Protein phosphatase 1, regulatory (inhibitor) subunit 1A | Ppp1r1a | AW523755 | 1.412 | GE16198 |
| Protein phosphatase 1, regulatory (inhibitor) subunit 1B | Ppp1r1b | BF550795 | 1.574 | GE19206 |
| proteoglycan 2, bone marrow | Prg2 | NM_031619 | 0.703 | GE21607 |
| Pterin 4 alpha carbinolamine dehydratase/dimerization cofactor of hepatocyte nuclear factor 1 alpha (TCF1) 1 | Pcbd1 | BQ211637 | 0.691 | GE1262470 |
| RAB3A, member RAS oncogene family | Rab3a | BF389910 | 1.421 | GE18442 |
| Ras association (RalGDS/AF-6) domain family 2 | Rassf2 | CA505825 | 0.678 | GE1280990 |
| RASD family, member 2 | Rhes | NM_133568 | 1.46 | GE1151848 |
| Receptor interacting protein kinase 5 | Ripk5 | AI103588 | 1.641 | GE1195267 |
| regulator of G-protein signaling 14 | MGC108631 | NM_053764 | 1.67 | GE21068 |
| regulator of G-protein signaling 2 | Rgs2 | NM_053453 | 1.593 | GE13546 |
| Reticulocalbin 3, EF-hand calcium binding domain (predicted) | Rcn3_predicted | AI008125 | 0.667 | GE13610 |
| Retinoic acid receptor, beta | Rarb | BF410198 | 1.61 | GE19050 |
| RNA binding motif protein 24 (predicted) | Rbm24_predicted | AA899570 | 1.492 | GE1100788 |
| SAP30 binding protein (predicted) | Sap30bp_predicted | BF416989 | 1.423 | GE18996 |
| SCY1-like 2 (S. cerevisiae) (predicted) | Scyl2_predicted | AI556596 | 0.644 | GE1156951 |
| SEC23A (S. cerevisiae) (predicted) | Sec23a_predicted | AA819352 | 0.645 | GE1163313 |
| Serine/threonine kinase 38 | Stk38 | BF283631 | 1.422 | GE18192 |
| SH3-domain GRB2-like B1 (endophilin) | Sh3glb1 | AW918345 | 0.588 | GE16898 |
| Signal recognition particle receptor, B subunit | Srprb | H35457 | 1.404 | GE1204686 |
| Signal sequence receptor, alpha | Ssr1 | BF397998 | 0.707 | GE18578 |
| Similar to 106 kDa O-GlcNAc transferase-interacting protein (predicted) | RGD1307844_predicted | AI175534 | 1.432 | GE14400 |
| Similar to ADP-ribosylation factor related protein 2 | LOC689079 | AI103993 | 1.467 | GE13988 |
| Similar to autism susceptibility candidate 2 (predicted) | RGD1561188_predicted | BF397988 | 1.519 | GE1112307 |
| Similar to Beta-2-syntrophin (59 kDa dystrophin-associated protein A1, basic component 2) (Syntrophin 3) (SNT3) (Syntrophin-like) (SNTL) | LOC689421 | BE119495 | 1.524 | GE1285413 |
| Similar to CG11030-PA (predicted) | RGD1310211_predicted | BQ207546 | 1.659 | GE15429 |
| Similar to CG11206-PA | LOC313672 | BF415778 | 0.699 | GE1169235 |
| Similar to CG3740-PA | LOC690000 | BQ203920 | 1.438 | GE16677 |
| Similar to D3Mm3e (predicted) | RGD1561494_predicted | CB579643 | 0.659 | GE1198317 |
| Similar to expressed sequence AW413431 (predicted) | RGD1559841_predicted | BF398087 | 1.679 | GE1226034 |
| Similar to Fantom protein (predicted) | RGD1311099_predicted | BF394682 | 1.405 | GE1196172 |
| Similar to GTP-binding protein (predicted) | RGD1562380_predicted | BQ201081 | 1.426 | GE1264710 |
| Similar to HN1-like protein | RGD1305117 | CB615228 | 0.691 | GE1171839 |
| Similar to hypothetical protein FLJ10006 | RGD1304762 | BQ209899 | 1.445 | GE1105583 |
| Similar to hypothetical protein FLJ14146 | RGD1310587 | BE097553 | 1.635 | GE17140 |
| Similar to hypothetical protein MGC17299 (predicted) | RGD1311937_predicted | CK480439 | 0.676 | GE1131733 |
| Similar to hypothetical protein MGC34646 (predicted) | RGD1306801_predicted | BU760139 | 1.402 | GE1180773 |
| Similar to IQ motif and Sec7 domain 2 | LOC685244 | BF398881 | 1.537 | GE17052 |
| Similar to KIAA0802 protein (predicted) | RGD1308319_predicted | BF394681 | 1.57 | GE15859 |
| Similar to leucine-rich repeat transmembrane neuronal 1 | LOC679668 | BF286131 | 1.456 | GE18331 |
| Similar to Lmo6 protein | LOC317380 | AI555009 | 1.421 | GE15434 |
| Similar to mKIAA0704 protein (predicted) | RGD1564287_predicted | AI549207 | 1.482 | GE1182990 |
| Similar to mKIAA0738 protein (predicted) | RGD1565474_predicted | AA943126 | 0.673 | GE12787 |
| Similar to open reading frame 5 | RGD1305062 | BQ201056 | 1.415 | GE1193984 |
| Similar to porcupine-D (predicted) | RGD1564947_predicted | BF412142 | 0.597 | GE15400 |
| Similar to protocadherin beta 9 | LOC680047 | BI296743 | 0.452 | GE1165730 |
| Similar to RAS-like, estrogen-regulated, growth-inhibitor (predicted) | RGD1562829_predicted | AW525924 | 1.404 | GE1136879 |
| Similar to RIKEN cDNA 1110055N21 (predicted) | RGD1309370_predicted | BF416880 | 1.4 | GE18994 |
| Similar to RIKEN cDNA 2310005P05 | RGD1307401 | BI275669 | 1.469 | GE1174967 |
| Similar to RIKEN cDNA 5730557B15 (predicted) | RGD1564227_predicted | BF283310 | 1.495 | GE1113008 |
| Similar to RIKEN cDNA 6720467C03 (predicted) | RGD1310681_predicted | AW917256 | 0.671 | GE16772 |
| Similar to RIKEN cDNA B230114P05 (predicted) | RGD1566296_predicted | AW531767 | 1.405 | GE1188602 |
| Similar to Sec1 family domain containing protein 2 (Syntaxin binding protein 1-like 1) (Neuronal Sec1) | LOC498353 | CB569439 | 0.662 | GE1112384 |
| Similar to septin 6 | LOC691335 | AW919881 | 1.674 | GE17025 |
| Similar to TAFA2 protein | LOC680647 | CB607097 | 0.642 | GE1250196 |
| Similar to transmembrane protein 41a | LOC681708 | AI600085 | 1.417 | GE15579 |
| Similar to Tumor protein D53 (mD53) (Tumor protein D52-like 1) | LOC689256 | BF405177 | 1.504 | GE18748 |
| Similar to U2 small nuclear ribonucleoprotein auxiliary factor 35 kDa subunit related-protein 1 (U2(RNU2) small nuclear RNA auxillary factor 1-like 1) (SP2) | LOC498425 | H34285 | 0.56 | GE14773 |
| Similar to UBX domain containing 1 | LOC363332 | H31923 | 1.508 | GE14642 |
| SNF1-like kinase | Sik | NM_021693 | 1.422 | GE20777 |
| sodium channel, voltage-gated, type I, alpha | Scn1a | NM_030875 | 0.697 | GE19675 |
| solute carrier family 25 (mitochondrial carrier, phosphate carrier), member 25 | Mcsc; Pcscl | NM_145677 | 1.602 | GE12341 |
| Splicing factor 3a, subunit 1 (predicted) | Sf3a1_predicted | BE116918 | 1.401 | GE17911 |
| Stanniocalcin 1 | Stc1 | BM386683 | 1.408 | GE1158856 |
| Step II splicing factor SLU7 (S. cerevisiae) | Slu7 | BQ211515 | 1.93 | GE1170125 |
| stress 70 protein chaperone, microsome-associated, 60kD human homolog | Stch | NM_019271 | 0.641 | GE20623 |
| Striatin, calmodulin binding protein 4 (predicted) | Strn4_predicted | CA505964 | 1.527 | GE15467 |
| Stromal interaction molecule 2 (predicted) | Stim2_predicted | AI234719 | 1.429 | GE14875 |
| SWI/SNF related, matrix associated, actin dependent regulator of chromatin, subfamily c, member 1 (predicted) | Smarcc1_predicted | AA965063 | 1.4 | GE12966 |
| syntaxin 1B2 | Stx2; Stx1b | NM_012700 | 1.572 | GE19909 |
| Thioesterase superfamily member 2 (predicted) | Them2_predicted | BG374763 | 0.705 | GE15272 |
| Thymoma viral proto-oncogene 2 | Akt2 | CB808947 | 1.8 | GE12923 |
| tight junction protein 2 | ZO-2; MGC124724 | U75916 | 0.699 | GE1196990 |
| TRAF family member-associated Nf-kappa B activator | Tank | NM_145788 | 0.711 | GE1278921 |
| Transcribed locus |  | BE113211 | 9.113 | GE1284198 |
| Transcribed locus |  | BF416935 | 1.663 | GE18995 |
| Transcribed locus | Mgll | AW919036 | 1.656 | GE16976 |
| Transcribed locus |  | BG374448 | 1.646 | GE19392 |
| Transcribed locus | Rgs6 | BE106816 | 1.596 | GE17368 |
| Transcribed locus |  | BF406424 | 1.593 | GE18880 |
| Transcribed locus |  | BF412565 | 1.588 | GE18923 |
| Transcribed locus |  | CB582880 | 1.579 | GE1157047 |
| Transcribed locus |  | BF401410 | 1.495 | GE1195977 |
| Transcribed locus | Usp2 | AI407719 | 1.485 | GE15080 |
| Transcribed locus |  | BF523781 | 1.469 | GE1241651 |
| Transcribed locus |  | AI555172 | 1.465 | GE1226178 |
| Transcribed locus |  | BI282327 | 1.462 | GE16803 |
| Transcribed locus |  | BM391930 | 1.458 | GE14543 |
| Transcribed locus |  | AA963632 | 1.454 | GE1199475 |
| Transcribed locus |  | AW529242 | 1.449 | GE1121693 |
| Transcribed locus |  | BQ207597 | 1.448 | GE1158560 |
| Transcribed locus |  | BE121416 | 1.441 | GE1208395 |
| Transcribed locus |  | AI175820 | 1.441 | GE14412 |
| Transcribed locus |  | BF391142 | 1.438 | GE16999 |
| Transcribed locus |  | AW526033 | 1.437 | GE16247 |
| Transcribed locus |  | AI170399 | 1.436 | GE14235 |
| Transcribed locus |  | AI556052 | 1.432 | GE1284017 |
| Transcribed locus |  | BF410097 | 1.432 | GE1226413 |
| Transcribed locus |  | BQ200340 | 1.432 | GE19225 |
| Transcribed locus |  | CA507062 | 1.431 | GE1295329 |
| Transcribed locus |  | BF398263 | 1.431 | GE1242608 |
| Transcribed locus |  | AI763629 | 1.429 | GE1260105 |
| Transcribed locus | Ccnf | BF398155 | 1.428 | GE18591 |
| Transcribed locus |  | BG374430 | 1.427 | GE1103035 |
| Transcribed locus |  | BQ207985 | 1.423 | GE1292815 |
| Transcribed locus |  | BE120341 | 1.422 | GE1174508 |
| Transcribed locus |  | AI112716 | 1.42 | GE1210056 |
| Transcribed locus |  | AA924312 | 1.418 | GE1253326 |
| Transcribed locus |  | BF389961 | 1.416 | GE1100359 |
| Transcribed locus |  | BF409065 | 1.41 | GE1214127 |
| Transcribed locus |  | BF398176 | 1.406 | GE1140674 |
| Transcribed locus |  | BG379246 | 1.405 | GE1208032 |
| Transcribed locus |  | BE116110 | 1.403 | GE17523 |
| Transcribed locus |  | BI296294 | 1.401 | GE16741 |
| Transcribed locus |  | AI236270 | 0.714 | GE14921 |
| Transcribed locus | Kras2 | BE116567 | 0.714 | GE17538 |
| Transcribed locus |  | BQ208802 | 0.707 | GE1247639 |
| Transcribed locus |  | BF413839 | 0.702 | GE1187669 |
| Transcribed locus |  | BG672085 | 0.699 | GE16737 |
| Transcribed locus |  | CK602288 | 0.69 | GE1249118 |
| Transcribed locus | Acadsb | CK471598 | 0.688 | GE1149975 |
| Transcribed locus |  | AI113308 | 0.686 | GE1154971 |
| Transcribed locus |  | BG670302 | 0.683 | GE1113709 |
| Transcribed locus |  | AA891733 | 0.681 | GE12609 |
| Transcribed locus |  | CF108757 | 0.679 | GE1178018 |
| Transcribed locus |  | AW523757 | 0.677 | GE1183674 |
| Transcribed locus |  | BQ203646 | 0.636 | GE1274544 |
| Transcribed locus |  | AW527297 | 0.619 | GE1270961 |
| Transcribed locus |  | BI290092 | 0.595 | GE1192097 |
| Transcribed locus |  | BF285936 | 0.547 | GE1236150 |
| Transcribed locus |  | BM391661 | 0.512 | GE1100825 |
| Transcribed locus |  | AW534150 | 0.499 | GE1127993 |
| Transcribed locus |  | BF387213 | 0.485 | GE1209873 |
| Transcribed locus |  | AA956057 | 0.444 | GE1103429 |
| Transcribed locus, moderately similar to XP_546425.2 PREDICTED: similar to roundabout, axon guidance receptor, homolog 3 [Canis familiaris] |  | BF390065 | 0.668 | GE1287744 |
| Transcribed locus, moderately similar to XP_576460.1 PREDICTED: similar to hypothetical protein PB402898.00.0 [Rattus norvegicus] |  | BF567233 | 0.632 | GE1191980 |
| Transcribed locus, strongly similar to XP_214253.3 PREDICTED: similar to muscleblind-like 2 isoform 1 [Rattus norvegicus] |  | CF108043 | 1.452 | GE1131400 |
| Transcribed locus, strongly similar to XP_217851.3 PREDICTED: similar to mKIAA2016 protein [Rattus norvegicus] | Tfb1m | AW528895 | 1.578 | GE1111582 |
| Transcribed locus, strongly similar to XP_230811.3 PREDICTED: similar to Zinc fingers and homeoboxes 3 [Rattus norvegicus] | Zhx3 | BM391261 | 1.404 | GE1287901 |
| Transcribed locus, strongly similar to XP_238155.2 PREDICTED: similar to glucuronosyltransferase I [Rattus norvegicus] |  | BQ209786 | 1.409 | GE1137177 |
| Transcribed locus, strongly similar to XP_341653.2 PREDICTED: similar to cylindromatosis (turban tumor syndrome) [Rattus norvegicus] |  | CA505956 | 1.522 | GE18114 |
| Transcribed locus, strongly similar to XP_342572.2 PREDICTED: similar to hypothetical protein MGC47065 [Rattus norvegicus] |  | CD371670 | 0.713 | GE1273319 |
| Transcribed locus, strongly similar to XP_580039.1 PREDICTED: hypothetical protein XP_580039 [Rattus norvegicus] |  | BE120382 | 1.464 | GE1236757 |
| Transcribed locus, strongly similar to XP_580137.1 PREDICTED: hypothetical protein XP_580137 [Rattus norvegicus] |  | BM384213 | 1.497 | GE14933 |
| Transcribed locus, weakly similar to NP_001008316.1 lymphotoxin B receptor (predicted) [Rattus norvegicus] | Ltbr | BF558479 | 1.43 | GE19344 |
| Transcribed locus, weakly similar to NP_186775.1 aldose 1-epimerase [Arabidopsis thaliana] |  | AA801210 | 1.412 | GE1302110 |
| Transcribed locus, weakly similar to XP_511446.1 PREDICTED: hypothetical protein XP_511446 [Pan troglodytes] |  | AI059663 | 0.696 | GE1301605 |
| Transcribed locus, weakly similar to XP_541379.2 PREDICTED: similar to zinc finger protein 91 (HPF7, HTF10) [Canis familiaris] |  | CA508492 | 1.526 | GE1123580 |
| Transmembrane protein 38a (predicted) | Tmem38a_predicted | BG374485 | 1.447 | GE1234845 |
| Tropomyosin 1, alpha | Tpm1 | BF564902 | 1.455 | GE17114 |
| tropomyosin 1, alpha | Tma2; Tmsa; Alpha-tm | M34136 | 1.434 | GE1271208 |
| ubiquitin specific peptidase 2 | MGC93284 | NM_053774 | 1.459 | GE1176850 |
| Ubiquitin specific peptidase 3 | Usp3 | AI411205 | 1.4 | GE15246 |
| Ubiquitin specific protease 20 (predicted) | Usp20_predicted | AI013788 | 1.419 | GE13767 |
| Ubiquitin specific protease 28 (predicted) | Usp28_predicted | BF418757 | 1.4 | GE12485 |
| Ubiquitin-conjugating enzyme E2E 2 (UBC4/5 homolog, yeast) | Ube2e2 | CB793719 | 0.599 | GE1298244 |
| Ubiquitin-conjugating enzyme E2G 2 (predicted) | Ube2g2_predicted | AI713324 | 1.543 | GE15627 |
| UDP-GlcNAc:betaGal beta-1,3-N-acetylglucosaminyltransferase 1 (predicted) | B3gnt1_predicted | BG373352 | 1.435 | GE14585 |
| UI-R-A1-ex-d-08-0-UI.s1 UI-R-A1 Rattus norvegicus cDNA clone UI-R-A1-ex-d-08-0-UI 3' |  | AA955421 | 1.432 | GE1158645 |
| UI-R-BJ0-ael-b-03-0-UI.s1 UI-R-BJ0 Rattus norvegicus cDNA clone UI-R-BJ0-ael-b-03-0-UI 3', mRNA sequence. |  | AW252820 | 1.579 | GE16041 |
| UI-R-BT0-qi-a-03-0-UI.s1 UI-R-BT0 Rattus norvegicus cDNA clone UI-R-BT0-qi-a-03-0-UI 3', mRNA sequence. |  | AI145550 | 1.43 | GE1104370 |
| UI-R-BT1-ast-e-03-0-UI.s1 UI-R-BT1 Rattus norvegicus cDNA clone UI-R-BT1-ast-e-03-0-UI 3', mRNA sequence. |  | BE106814 | 1.851 | GE1155794 |
| UI-R-BT1-bnz-f-06-0-UI.s1 UI-R-BT1 Rattus norvegicus cDNA clone UI-R-BT1-bnz-f-06-0-UI 3', mRNA sequence. | Catnb | BF412807 | 1.421 | GE1202528 |
| UI-R-C1-ka-c-10-0-UI.r1 UI-R-C1 Rattus norvegicus cDNA clone UI-R-C1-ka-c-10-0-UI 5', mRNA sequence. |  | BF550907 | 1.412 | GE1120078 |
| UI-R-Y0-vm-g-09-0-UI.r1 UI-R-Y0 Rattus norvegicus cDNA clone UI-R-Y0-vm-g-09-0-UI 5', mRNA sequence. |  | BF523413 | 0.607 | GE1200965 |
| UTP14, U3 small nucleolar ribonucleoprotein, homolog A (yeast) | Utp14a | BQ199733 | 1.416 | GE1172253 |
| WAP four-disulfide core domain 2 | re4 | NM_173109 | 1.447 | GE15278 |
| WD repeat domain 41 (predicted) | Wdr41_predicted | AW521367 | 1.421 | GE16175 |
| Wolfram syndrome 1 homolog (human) | Wfs1 | BG373234 | 1.427 | GE1306383 |
| X Kell blood group precursor related family member 8 homolog | Xkr8 | AW916151 | 1.473 | GE16689 |
| X Kell blood group precursor related family member 8 homolog | Xkr8 | BF412090 | 1.404 | GE1297200 |
| Zinc finger protein 142 (clone pHZ-49) (predicted) | Znf142_predicted | BE101212 | 2.633 | GE17254 |
| Zinc finger protein 189 (predicted) | Zfp189_predicted | BG671710 | 0.552 | GE12289 |
| Zinc finger protein 216 (predicted) | Zfp216_predicted | BF401163 | 1.406 | GE1117201 |
| Zinc finger protein 629 | Zfp629 | BM388199 | 1.409 | GE1168031 |
| Zinc finger, AN1-type domain 2A | Zfand2a | CA505639 | 1.424 | GE1112268 |

**NAc**

Both (Naïve vs. 1-day and Naïve vs. 10-day)

|  |  |  | **Naïve v 1-day** | **Naïve v 10-days** | |
| --- | --- | --- | --- | --- | --- |
| **Gene** | **Alias** | **Accession #** | **Fold Change** | **Fold Change** | **Probe ID** |
| activity regulated cytoskeletal-associated protein | Arc | NM_019361 | 1.75 | 1.91 | GE20683 |
| Cadherin 22 | Cdh22 | BF388223 | 0.591 | 0.55 | GE18416 |
| Carbohydrate (chondroitin) synthase 1 (predicted) | Chsy1_predicted | CA508384 | 1.422 | 1.45 | GE15018 |
| Cartilage acidic protein 1 | Crtac1 | CK221951 | 1.454 | 1.647 | GE1285268 |
| Cd27 binding protein (Hindu God of destruction) (predicted) | Siva_predicted | BQ200239 | 1.542 | 1.559 | GE1177823 |
| early growth response 1 | Ngf1; Ngfi; NGFI-A; Krox-24; zif-268 | NM_012551 | 1.415 | 1.455 | GE1278192 |
| early growth response 2 | Krox20 | NM_053633 | 1.804 | 1.939 | GE13105 |
| Inositol 1,4,5-triphosphate receptor 1 | Itpr1 | BF410576 | 1.635 | 1.763 | GE1203008 |
| Nuclear receptor co-repressor 1 | Ncor1 | CB770816 | 0.677 | 0.672 | GE1138905 |
| nuclear receptor subfamily 4, group A, member 1 | HMR; Nur77; Ngfi-b | NM_024388 | 2.042 | 2.559 | GE1248064 |
| Nucleoredoxin (predicted) | Nxn_predicted | BF418744 | 1.485 | 1.604 | GE1218653 |
| Ribonuclease P 14 subunit homolog (human) (predicted) | Rpp14_predicted | CF110818 | 1.408 | 1.632 | GE17283 |
| Similar to CG3740-PA | LOC690000 | BQ203920 | 1.482 | 1.438 | GE16677 |
| Similar to hypothetical protein DKFZp434K1815 | LOC304396 | BF410792 | 1.424 | 1.404 | GE12704 |
| Similar to RIKEN cDNA 1110055N21 (predicted) | RGD1309370_predicted | BF416880 | 1.501 | 1.4 | GE18994 |
| Similar to RIKEN cDNA 2010012F05 (predicted) | RGD1309846_predicted | CB778957 | 0.525 | 0.485 | GE1217223 |
| sodium channel, voltage-gated, type I, beta | Scn1b | NM_017288 | 0.585 | 0.496 | GE21468 |
| solute carrier family 6 (neurotransmitter transporter, glycine), member 9 | Glyt1; GLYT-1; GLYT-1b | NM_053818 | 0.667 | 0.594 | GE21686 |
| synovial sarcoma, X breakpoint 2 interacting protein | ADIP | NM_175597 | 1.407 | 1.563 | GE15644 |
| Transcribed locus, strongly similar to XP_214253.3 PREDICTED: similar to muscleblind-like 2 isoform 1 [Rattus norvegicus] |  | CF108043 | 1.516 | 1.548 | GE1131400 |
| Transducer of ERBB2, 2 | Tob2 | BF284693 | 1.591 | 1.536 | GE18245 |
| UDP-N-acetyl-alpha-D-galactosamine:polypeptide N-acetylgalactosaminyltransferase-like 4 (predicted) | Galntl4_predicted | BG380231 | 1.404 | 1.634 | GE1157514 |
| visinin-like 1 | Nvp1; Ratnvp1; MGC105475 | NM_012686 | 0.671 | 0.661 | GE1289275 |
| Zinc finger and BTB domain containing 16 | Zbtb16 | BF409027 | 2.766 | 2.122 | GE1257656 |
| Transcribed locus |  | AW523929 | 2.277 | 3.264 | GE1235464 |
| Transcribed locus |  | BF393380 | 1.441 | 1.438 | GE1242431 |
| Transcribed locus | B130017i01rik | AI407483 | 1.433 | 1.431 | GE15072 |
| Transcribed locus |  | CF112406 | 1.413 | 1.427 | GE1188642 |
| Transcribed locus |  | BF411990 | 1.408 | 1.494 | GE1305876 |
| Transcribed locus |  | AI059369 | 0.708 | 0.575 | GE1205340 |
| Transcribed locus |  | BQ203646 | 0.653 | 0.636 | GE1274544 |
| Transcribed locus |  | BF406495 | 0.598 | 0.606 | GE1198354 |
