# Supplementary material for: Gene expression changes in the medial prefrontal cortex and nucleus accumbens following abstinence from cocaine self-administration
Source: BMC Neurosci. 2010 Feb 26;11:29. doi: 10.1186/1471-2202-11-29 (PMC2837051; doi:10.1186/1471-2202-11-29)
Supplement: Additional file 3 — Dissection schematics. Dissection diagrams. Schematics of the mPFC and NAc dissections are provided using modifications of figures from Paxinos and Watson. Numbered red lines are specific dissection cuts using visible landmarks as described in the methods. The shaded area represents the tissue collected for molecular analysis. [file 1471-2202-11-29-S3.PDF]

mPFC

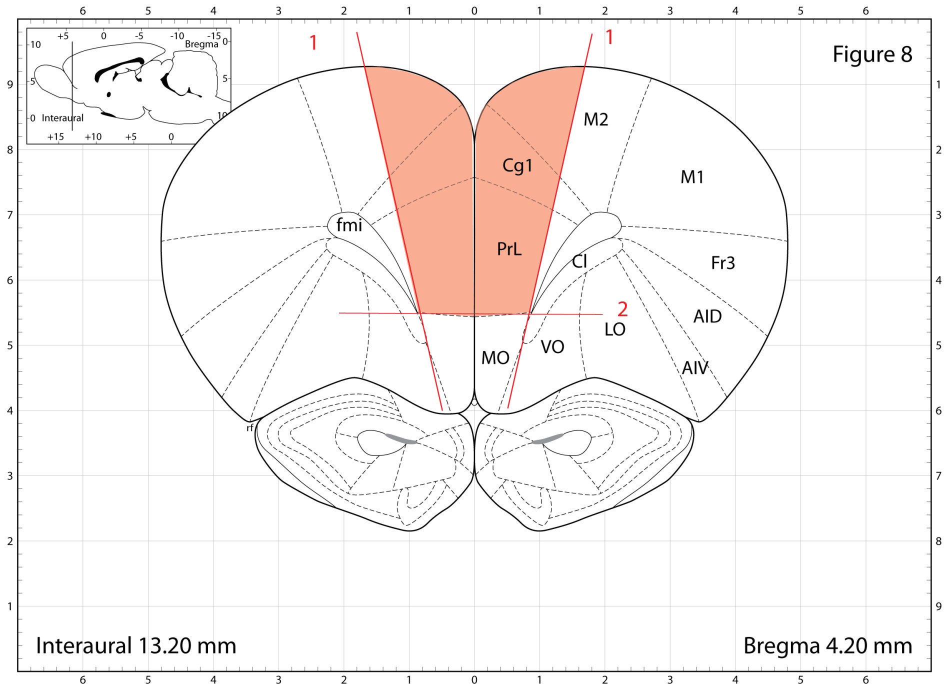

NAc

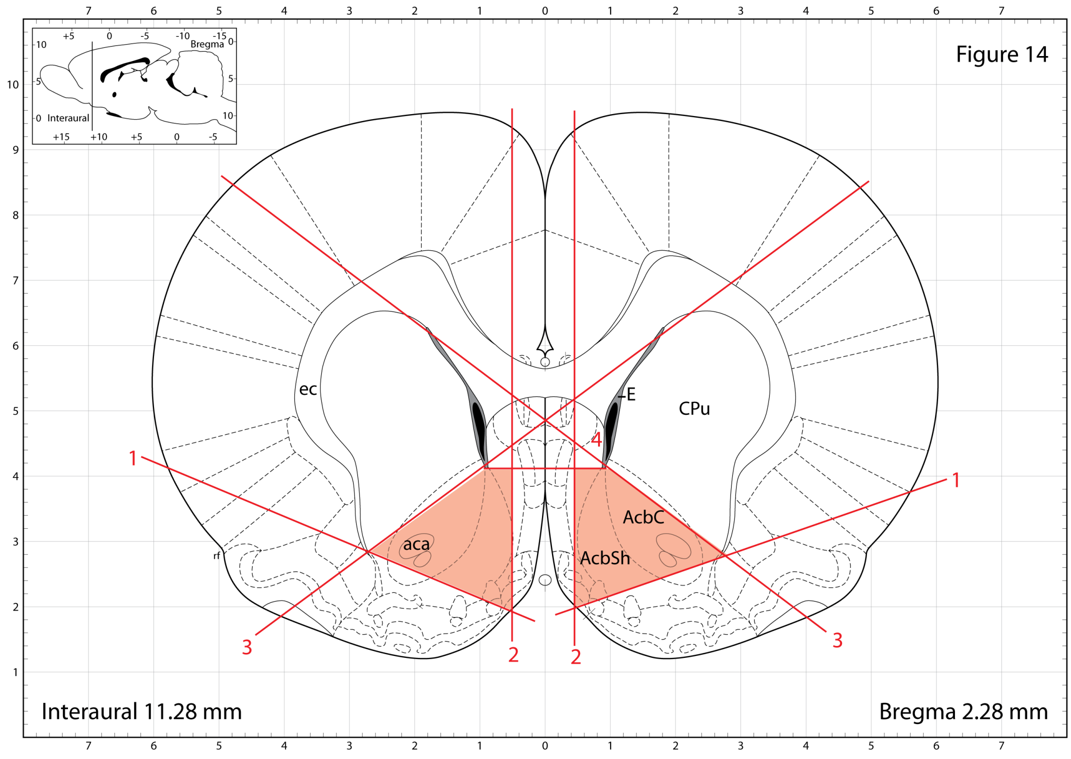

Supplementary Figure 1: Dissection diagrams. Schematics of the mPFC and NAc dissections are provided using modifications of figures from Paxinos and Watson. Numbered red lines are specific dissection cuts using visible landmarks as described in the methods. The shaded area represents the tissue collected for molecular analysis.
